# Optimized 4,5-Diarylimidazoles as Potent/Selective Inhibitors of Protein Kinase CK1δ and Their Structural Relation to p38α MAPK

**DOI:** 10.3390/molecules22040522

**Published:** 2017-03-24

**Authors:** Jakob Halekotte, Lydia Witt, Chiara Ianes, Marc Krüger, Mike Bührmann, Daniel Rauh, Christian Pichlo, Elena Brunstein, Andreas Luxenburger, Ulrich Baumann, Uwe Knippschild, Joachim Bischof, Christian Peifer

**Affiliations:** 1 Institute of Pharmacy, Christian-Albrechts-University of Kiel, Gutenbergstraße 76, D-24118 Kiel, Germany; jhalekotte@pharmazie.uni-kiel.de (J.H.); lwitt@pharmazie.uni-kiel.de (L.W.); 2 Department of General and Visceral Surgery, Ulm University Hospital, Albert-Einstein-Allee 23, D-89081 Ulm, Germany; chiara.ianes@uni-ulm.de (C.I.); marcm3012@googlemail.com (M.K.); uwe.knippschild@uniklinik-ulm.de (U.K.); 3 Institute of Chemical Biology, Dortmund University of Technology, Otto-Hahn-Straße 4a, D-44227 Dortmund, Germany; mike.buehrmann@googlemail.com (M.B.); daniel.rauh@tu-dortmund.de (D.R.); 4 Department for Chemistry, University of Cologne, Otto-Fischer-Straße 12-14, D-50674 Cologne, Germany; pichloc@uni-koeln.de (C.P.); Elena.Brunstein@uni-koeln.de (E.B.); ubaumann@uni-koeln.de (U.B.); 5 The Ferrier Research Institute, Victoria University of Wellington, Gracefield Research Centre, 69 Gracefield Road, Lower Hutt P.O. Box 33-436, New Zealand; Andreas.Luxenburger@vuw.ac.nz

**Keywords:** protein kinase CK1, formerly known as casein kinase 1, p38 MAPK, kinase inhibitors, 4,5-diaryl-imidazoles, Alzheimer’s disease, amyotrophic lateral sclerosis, familial advanced sleep phase syndrome, cancer

## Abstract

The involvement of protein kinase CK1δ in the pathogenesis of severe disorders such as Alzheimer’s disease, amyotrophic lateral sclerosis, familial advanced sleep phase syndrome, and cancer has dramatically increased interest in the development of effective small molecule inhibitors for both therapeutic application and basic research. Unfortunately, the design of CK1 isoform-specific compounds has proved to be highly complicated due to the existence of six evolutionarily conserved human CK1 members that possess similar, different, or even opposite physiological and pathophysiological implications. Consequently, only few potent and selective CK1δ inhibitors have been reported so far and structurally divergent approaches are urgently needed in order to establish SAR that might enable complete discrimination of CK1 isoforms and related p38α MAPK. In this study we report on design and characterization of optimized 4,5-diarylimidazoles as highly effective ATP-competitive inhibitors of CK1δ with compounds **11b** (IC_50_ CK1δ = 4 nM, IC_50_ CK1ε = 25 nM), **12a** (IC_50_ CK1δ = 19 nM, IC_50_ CK1ε = 227 nM), and **16b** (IC_50_ CK1δ = 8 nM, IC_50_ CK1ε = 81 nM) being among the most potent CK1δ-targeting agents published to date. Inhibitor compound **11b**, displaying potential as a pharmacological tool, has further been profiled over a panel of 321 protein kinases exhibiting high selectivity. Cellular efficacy has been evaluated in human pancreatic cancer cell lines Colo357 (EC_50_ = 3.5 µM) and Panc89 (EC_50_ = 1.5 µM). SAR is substantiated by X-ray crystallographic analysis of **16b** in CK1δ and **11b** in p38α.

## 1. Introduction

**Figure 1:**
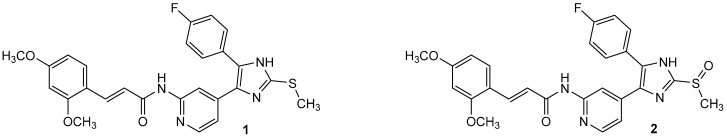
ATP-competitive dual specific inhibitors **1** and **2** of CK1δ/ε and p38α MAPK.

Protein kinase CK1δ is a member of the ubiquitously expressed and constitutively active Ser/Thr-specific CK1 (formerly known as casein kinase 1) family which comprises the six human isoforms α, γ1, γ2, γ3, δ, and ε, together with their closest relatives the tau tubulin kinases 1 and 2 (TTBK1/2) and vaccinia-related kinases 1-3 (VRK1–3) [1,2,3]. Despite CK1 being evolutionarily highly conserved within their catalytic domains, all isoforms differ significantly in the length and the primary structure of their regulatory N- and C-terminal regions. Among them, isoforms δ and ε display the highest consensus, with a 98% sequence identity within their catalytic domain and at least 40% homology within their autoregulatory C-terminal domains [2,3,4,5]. Pathophysiologically, identification of mutations within the coding region of CK1δ as well as deregulation of CK1δ expression and/or activity levels as important determinants in development and progression of severe human disorders such as Alzheimer’s disease (AD) [2,6,7,8], amyotrophic lateral sclerosis (ALS) [9], familial advanced sleep phase syndrome (FASPS) [10], and cancer [2,5,11,12,13,14,15,16,17,18,19] has dramatically increased interest in the development of potent and selective small molecule kinase inhibitors for both therapeutic approaches and basic research. However, the existence of paralogous CK1 isoforms that possess similar, different, or even opposite physiological and pathophysiological implications render the design of suitable candidate molecules that target CK1δ in an ideally isoform-dependent manner enormously difficult. The most extensively used and characterized CK1 inhibitor to date, IC261, moderately inhibits CK1 isoforms δ and ε (IC_50_ CK1δ/ε = 1 µM, IC_50_ CK1α = 10 µM) and proved valuable in diverse pharmacological studies [16,20,21,22]. Furthermore, IC261 even revealed therapeutic potential for the treatment of pancreatic cancer in a subcutaneous mouse xeno-transplantation model, despite the fact that the inhibitor is active against several other targets, including tubulin polymerization and ion channels [16,22,23,24]. In addition, few further compounds have been reported as CK1 inhibitors, mainly those which are commonly referred to as “linear” [25,26] and “tear-drop”-like binders [27] with respect to their three-dimensional structure. Among the latter, a promising 4,5-diarylimidazole-based inhibitor **1**, originally designed as an inhibitor of p38α MAPK, was revealed in 2009 by Peifer et al. to inhibit CK1δ/ε with IC_50_ values in the low nanomolar range (**1** IC_50_ CK1δ = 5 nM, **1** IC_50_ CK1ε = 73 nM) [28]. Interestingly, sulfoxidation of thioether **1** leading to sulfoxide **2** significantly enhanced discrimination of highly related isoforms δ and ε to at least 40-fold while preserving good potency for CK1δ (**2** IC_50_ CK1δ = 11 nM, **2** IC_50_ CK1ε = 447 nM, Figure 1) [28]. Unfortunately, the reduced chemical stability of **1** and **2** in solution due to *E*/*Z*-isomerization and the presence of a *Michael* acceptor moiety in the cinnamic acid side chain are responsible for their limited usability in vitro and in vivo.

The present follow-up [28] study reports on the optimization of lead structures **1** and **2**, respectively, leading to stable novel inhibitors of CK1δ/ε with IC_50_ values in the low nanomolar range. The optimization strategy followed a well-established procedure in medicinal chemistry including in silico design, hit synthesis, and in vitro biological evaluation [29,30].

## 2. Results and Discussion

### 2.1. Molecular Modeling

**Figure 2:**
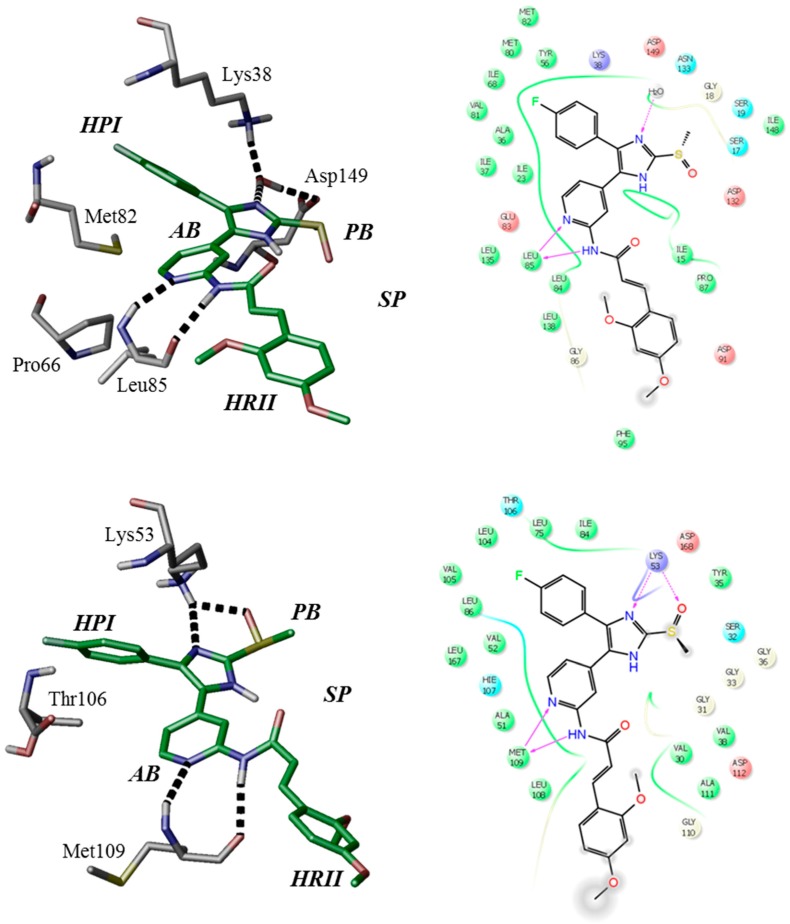
Modeled binding modes of **2** in CK1δ (top, pdb 3UZP [31]) and p38α (bottom, pdb 1BMK [33]) ATP-binding pockets. Key amino acid residues and ligand-active site interactions are shown. **Left**: in accordance to Traxler et al. [34], the ATP-binding pocket of protein kinases ought to be subdivided into hydrophobic pocket I (*HPI*), hydrophobic region II (*HRII)*, adenine-binding region (*AB*), phosphate-binding region (*PB*), and sugar pocket (*SP*). **Right**: 2D ligand interaction diagrams.

The binding modes of ATP-competitive type-I inhibitors **1** and **2** in CK1δ and p38α have been postulated based on structure-based molecular modeling (Figure 2) [28]. Comparable to poses of similar “tear-drop”-like binders (e.g., pdb 3UZP [31]) two hydrogen bonds are formed between the 2-amino-pyridine moiety and CK1δ hinge residue Leu85. The positive mesomeric electron donating effect of the amino group in *ortho*-position of the pyridine nitrogen positively influences electron density and thus the H-bond acceptor strength at the pyridine-*N* [32].

Furthermore, core catalytic residues Lys38 and Asp149 [13,31] coordinate a structural water within the catalytic cleft which donates another hydrogen bond towards an imidazole nitrogen of the respective inhibitor. Gatekeeper residue Met82 is rotated by 180° towards Pro66 [13,31], thereby permitting access to the hydrophobic pocket I (*selectivity pocket*, *HPI*) [2,34] which is ideally occupied by the 4-fluorophenyl moiety [31,35]. Molecular modeling further suggests five-membered heterocycles to dictate an ideal angle for positioning of the vicinal aryl moieties within the ATP-binding pocket of CK1δ [36]. The (*E*)-configured cinnamic acid side chain of **1** and **2** extends into the spacious solvent-exposed hydrophobic region II (*affinity pocket*, *HRII*) [2,34]. Modeling calculations further considered different di- or trimethoxyphenyl substitution pattern optimal within this region as they enable flexible occupation of hydrophobic surfaces and shielding deeper cavities of the ATP-binding pocket from surrounding water, thus entailing increased enthalpic contribution of buried hydrogen bonds. Analogous binding poses have been achieved regarding p38α with the bidentate hinge-binding moiety addressing Met109 and the imidazole-*N* accepting a hydrogen bond from Lys53. Rotation of the smaller gatekeeper residue Thr106, however, does not seem necessary in order to occupy *HPI*.

**Scheme 1:**
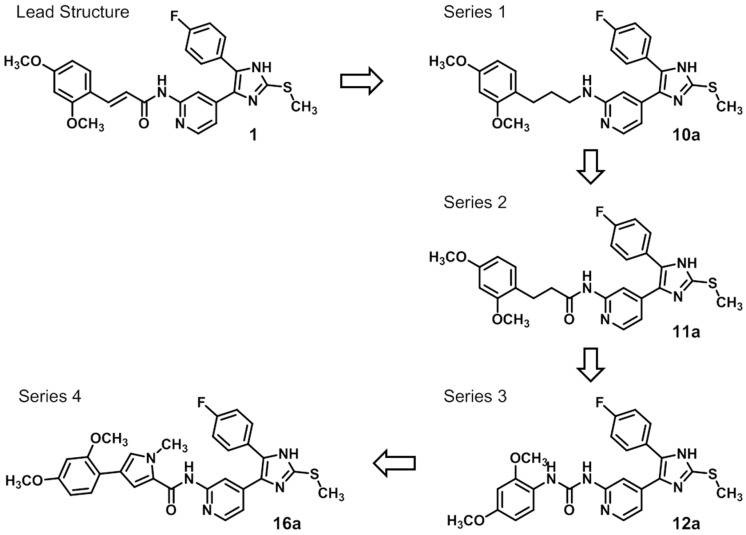
Structural considerations based on molecular modeling leading to lead structure **1** derivatives **10a** (series 1), **11a** (series 2), **12a** (series 3), and **16a** (series 4).

The binding modes described above are based on (*E*)-configured cinnamic moieties of **1** and **2**, respectively. In contrast, molecular modeling of the respective (*Z*)-configurations did not result in plausible binding modes (not shown). Accordingly, computational analysis assume these (*Z*)-stereoisomers to be less bioactive. Furthermore, the acrylamide *Michael* acceptor moiety of the cinnamic acid side chain was considered responsible for the observed chemical instability of **1** and **2** in solution. In line with this notion, within a short period of time after preparing a solution of **1** and **2** in DMSO a HPLC analysis showed an increasing number of not identifiable degradation products. Consequently, our primary goal towards an optimized inhibitor was to gain chemical stability. Thus, having identified the cinnamic side chain to be responsible for the chemical instability issue we aimed towards stable side chains attached at the validated 2-aminopyridine core moiety. By these modifications we set out to explore the respective hydrophobic region II formerly occupied by the cinnamic acid moiety. At the same time, both potency and selectivity for CK1δ were taken into account. Therefore, in our systematic approach four structurally divergent series of inhibitors with variable side chains (Scheme 1) have been designed based on the following considerations.

First, removal of both the planar (sp^2^) π-bond and carbonyl group in **1** and **2** led to respective sp^3^ hybridized 3-(2,4-dimethoxyphenyl)propanamine **10a** and derivatives (series 1). However, at this position of the ligand, additional degrees of freedom and enhanced conformational flexibility are typically accompanied by losses of both potency and selectivity; Second, maintaining the amide function but formally reducing the π-bond resulted in presumably stable and potent 3-(2,4-dimethoxyphenyl)propionic amide derivatives (e.g., **11a**, series 2). Third, a carbamide moiety in **12a** and derivatives (series 3) might enable an additional hydrogen bond towards hinge Leu85 and therefore could account for enthalpic binding energy gains. The additional fixation was further suggested to exploit different folding of related CK1δ, CK1ε, and p38α within range of the hinge region and thus to be a key parameter for triggering inhibitor selectivity. And fourth, fixing the (*E*)-configuration of cinnamic amides **1** and **2** within five-membered heterocycles led to 4-(2,4-dimethoxyphenyl)-1-methyl-1*H*-pyrrole-2-carboxylic amide (**16a**, series 4). Taken together, by our compound design concept in the side chain *Michael* acceptor characteristics and inactive (*Z*)-isomers were eliminated while potentially conserving beneficial impacts regarding potency and selectivity.

Based on the inhibitor categories described above we generated a virtual set of compounds being subsequently processed in a LigPrep/Glide docking campaign using CK1δ (pdb 3UZP [31]) and p38α (pdb 1BMK [33]) protein structures. Thereby we focused on variation of side chains addressing the *HRII* while maintaining the fixed 4,5-diaryl-imidazole pharmacophore. This included different sets of substituted lipophilic and mainly sterically demanding moieties in order to exploit this region. As methoxy-substituents were assumed most favorable in this context, efforts have been devoted to methoxy-screenings investigating different substitution patterns.

### 2.2. Synthesis

**Scheme 2:**
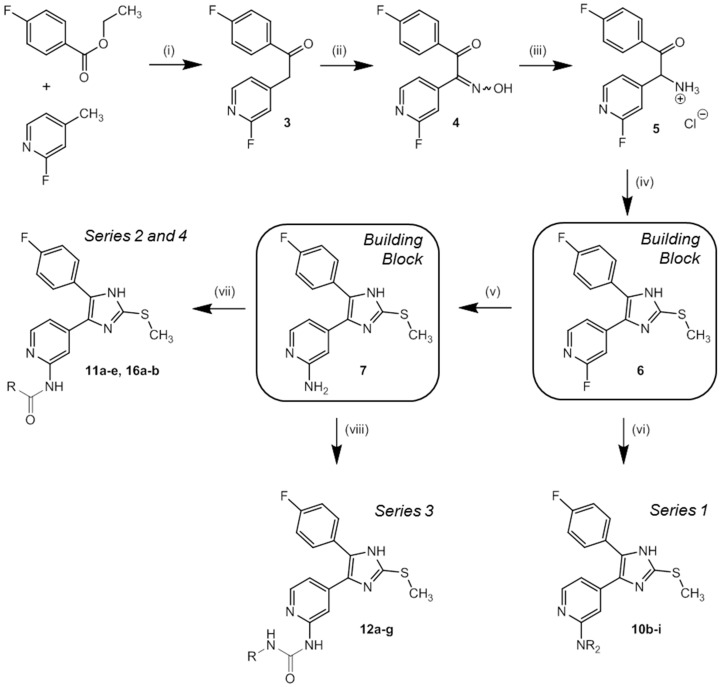
Synthesis of key building blocks **6** and **7** as well as inhibitors **10b**–**i** (series 1), **11a**–**e** (series 2), **12a**–**g** (series 3), and **16a**–**b** (series 4). *Reagents and Conditions*: (i) NaHMDS, THF, 2 h 0 °C, 1 h r.t.; (ii) CH_3_CHOOH, NaNO_2_, 1 h 0 °C, 3.5 h r.t.; (iii) H_2_, Pd/C, HCl-saturated 2-propanol, 12 h r.t.; (iv) methyl thiocyanate, DMF, 45 min reflux, 45 min r.t.; (v) NH_3_, 20–30 bar, 18 h 180 °C; (vi) HNR_2_, 12 h 160 °C; (vii) carboxylic acid, CDI or PyBOP/DIPEA, DMF, 12 h 110 °C; (viii) isocyanate, DIPEA, DMF, 12 h r.t.

In order to effectively synthesize the designed and top ranked hits from docking, a straightforward five-step procedure towards the building blocks 2-fluoro-4-(5-(4-fluorophenyl)-2-(methylthio)-1*H*-imidazol-4-yl)pyridine (**6**) and 4-(5-(4-fluorophenyl)-2-(methylthio)-1*H*-imidazol-4-yl)pyridine-2-amine (**7**) was established in accordance to literature protocols [37,38]. In the final step, by substitution of key compounds **6** and **7,** variations of side chains were introduced and thus four compound series were prepared (Scheme 2).

**Scheme 3:**
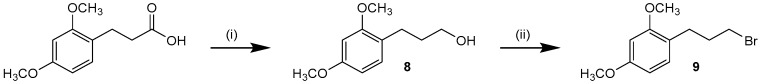
Synthesis of 1-(3-bromopropyl)-2,4-dimethoxybenzene (**9**). *Reagents and Conditions*: (i) LiAlH_4_, THF, 1 h r.t.; (ii) NBS, PPh_3_, DCM, 2 h r.t.

Slightly deviating from the procedure depicted in Scheme 2, pyridine-2-amine **10a** has been synthesized by a S_N_2 reaction of single Boc-protected 2-amino-4-methylpyridine and 1-(3-bromopropyl)-2,4-dimethoxybenzene (**9**) followed by subsequent formation of the 4,5-diaryl-imidazole scaffold using the procedure developed for synthesis of **6**. Alkyl halide **9** was synthesized from 3-(2,4-dimethoxyphenyl)propionic acid by reduction [39] followed by an Appel reaction using triphenylphosphine and *N*-bromosuccinimide (Scheme 3) [40]. Detailed information about the synthesis of **10a** is presented in the Supporting Information.

Series 1 pyridine-2-amines **10b**–**f** and piperazines **10g**–**i** were prepared by a Tschitschibabin-like nucleophilic substitution [32,38]. Therefore, 2-fluoropyridine **6** was dissolved or suspended in an excess of the appropriate amine or piperazine and heated to 160 °C (Scheme 2). In general, in this reaction primary amines accounted for better yields. 

Syntheses of series 2 included the reaction of building block **7** with CDI-activated differently di- or trimethoxy-substituted 3-phenylpropionic acids to afford amides **11a**–**e**. The poor nucleophilic character of **7**, however, required heating to 110 °C in order to achieve suitable reactivity (Scheme 2) [28].

In contrast, highly reactive isocyanates readily acylated **7** at room temperature in terms of a Wöhler-like synthesis leading to series 3 carbamide derivatives **12a**–**g** (Scheme 2) [41]. Noteworthy, addition of Hünig’s base led to increased yields and reactions had to be performed under protective gas atmosphere in order to prevent formation of carbamide dimers.

**Scheme 4:**
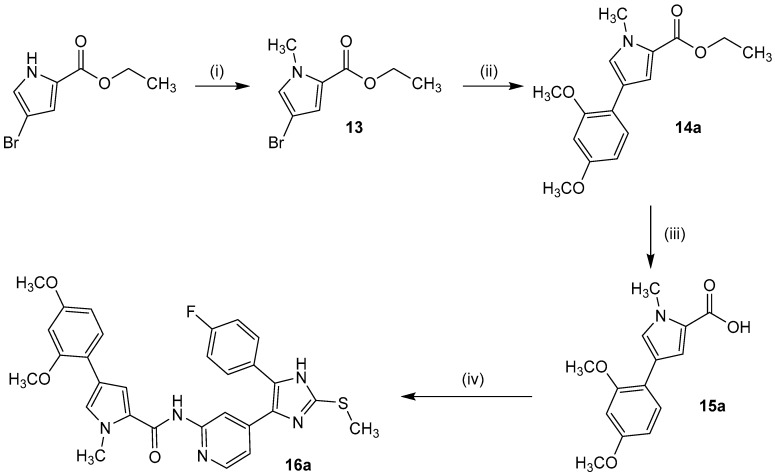
Synthesis of 4-(2,4-dimethoxyphenyl)-1-methyl-1*H*-pyrrole-2-carboxylic acid (**15a**). *Reagents and Conditions*: (i) NaH, DMF, 20 min 0 °C, CH_3_I, 15 min 0 °C, 2.5 h r.t.; (ii) 2,4-dimethoxyphenyl boronic acid, Pd(PPh_3_)_4_, aq. NaHCO_3_, DMF, 4 h reflux, 12 h r.t.; (iii) aq. NaOH, THF/methanol, 5 h 50 °C, 12 h r.t., (iv) DIPEA, DMF, 30 min r.t., **7**, 12 h 110 °C.

Series 4 precursor 4-(2,4-dimethoxy-phenyl)pyrrole-2-carboxylic acid was prepared in a three step synthesis starting from ethyl 4-bromo-1*H*-pyrrole-2-caboxylate [42]. Methylation of the pyrrole nitrogen using methyl iodide was followed by Suzuki cross-coupling with (2,4-dimethoxyphenyl)boronic acid and subsequent simple ester hydrolysis in diluted alkali. The 2,5-dimethoxyphenyl side chain derivative **15b** has been synthesized analogously. Finally, **16a**–**b** were obtained by PyBOP-supported amide coupling with building block **7** under elevated temperature (Scheme 4).

**Scheme 5:**
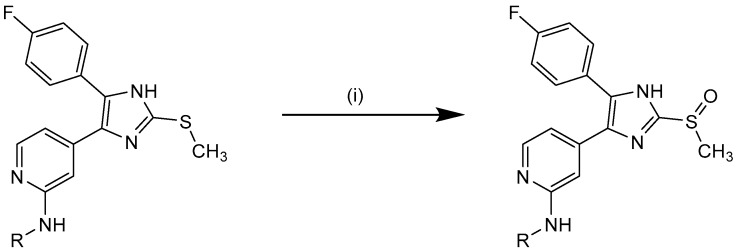
Synthesis of sulfoxides. *Reagents and Conditions*: (i) Oxone^®^ (potassium peroxomonosulfate), THF/H_2_O, 30 min–2 h 0 °C.

As mentioned above, the respective sulfoxides might possess interesting potential regarding selectivity. Therefore, oxidation of selected compounds was performed in accordance to literature using Oxone^®^ at 0 °C or at ambient temperature (Scheme 5). During thioether oxidation it was essential to strictly monitor reaction progress for quenching at the sulfoxide level [28]. Accordingly, sulfoxidation was performed for compounds **10c**–**d**, **11a**–**b**, **12a**, **12c**–**g**, and **16a** leading to **10j**–**k** (series 1), **11f**–**g** (series 2), **12h**–**m** (series 3), and **16c** (series 4). It has to be noted that sulfoxides are chiral compounds and we are always referring to the racemate. However, docking analysis did not reveal differences between the enantiomers. In the literature the racemate is reported for this inhibitor class with only one exception, where the *R*-configuration was observed to possess increased affinity for p38α MAPK [43].

All synthesized target compounds **10a**–**k**, **11a**–**g**, **12a**–**m**, **16a**–**c** are listed in Table 1. They were stable in DMSO solution at room temperature without detectable degradation over a period of 72 h by HPLC analysis.

### 2.3. Kinase Assays and IC_50_ Determination

Compounds **10a**–**k** (series 1), **11a**–**g** (series 2), **12a**–**m** (series 3), and **16a**–**c** (series 4) have initially been screened for their ability to inhibit the activity of CK1δ and CK1ε in an in vitro kinase assay at a concentration of 10 µM. Inhibitors exhibiting promising effects were further subjected to IC_50_ value determination (Table 2). In addition, IC_50_ values regarding inhibition of CK1δ and p38α MAPK have commercially been obtained from ProQinase GmbH (Freiburg, Germany) for **11b**, **12a**, and **16b** as these were the most potent representatives of their respective series (Table 3). In comparison to our data, IC_50_ values measured by ProQinase appear slightly lower due to the different assay setup such as the ATP concentration used (compare Experimental Section/Supporting Information and www.proqinase.com).

Fortunately, potent CK1δ inhibition and appropriate SAR correlated with the calculated binding modes for the majority of compounds. Especially **11b**, **16a**, and **16b** exhibited excellent efficacy with IC_50_ values in the single-digit nanomolar range in standardized kinase assays and are therefore among the most potent CK1δ inhibitors reported to date. In general, loss of potency for series 1 inhibitors when compared to lead structure **1** has been observed in vitro: while **1** has an IC_50_ for CK1δ of 5 nM [28] a side chain without both carbonyl group and π-bond resulted in considerably decreased inhibition (e.g., series 1, **10a** IC_50_ CK1δ = 386 nM). In contrast, compounds possessing the carbonyl group but without π-bond afforded chemically stable series 2 (e.g., **11a** IC_50_ CK1δ = 20 nM, IC_50_ CK1ε = 129 nM) and thereby restored potency almost to the level reported for lead **1** (IC_50_ CK1δ = 5 nM, IC_50_ CK1ε = 73 nM). Isoform selectivity regarding CK1ε, however, seemed to be slightly decreased. Consequently, the π-bond of lead **1** can be identified to be responsible for compound instability, while the carbonyl depicts an important determinant concerning potency. In addition, series 3 and 4 both provided potent inhibitors of CK1δ with IC_50_ values in the low nanomolar range. However, the aimed impact on CK1δ/ε isoform selectivity by rigidification of the double bond in the (*E*)-configuration has not been fully achieved. Unfortunately, tridentate hinge binding carbamide derivatives **12a**–**m** (series 3) were actually limited by their poor solubility in vitro.

Oxidation of the exocyclic sulfur at the imidazole-2-position of thioether **1** to afford sulfoxide **2** reflects in vivo metabolization by phase I enzymes [44]. Interestingly, this sulfoxidation increased CK1δ/ε isoform selectivity in one reported previously study [28]. In our hands, however, this effect could not be confirmed for the newly designed and synthesized set of inhibitors as oxidation only slightly altered potency, though without significantly affecting isoform selectivity. Nevertheless, sulfoxidized compounds remained potent inhibitors of CK1δ with IC_50_ values in the low nanomolar range. This indicates that these agents will retain activity despite metabolization.

### 2.4. X-ray Analysis of Binding Modes in CK1δ and p38α

In order to verify modeled binding modes and to obtain further insights regarding ligand-protein interactions determining selectivity we set out to co-crystallize potent inhibitors of series 2, 3, and 4 (**11b**, **12a**, and **16b**) in CK1δ. In line with this notion, a ligand-protein structure of **16b** could be co-crystallized with a C-terminally truncated CK1δ construct and the complex structure was determined at 2.0 Å resolution. Unfortunately, co-crystallization of **11b** and **12a** failed experimentally.

**Figure 3:**
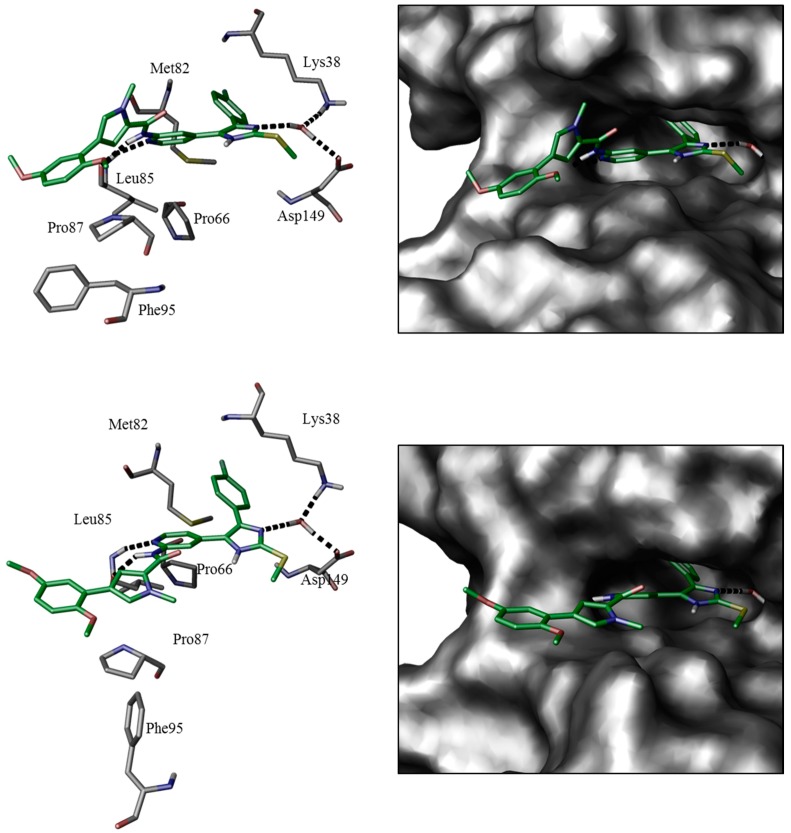
Similar binding modes of 16b in CK1δ have been gained by co-crystallization (**top**, pdb 5MQV) and molecular modeling (**bottom**, pdb 3UZP [31]). The poses are presented by key residues and hydrogen bond interactions (**left**) and Connolly molecular surface (**right**).

**Figure 4:**
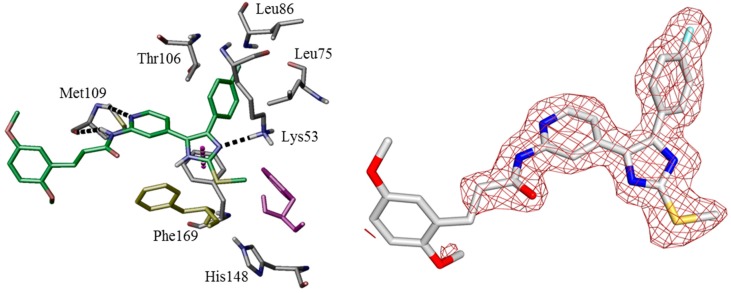
Co-crystallization of 11b in p38α MAPK (pdb 5ML5). The binding mode is presented by key residues and hydrogen bond interactions as well as π-π-stacking (**left**). DFG Phe169 is stabilized in an intermediate state between active DFG-in (purple, pdb 1BMK [33]) and inactive DFG-out (yellow, pdb 1WBT [52]) conformation. DFG-in/out states were represented by structure alignment using Schrödinger software. The inhibitor side chain addressing *HRII* has not been definitely resolvable in terms of electron density (**right**, generated with PyMOL).

Additionally, overall most active agent **11b** has been co-crystallized with p38α MAPK at 1.9 Å resolution. In fact, crystallographic data largely confirmed the calculated binding pose of **16b** within the active site of CK1δ (Figure 3). As determined by X-ray analysis the 4-fluorophenyl moiety deeply penetrates into *HPI*, enabled by rotation of gatekeeper Met82 towards Pro66. The bidentate hinge-binding 2-aminopyridine motif forms two hydrogen bonds towards Leu85. Furthermore, Lys38 and Asp149 coordinate a water molecule which donates another hydrogen bond accepted by an imidazole nitrogen of **16b**. *HRII*, however, is addressed by bulky 4-(2,5-dimethoxyphenyl)-1-methyl-1*H*-pyrrole-2-carboxamide, probably displacing energetically unfavorable water molecules and shielding deeper hydrophobic cavities of the binding pocket. Although the 2,5-dimethoxyphenyl moiety herein mainly interacts with Pro87 by π-aliphatic-stacking, electron density has also been observed within range of Phe95 which can be interpreted by different conformers of **16b**, thus indicating additional interaction. The complex of **11b** in p38α MAPK predominantly confirmed the expected 4,5-diarylimidazole core fitting, similar to CK1δ, with gatekeeper Thr106-lined *HPI* occupied by the 4-fluorophenyl moiety and the 2-aminopyridine acting as bidentate hinge binder addressing Met109. The ATP-binding pocket in this structure does not contain structural water molecules and Lys53 directly interacts with **11b** imidazole nitrogen by formation of a hydrogen bond. Interestingly, DFG-motif Phe169 is coordinated between **11b** imidazole-*N* and Lys53 by π–π- and, most likely, π-cation-stacking, respectively, thereby interrupting the hydrophobic spine consisting of Leu75, Leu86, Phe169, and His148 [45,46,47]. This pose is stabilizing the kinase in an intermediate conformation between DFG-in and DFG-out (*DFG-in-between*) [48], thereby showing several characteristics similar to type-I½-like inhibition of p38α MAPK [49,50], although molecular alignMent studies may suggest the DFG-in state most likely to be inhibited by **11b** as well. However, the DFG-out conformation can be assumed to be sterically prohibited (Figure 4, left). Interestingly, the 3-(2,5-dimethoxyphenyl)propionic amide side chain of **11b** addressing *HRII* has not been definitely resolvable by X-ray analysis in terms of electron density (Figure 4, right). In fact, this is in agreement with our hypothesis postulating high flexibility of this ligand part within *HRII*. In line with this notion, we originally designed bulky inhibitor side chains to suboptimal fit into less extensive hydrophobic region II of p38α MAPK, in turn allowing higher affinity for CK1δ. Thus, in that way paying enthalpic and entropic penalty should result in decreased affinity for p38α MAPK when compared to the situation in CK1δ [51]. However, in contrast to our hypothesis inhibitors actually showing the bulky side chain, e.g., **11b**, **12a**, and **16b**, were determined to be nanomolar inhibitors of p38α MAPK as well as of CK1δ. This finding suggests that—despite bulky side chains—the core 4,5-diaryl-imidazole scaffold addressing both ATP sites of CK1δ and p38α is able to determine high affinity for these kinases.

### 2.5. Selectivity Profiling of ***11b***

**Figure 5:**
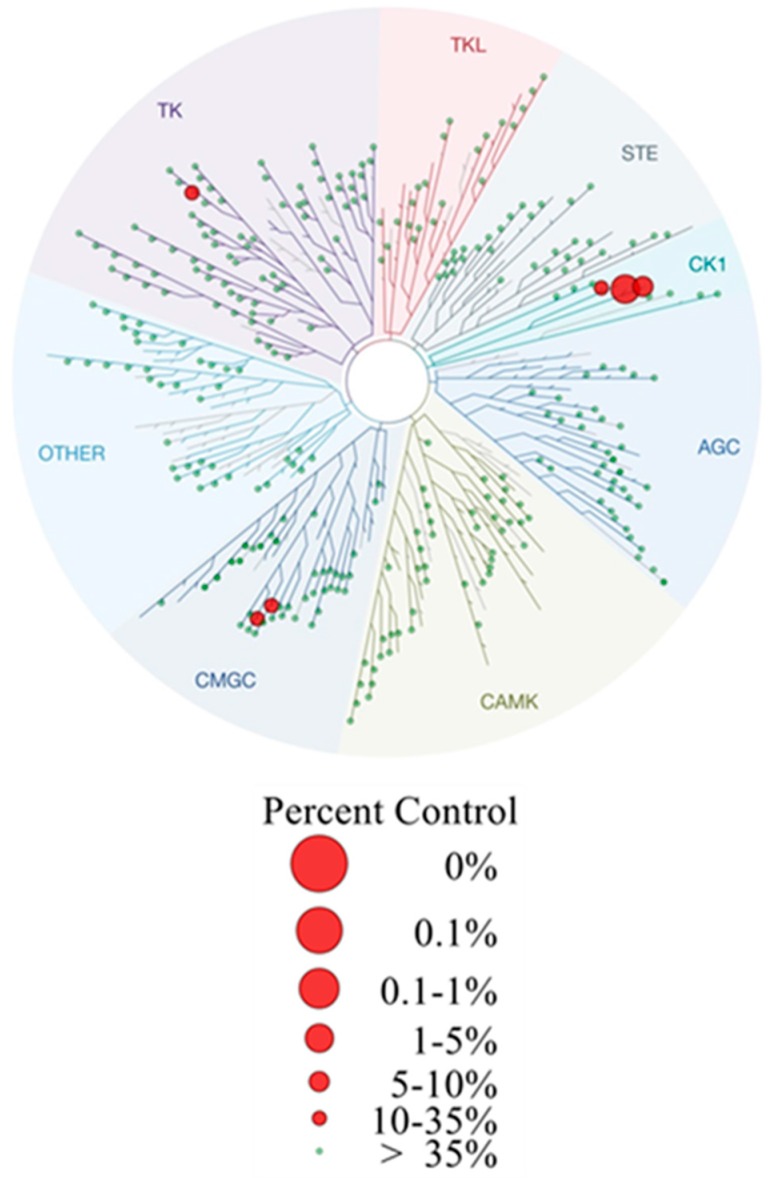
Dendrogram representation of selectivity profiling of **11b** screened over 321 protein kinases at a concentration of 100 nM (ProQinase GmbH, Freiburg, Germany). Residual activity was determined compared to DMSO control. The S-Score(50) of **11b** is 0.027. The dendrogram was generated utilizing TREEspot Software Tool, DISCOVERX CORPORATION 2010. Corresponding raw data are given in Supplementary Table S5.

In order to further characterize potent inhibitor **11b** selectivity profiling has been performed at a concentration of 100 nM in a panel of 321 protein kinases (ProQinase GmbH). These data revealed high selectivity of **11b** for CK1δ hitting only six additional kinases with residual activities less than 50% apart from CK1δ (residual activity = 3%) and CK1ε (residual activity = 7%, CK1 isoforms γ1–3 remained unaffected). Among these, CK1α (residual activity = 26%) and p38α MAPK (residual activity = 22%) were found as prominent targets. Also the highly related kinases JNK2 (residual activity = 21%), JNK3 (residual activity = 37%), and RIPK2 (residual activity = 45%) were identified as additional hits. Furthermore, Tyr-specific kinase LCK (residual activity = 26%) was inhibited (Figure 5 and Supporting Information). Consequently, **11b** represents an agent highly selective for CK1δ within the range of its IC_50_ value of 4 nM with an overall selectivity score S(50) of 0.027. The S-Score(50) has been calculated in accordance to Karaman et al. [53] describing the portion of kinases with a residual activity >50% in relation to all tested kinases included in this project.

### 2.6. Cellular Assays and EC_50_ Determination

Synthesized compounds have further been evaluated regarding their efficacy in cellular systems in MTT viability assays using colorectal HT-29 or pancreatic Colo357, Panc89, Panc-1, and MiaPaCa-2 carcinoma cell lines. The cell lines were chosen as they are reportedly overexpressing CK1δ and CK1ε and exhibit resistance against a variety of chemotherapeutic agents [12,15,16]. In general, amines **10a**–**k** (series 1) were rather ineffective, even at an elevated concentration of 20 µM with **10e** being most active exhibiting an EC_50_ value of 9.3 µM in HT-29 cells (data can be found in Supporting Information). For the subsequently designed compound series EC_50_ data were only obtained for compounds which showed the most promising inhibition of CK1δ in vitro. Inhibitors from series 2 (**11a**–**e**) showed significant increases of potency, presumably referable to the amide carbonyl group which has already been reported to beneficially affect metabolic stability [44]. As expected, **16a**–**b** (series 4) were only slightly less active. In contrast, carbamides **12a**–**k** (series 3) were only moderately active, presumably suffering from poor solubility. Among all series, **11b** again proved the most effective agent with EC_50_ values of 3.5 µM and 1.5 µM in Colo357 and Panc89 cells, respectively (determined EC_50_ values can be found in Table 4).

If the EC_50_ values determined in cell-based assays are compared with in vitro determined IC_50_ data, massive differences can be observed and compounds which appeared to be extremely potent are apparently less efficient in the treatment of living cells. This difference is compound and cell line dependent, can be due to different reasons, and has already been documented in previous reports [26,54]. Firstly, limited cell permeability of small molecule inhibitors may limit their cellular uptake resulting in lower potency; Secondly, once compounds successfully crossed the cell membrane, ATP-competitive inhibitors must compete with high intracellular ATP levels leading to a discrepancy between IC_50_ values determined by enzymatic versus cellular assays. Additionally, time of binding in the ATP pocket and drug export systems can reduce the inhibitory effect. Therefore, cell-based assays are essential in order to validate the inhibitory effects of newlx identified small molecule inhibitors.

## 3. Materials and Methods

### 3.1. Molecular Modeling

Molecular modeling was performed on a DELL Precision T5500 eight core workstation. For visualization Maestro, version 10.6, 2016 (Schrödinger LLC, New York, NY, USA) was used. Protein crystal structures were prepared prior to docking by the Protein Preparation Wizard [55] synchronizing the following modules: Epik, version 3.6, 2016 [56]; Impact, version 7.1, 2016; Prime, version 4.4, 2016 [57]. In order to achieve high Enrichment-factors, the common refinement protocol by Sastry et al. [55] has been adjusted: the process involved assignment of bond orders, addition of hydrogen atoms, identification of disulfide bonds, and the conversion of artifical selenomethionines to methionines (default settings). Missing side chains were filled in using Prime. Missing loops have not been detected. Water molecules beyond 5 Å from hetero atoms have been deleted automatically. H-bond optimization was performed in a standard sampling, the Root-mean-square deviation for atomic positions cutoff for heavy atoms in subsequent protein minimization was set to 0.3 Å

Ligands were prepared to generate energetically minimized three-dimensional coordinates with an extended cutoff by MacroModel, version 11.2, 2016 (Schrödinger LLC). Ionization and tautomeric states were estimated at pH 7 ± 2 by LigPrep, version 3.8, 2016 (Schrödinger LLC) [55], utilizing Hammet and Taft methodology-based Epik [56]. Additionally, Epik state penalties (kcal∙mol^−1^) were calculated for each ligand to quantify the energetic cost for state transition in solution [55]. In order to indicate ligand flexibility, up to 50 bioactive conformers per ligand were identified and prioritized utilizing the conformational search module in the fast mode (ConfGen, version 3.6, 2016, Schrödinger LLC) [58]. Receptor grid generation was generated with Glide, version 7.1, 2016 (Schrödinger LLC) [59]. For ligand docking and screening the Glide XP workflow was used [59]. Energetically minimized ligand conformations were docked into the active site of the protein; possible binding poses were determined and subsequently ranked based on their calculated binding affinities.

### 3.2. Chemistry

Infrared spectra were recorded on an IRAffinity-1S FTIR-spectrometer (Shimadzu Europa GmbH, Hannover, Germany). NMR spectra were recorded on an Avance III 300 spectrometer, tempered at 298 K: ^1^H (300 MHz), ^13^C-NMR (75 MHz) (Bruker Daltonik GmbH, Bremen, Germany). The data is reported as follows: chemical shift in ppm from tetramethylsilane (TMS) as external standard, multiplicity and coupling constant *J* (Hz). Spectra were either referenced to TMS or internal DMSO-*d*_6_ (^1^H-NMR δ 2.50) and internal DMSO-*d*_6_ (^13^C-NMR δ 39.52) or internal CHCl_3_ (^1^H-NMR δ 7.26) and internal CDCl_3_ (^13^C-NMR δ 77.00). The following NMR abbreviations have been used: b (broad), s (singlet), d (doublet), t (triplet), m (unresolved multiplet). Several target compounds show spectra of at least two isomers in DMSO-*d*_6_ with a maximal ratio of 1:3. These are due to atropisomers and tautomers as already reported for similar 4,5-diaryl-imidazoles [32]. Although such effects have not been observed whenever CDCl_3_ was used, DMSO-*d*_6_ was the most frequented solvent with respect to its favorable solubility-mediating properties. For reasons of clarity, signals are only given for the main isomer. The labelling scheme of structures to correlate NMR signals can be found in Supporting Information. LC-MS was performed with an 1100 HPLC system (Agilent Technologies, Santa Clara, CA, USA) over an Agilent Eclipse XDB-C8 column (150 × 4.6 mm, 5 µm) using a 0.1% acetic acid/acetonitrile gradient for mobile phase (flow rate = 1 mL∙min^−1^). Mass spectra with nominal solution were recorded on a Bruker Esquire ~LC ion trap mass spectrometer with electron spray ionization (ESI) operating in the positive ion mode, with the following parameters: drying gas nitrogen 8 L∙min^−1^, nebulizer 35 psi, drying temperature 350 °C). HRMS spectra were recorded on an AccuTOF™ GCv 4G electron ionization (EI)/field desorption (FD) mass spectrometer (JEOL Germany, Freising, Germany). For clarity, only the highest measured peak is given for mass spectra. Melting points/decomposition temperatures were determined on a SMP3 Melting Point Apparatus (Stuart Scientific, Keison Products, Chelmsford, Essex, UK) and are uncorrected. Column chromatography was performed using a LaFlash system (VWR International GmbH, Darmstadt, Germany). The crude product was loaded on silica gel 60 (63–200 µm) (Macherey-Nagel, Düren, Germany) or PuriFlash IR-50 C18 modified silica gel (50 µm) (Interchim Deutschland GmbH, Mannheim, Germany) and packed in Interchim PuriFlash-DLE/12G dry-load precolumns. Pre-packed Interchim PuriFlash-30SIHP silica gel columns (30 µm, 40 g) and Interchim PuriFlash-15C18HP modified silica gel columns (15 µm, 55 g) were used for separation with flow rates usually adjusted to 30 mL∙min^−1^ or 20.5 mL∙min^−1^. Progress of reactions was monitored by thin-layer chromatography (TLC) performed with Macherey-Nagel 0.2 mm Polygram^®^ SIL G/UV_254_ pre-coated silica gel polyester sheets and Silicagel 60 RP-18 F_254_ modified silica gel aluminum plates (Merck Millipore, Darmstadt, Germany). Where necessary, reactions were carried out in a nitrogen atmosphere using 4 Å molecular sieves. All reagents and solvents were obtained from commercial sources (abcr GmbH, Karlsruhe, Germany; Sigma-Aldrich Chemie GmbH, Munich, Germany; Merck Group, Munich, Germany; Merck Millipore; Acros Organics Thermo Fisher Scientific, Geel, Belgium; VWR International GmbH, Hannover, Germany and used as received: THF was used after distillation over Na/benzophenone. HPLC analysis was performed on a Hewlett Packard HP 1050 Series using either a ZORBAX^®^ Eclipse XDB-C8 (150 mm × 4.6 mm, 5 µm) or a Kinetex^®^ C8 (150 × 4.6 mm, 5 µm) column (mobile phase flow 1.5 mL·min^−1^, gradient KH_2_PO_4_ buffer 10 mM, pH 2.3/methanol, UV-detection 254 nm). All key compounds submitted to biological assays were proven by this method to show ≥98% purity. Syntheses under elevated pressure were performed in Berghof high*preactor*™ BR-25 with corresponding heating block on a MR Hei-Standard laboratory heating plate (Heidolph, Schwabach, Germany). Microwave syntheses were performed in a CEM Discover Microwave Synthesizer (CEM GmbH, Kamp-Lintfort, Germany) under air cooling and high stirring with a maximal power of 100 W.

#### 3.2.1. Syntheses of Key Building Blocks **3**–**7**

*1-(4-Fluorophenyl)-2-(2-fluoropyridin-4-yl)-ethan-1-one* (**3**). NaHMDS (66.7 mL 2 M solution in THF, 133 mmol) was slowly added to a stirred solution of 2-fluoro-4-methylpyridine (10.6 mL, 103 mmol) and ethyl 4-fluorobenzoate (18.1 mL, 123 mmol) in 40 mL anhyd. THF at 0 °C under a nitrogen atmosphere. After stirring at 0 °C for 2 h the reaction was allowed to reach rt and stirring continued for 1 h. The mixture was diluted with ethyl acetate and washed twice with 10% aq. HCl. The organic layer was dried over anhyd. Na_2_SO_4_ and solvent was removed under reduced pressure. Recrystallization from ethyl acetate afforded **3** as colorless solid. Yield 23.9 g (quant.); C_13_H_9_F_2_NO (M*_r_* 233.22); m.p. 102 °C; ^1^H-NMR (DMSO-*d*_6_): δ = 4.59 (s, 2H, C*H*_2_), 7.10 (s, 1H, C_3_*H*, Pyr), 7.25–7.27 (m, 1H, C^5^*H*, Pyr), 7.37–7.43 (m, 2H, C^3/5^*H*, F-Phe), 8.11–8.19 (m, 3H, C^2/6^*H*, F-Phe, C^6^*H*, Pyr) ppm; ^13^C-NMR (DMSO-*d*_6_): δ = 43.6 (d, ^4^*J*_CF_ = 2.8 Hz, *C*H_2_), 110.8 (d, ^2^*J*_CF_ = 37.6 Hz, *C*^3^H, Pyr), 115.8 (d, ^2^*J*_CF_ = 22.0 Hz, *C*^3/5^H, F-Phe), 123.8 (d, ^4^*J*_CF_ = 3.8 Hz, *C*^5^H, Pyr), 131.3 (d, ^3^*J*_CF_ = 9.6 Hz, *C*^2/6^H, F-Phe), 132.9 (d, ^4^*J*_CF_ = 2.7 Hz, *C*^1^, F-Phe), 147.0 (d, ^5^*J*_CF_ = 15.5 Hz, *C*^6^H, Pyr), 150.7 (d, ^3^*J*_CF_ = 8.5 Hz, *C*^1^, Pyr), 163.1 (d, ^1^*J*_CF_ = 234.3 Hz, *C*^2^F, Pyr), 165.2 (d, ^1^*J*_CF_ = 252.0 Hz, *C*^4^F, F-Phe), 194.6 (*C*O) ppm; MS (ESI, 70 eV) *m*/*z* 234 [MH]^+^.

*1-(4-Fluorophenyl)-2-(2-fluoropyridin-4-yl)-2-(hydroximino)ethan-1-one* (**4**). NaNO_2_ (2.06 g, 29.8 mmol) in 12 mL H_2_O was slowly added to a stirred solution of **3** (2.30 g, 9.86 mmol) in 17 mL glacial acetic acid at 10 °C. After stirring at r.t. for 1 h, 30 mL H_2_O were added and stirring continued for 3.5 h. The suspension was cooled to 8 °C, filtered, and the residue was washed with H_2_O and dried under reduced pressure to afford **4** as colorless solid. Yield 2.46 g (95%); C_13_H_8_F_2_N_2_O_2_ (M*_r_* 262.22); m.p. 185 °C; ^1^H-NMR (DMSO-*d*_6_): δ = 7.19 (bs, 1H, C^3^*H*, Pyr), 7.37–7.46 (m, 3H, C^5^*H*, Pyr and C^3/5^*H*, F-Phe), 7.92–7.98 (m, 2H, C^2/6^*H*, F-Phe), 8.30 (d, ^3^*J* = 5.3 Hz, 1H, C^6^*H*, Pyr), 12.67 (s, 1H, O*H*) ppm; ^13^C-NMR (DMSO-*d*_6_): δ = 105.4 (d, ^2^*J*_CF_ = 38.9 Hz, *C*^3^H, Pyr), 116.7 (d, ^2^*J*_CF_ = 22.5 Hz, *C*^3/5^H, F-Phe), 118.2 (d, ^4^*J*_CF_ = 4.2 Hz, *C*^5^H, Pyr), 130.8 (d, ^4^*J*_CF_ = 2.7 Hz, *C*^1^, F-Phe), 132.2 (d, ^3^*J*_CF_ = 10.1 Hz, *C*^2/6^H, F-Phe), 144.3 (d, ^3^*J*_CF_ = 8.4 Hz, *C*^4^, Pyr), 148.8 (d, ^5^*J*_CF_ = 15.6 Hz, *C*^6^H, Pyr), 151.9 (d, ^4^*J*_CF_ = 4.0 Hz, *C*NOH), 163.5 (d, ^1^*J*_CF_ = 235.6 Hz, *C*^2^F, Pyr), 166.0 (d, ^1^*J*_CF_ = 254.8 Hz, *C*^4^F, F-Phe), 192.04 (*C*O) ppm; MS (ESI, 70 eV) *m*/*z* = 263 [MH]^+^.

*2-(4-Fluorophenyl)-1-(2-fluoropyridin-4-yl)-2-oxoethan-1-aminium chloride* (**5**). Pd/C 10% (279 mg) was added in one portion under a nitrogen atmosphere to an intensely stirred solution of **4** (1.60 g, 6.10 mmol) in 15 mL 2-propanol and 20 mL HCl sat. 2-propanol and stirring continued for 12 h at r.t. The crude product was obtained by filtration, the residue was resuspended in methanol, filtered again, and the solvent was removed under reduced pressure. Recrystallization from methanol/diethyl ether afforded **5** as beige solid. Yield 1.43 g (82%); C_13_H_11_ClF_2_N_2_O (M*_r_* 284.69); m.p. 216 °C; ^1^H-NMR (DMSO-*d*_6_): δ = 6.56 (s, 1H, C*H*), 7.35–7.41 (m, 2H, C^3/5^*H*, F-Phe), 7.52–7.54 (m, 2H, C^3/5^*H*, Pyr), 8.21 (dd, ^3^*J* = 8.4 Hz, ^4^*J* = 5.6 Hz, 2H, C^2/6^*H*, F-Phe), 8.31 (d, ^3^*J* = 5.4 Hz, 1H, C^6^*H*, Pyr), 9.36 (bs, 3H, N*H*_3_^+^) ppm; ^13^C-NMR (DMSO-*d*_6_): δ = 56.8 (*C*NH_3_^+^), 110.5 (d, ^2^*J*_CF_ = 39.3 Hz, *C*^3^H, Pyr), 116.9 (d, ^2^*J*_CF_ = 22.1 Hz, *C*^3/5^H, F-Phe), 122.3 (d, ^4^*J*_CF_ = 4.2 Hz, *C*^5^H, Pyr), 130.0 (d, ^4^*J*_CF_ = 2.7 Hz, *C*^1^, F-Phe), 132.9 (d, ^3^*J*_CF_ = 9.8 Hz, *C*^2/6^H, F-Phe), 147.4 (d, ^3^*J*_CF_ = 8.0 Hz, *C*^4^, Pyr), 149.4 (d, ^5^*J*_CF_ = 15.0 Hz, *C*^6^H, Pyr), 163.5 (d, ^1^*J*_CF_ = 236.7 Hz, *C*^2^F, Pyr), 166.3 (d, ^1^*J*_CF_ = 255.1 Hz, *C*^4^F, F-Phe), 191.4 (*C*O) ppm; MS (ESI, 70 eV) *m*/*z* 249 [MCl]^+^.

*2-Fluoro-4-(5-(4-fluorophenyl)-2-(methylthio)-1H-imidazol-4-yl)pyridine* (**6**). A mixture of **5** (4.41 g, 15.5 mmol) and methyl thiocyanate (3.18 mL, 46.5 mmol) was refluxed under a nitrogen atmosphere for 45 min in 140 mL anhyd. DMF and stirred 45 min at r.t. before ice-cold H_2_O (400 mL) was added. The suspension was cooled to 8 °C, filtered, the residue was washed with H_2_O, and dried under reduced pressure to afford **6** as fine yellow solid. Yield 3.59 g (76%); C_15_H_11_F_2_N_3_S (M*_r_* 303.33); m.p. 205 °C; ^1^H-NMR (DMSO-*d*_6_): δ = 2.63 (s, 3H, SC*H*_3_), 7.09 (bs, 1H, C^3^*H*, Pyr), 7.26–7.34 (m, 3H, C^5^*H*, Pyr and C^3/5^*H*, F-Phe), 7.49–7.54 (m, 2H, C^2/6^*H*, F-Phe), 8.09 (d, ^3^*J* = 5.3 Hz, 1H, C^6^*H*, Pyr), 12.84 (bs, 1H, N*H*) ppm; ^13^C-NMR (DMSO-*d*_6_): δ = 14.9 (S*C*H_3_), 105.0 (d, ^2^*J*_CF_ = 39.5 Hz, *C*^3^H, Pyr), 115.9 (d, ^2^*J*_CF_ = 21.6 Hz, *C*^3/5^H, F-Phe), 118.5 (d, ^4^*J*_CF_ = 2.5 Hz, *C*^5^H, Pyr), 126.5 (*C*^1^, F-Phe), 130.9 (*C*^2/6^H, F Phe), 132.9 (d, ^3^*J*_CF_ = 8.6 Hz, *C*^4^, Pyr), 143.0 (*C*^2^, Imdz), 147.6 (d, ^3^*J*_CF_ = 16.1 Hz, *C*^6^H, Pyr), 162.1 (d, ^1^*J*_CF_ = 246.4 Hz, *C*F, F-Phe), 163.7 (d, ^1^*J*_CF_ = 233.3 Hz, *C*F, Pyr) ppm; MS (ESI, 70 eV) *m*/*z* 304 [MH]^+^.

*4-(5-(4-Fluorophenyl)-2-(methylthio)-1H-imidazol-4-yl)pyridin-2-amine* (**7**). A solution of **6** (1.00 g, 3.30 mmol) in 15 mL 32% aq. ammonia solution was heated in a high pressure reactor at 180 °C for 18 h. The reactor was allowed to reach r.t. and H_2_O was added. The crude product was obtained by filtration, washed with H_2_O and diisopropyl ether and purified by flash chromatography (SiO_2_, 50%–100% ethyl acetate/petrol ether) to afford **7** as beige solid. Yield 852 mg (86%); C_15_H_13_FN_4_S (M*_r_* 300.36); ^1^H-NMR (DMSO-*d*_6_): δ = 2.60 (s, 1H, SC*H*_3_), 5.79–5.95 (m, 2H, N*H*_2_), 6.42–6.67 (m, 2H, C^3/5^*H*, Pyr), 7.17–7.27 (m, 2H, C^3/5^*H*, F-Phe), 7.48 (bs, 2H, C^2/6^*H*, F-Phe), 7.74–7.86 (m, 1H, C^6^*H*, Pyr), 12.58 (s, 1H, N*H*) ppm; ^13^C-NMR (DMSO-*d*_6_): δ = 15.1 (S*C*H_3_), 105.1 (*C*^5^H, Pyr), 110.0 (*C*^3^H, Pyr), 115.7 (d, ^3^*J*_CF_ = 21.1 Hz, *C*^3/5^H, F-Phe), 127.0 (s, *C*^4^/*C*^5^, Imdz), 129.2 (s, *C*^1^, F-Phe), 130.5 (d, ^4^*J*_CF_ = 6.6 Hz, *C*^2/6^H, F-Phe), 147.5 (*C*^6^H, Pyr), 160.1 (*C*F) ppm; MS (ESI, 70 eV) *m*/*z* 301 [MH]^+^.

#### 3.2.2. Synthesis of 4-(5-(4-Fluorophenyl)-2-(methylthio)-1*H*-imidazol-4-yl)pyridin-2-amines and -piperazines **8**, **9**, **10a**–**i**

*3-(2,4-Dimethoxyphenyl)propan-1-ol* (**8**). Under a nitrogen atmosphere a solution of LiAlH_4_ (120 mg, 3.16 mmol) in 3 mL anhyd. THF was slowly added to 3-(2,4-dimethoxyphenyl)propionic acid (508 mg, 2.42 mmol) in 6 mL anhyd. THF at r.t. with intense stirring that continued for 1 h. After completion, the reaction was cooled down in an ice-bath and quenched by successive addition of H_2_O (120 µL), 15% aq. NaOH solution (120 µL), and H_2_O (360 µL). The precipitate was filtered off, washed with THF, and the filtrate was concentrated under reduced pressure. The crude product was purified by flash chromatography (SiO_2_, 20%–30% ethyl acetate/petrol ether) to afford **8** as colorless oil. Yield 457 mg (96%); C_11_H_16_O_3_ (M*_r_* 196.25); ^1^H-NMR (CDCl_3_): δ = 1.78–1.83 (m, 2H, CH_2_C*H*_2_CH_2_OH), 2.65 (t, ^3^*J* = 7.3 Hz, 2H, C*H*_2_CH_2_CH_2_OH), 3.59 (t, ^3^*J* = 6.3 Hz, 1H, CH_2_CH_2_C*H*_2_OH), 3.78 (s, 1H, O*H*), 3.79 (s, 3H, C^4^OC*H*_3_), 3.81 (s, 3H, C^2^OC*H*_3_), 6.42–6.46 (m, 2H, C^3/5^*H*, (OCH_3_)_2_-Phe), 7.03 (dd, ^3^*J* = 7.7 Hz, ^5^*J* = 0.6 Hz, 1H, C^6^*H*, (OCH_3_)_2_-Phe) ppm; ^13^C-NMR (CDCl_3_): δ = 25.4 (*C*H_2_CH_2_CH_2_OH), 33.2 (CH_2_*C*H_2_CH_2_OH), 55.5 (C^2/4^O*C*H_3_), 62.1 (CH_2_CH_2_*C*H_2_OH), 98.6 (*C*^3^H, (OCH_3_)_2-_Phe), 104.3 (*C*^5^H, (OCH_3_)_2_-Phe), 122.4 (*C*^1^, (OCH_3_)_2_-Phe), 130.4 (*C*^6^H, (OCH_3_)_2_-Phe), 158.3 (*C*^2^OCH_3_), 159.3 (*C*^4^OCH_3_) ppm.

*1-(3-Bromopropyl)-2,4-dimethoxybenzene* (**9**). To an ice-cold solution of **8** (260 mg, 1.33 mmol) in 4 mL anhyd. DCM, triphenylphosphine (412 mg, 1.57 mmol) and *N*-bromosuccinimide (263 mg, 1.48 mmol) were added under a nitrogen atmosphere and the reaction was stirred for 2 h. The mixture was washed with H_2_O and sat. aq. NaCl solution, dried over anhyd. Na_2_SO_4_, and the solvent was removed under reduced pressure. Diethyl ether was added, the precipitate filtered off, and the filtrate was concentrated and purified by flash chromatography (SiO_2_, 2%–10% ethyl acetate/petrol ether) to afford **9** as colorless oil. Yield 205 mg (60%); C_11_H_15_BrO (M*_r_* 259.14); ^1^H-NMR (CDCl_3_): δ = 2.11 (quint, ^3^*J* = 5.6 Hz, 2H, CH_2_C*H*_2_CH_2_Br), 2.70 (t, ^3^*J* = 7.2 Hz, 2H, C*H*_2_CH_2_CH_2_Br), 3.39 (t, ^3^*J* = 6.8 Hz, 2H, CH_2_CH_2_C*H*_2_Br), 3.80 (s, 6H, C^2/4^OC*H*_3_), 6.42 (dd, ^3^*J* = 8.0 Hz, ^4^*J* = 2.5 Hz, 1H, C^5^*H*, (OCH_3_)_2_-Phe), 6.45 (d, ^4^*J* = 2.3 Hz, 1H, C^3^*H*, (OCH_3_)_2_-Phe), 7.05 (dd, ^3^*J* = 8.0 Hz, ^5^*J* = 0.5 Hz, 1H, C^6^*H*, (OCH_3_)_2_-Phe) ppm; ^13^C-NMR (CDCl_3_): δ = 28.4 (CH_2_*C*H_2_CH_2_Br), 33.0 (*C*H_2_CH_2_CH_2_Br), 33.9 (CH_2_CH_2_*C*H_2_Br), 55.3 (C^4^O*C*H_3_), 55.5 (C^2^O*C*H_3_), 98.7 (*C*^3^H, (OCH_3_)_2_-Phe), 103.9 (*C*^5^H, (OCH_3_)_2_-Phe), 121.4 (*C*^1^, (OCH_3_)_2_-Phe), 130.5 (*C*^6^H, (OCH_3_)_2_-Phe), 158.5 (*C*^2^OCH_3_), 159.6 (*C*^4^OCH_3_) ppm.

*N-(3-(2,4-Dimethoxyphenyl)propyl)-4-(5-(4-fluorophenyl)-2-(methylthio)-1H-imidazol-4-yl)pyridin-2-amine* (**10a**). The synthetic protocol starting from Boc-protected 2-amino-4-methylpyridine and **9** predominantly matches the procedure described for **7** and can be found in the Supporting Information. ^1^H-NMR (DMSO-*d*_6_): δ = 1.67–1.72 (m, 2H, CH_2_C*H*_2_CH_2_NH), 2.48–2.52 (m, 2H, C*H*_2_CH_2_CH_2_NH), 2.60 (s, 3H, SC*H*_3_), 3.13–3.17 (m, 2H, CH_2_CH_2_C*H*_2_NH), 3.72 (s, 3H, C^4^OC*H*_3_), 3.74 (s, 3H, C^2^OC*H*_3_), 6.41–6.50 (m, 5H, C^3/5^*H*, Pyr and C^3/5^*H*, (OCH_3_)_2_-Phe and CH_2_CH_2_CH_2_N*H*), 7.01 (d, ^3^*J* = 8.2 Hz, 1H, C^6^*H*, (OCH_3_)_2_-Phe), 7.22 (m, 2H, C^3/5^*H*, F-Phe), 7.48 (m, 2H, C^2/6^*H*, F-Phe), 7.85 (bs, C^6^*H*, Pyr), 12.58 (bs, 1H, N*H*) ppm; ^13^C-NMR (DMSO-*d*_6_): δ = 15.1 (S*C*H_3_), 26.6 (*C*H_2_CH_2_CH_2_NH), 29.3 (CH_2_*C*H_2_CH_2_NH), 40.7 (CH_2_CH_2_*C*H_2_NH), 55.1 (C^4^O*C*H_3_), 55.2 (C^2^O*C*H_3_), 98.3 (*C*^3^H, (OCH_3_)_2_-Phe), 104.3 (*C*^5^H, (OCH_3_)_2_-Phe), 104.9 (*C*^3^H, Pyr), 109.7 (*C*^5^H, Pyr), 115.4 (*C*^3/5^H, F-Phe), 121.8 (*C*^1^, (OCH_3_)_2_-Phe), 126.5 (*C*^4^, Imdz), 127.0 (*C*^5^, Imdz), 129.7 (*C*^1^, F-Phe), 129.7 (*C*^6^H, (OCH_3_)_2_-Phe), 130.1 (*C*^2/6^H, F-Phe), 142.1 (*C*^2^, Imdz), 147.5 (*C*^6^H, Pyr), 157.9 (*C*^2^OCH_3_), 158.8 (*C*^4^OCH_3_), 159.3 (*C*^2^, Pyr) ppm; MS (ESI, 70 eV) *m*/*z* 479 [MH]^+^; HRMS (EI, 70 eV) *m*/*z* [M]^+^ calcd. for C_26_H_27_FN_4_O_2_S, 478.1839; found, 478.1839.

##### General Procedure for the Preparation Compounds **10b**–**i**

Compound **6** (1.0 equiv) was suspended in the appropriate amine/piperazine (4.0 to 6.0 equiv) and the intensely stirred mixture was heated to 160 °C. Progress of the reaction was monitored by HPLC control. After complete conversion the mixture was diluted with ethyl acetate, washed with sat. aq. NaHCO_3_ solution and H_2_O, dried over anhyd. Na_2_SO_4_, and concentrated under reduced pressure. The crude product was purified by flash chromatography (stationary phase, eluent, and mixing ratio given for each compound, respectively) to afford the particular compound.

*N-(2,4-Dimethoxyphenyl)-4-(5-(4-fluorophenyl)-2-(methylthio)-1H-imidazol-4-yl)-pyridin-2-amine* (**10b**). Synthesis was performed according to the general procedure from **6** (400 mg, 1.32 mmol) and 2,4-dimethoxyaniline (808 mg, 5.27 mmol). Purification was achieved by flash chromatography (SiO_2_, 20%–90% ethyl acetate/petrol ether) to afford **10b** as greyish crystals. Yield 312 mg (54%); C_23_H_21_FN_4_O_2_S (M*_r_* 436.51); m.p. 206 °C; ^1^H-NMR (DMSO-*d*_6_): δ = 2.60 (s, 3H, SC*H*_3_), 3.75 (s, 6H, 2 OC*H*_3_), 6.39–6.44 (m, 1H, C^5^*H*, (OCH_3_)_2_-Phe), 6.56–6.58 (m, 1H, C^3^*H*, (OCH_3_)_2_-Phe), 6.63 (dd, ^3^*J =* 5.3 Hz, ^4^*J =* 1.2 Hz, 1H, C^5^*H*, Pyr), 6.82 (s, 1H, C^3^*H*, Pyr), 7.18–7.28 (m, 2H, C^3/5^*H*, F-Phe), 7.41–7.46 (m, 2H, C^2/6^*H*, F-Phe), 7.52 (d, ^3^*J =* 8.7 Hz, 1H, C^6^*H*, (OCH_3_)_2_-Phe), 7.71 (s, 1H, N*H*), 7.95 (d, ^3^*J =* 5.3 Hz, 1H, C^6^*H*, Pyr), 12.60 (s, 1H, N*H*, Imdz) ppm; ^13^C-NMR (DMSO-*d*_6_): δ = 15.1 (S*C*H_3_), 55.2 (O*C*H_3_), 55.2 (O*C*H_3_), 99.0 (*C*^3^H, (OCH_3_)_2_-Phe), 104.0 (*C*^5^H, (OCH_3_)_2_-Phe), 105.4 (*C*^3^H, Pyr), 111.5 (*C*^5^H, Pyr), 115.4 (dd, ^2^*J*_CF_ = 21.8 Hz, *C*^3/5^H, F-Phe), 122.5 (*C*, Imdz), 123.6 (*C*^6^H, (OCH_3_)_2_-Phe), 126.8 (d, ^4^*J*_CF_ = 3.1 Hz, *C*^1^, F-Phe), 130.0 (d, ^3^*J*_CF_ = 8.1 Hz, *C*^2/6^H, F-Phe), 134.9 (*C*^1^, (OCH_3_)_2_-Phe), 138.8 (*C*, Imdz), 141.7 (*C*^2^OCH_3_), 142.7 (*C*^2^, Imdz), 147.6 (*C*^6^H, Pyr), 152.2 (*C*^4^OCH_3_), 155.9 (*C*^4^, Pyr), 157.4 (*C*^2^, Pyr), 161.8 (d, ^1^*J*_CF_ = 245.0 Hz, *C*F) ppm; MS (ESI, 70 eV) *m*/*z* 437 [MH]^+^.

*N-(2-Ethoxyphenyl)-4-(5-(4-fluorophenyl)-2-(methylthio)-1H-imidazol-4-yl)pyridin-2-amine* (**10c**). Synthesis was performed according to the general procedure from **6** (1.50 g, 4.95 mmol) and 2-ethoxyaniline (3.88 mL, 29.7 mmol). Purification was achieved by flash chromatography (SiO_2_, 20%–90% ethyl acetate/petrol ether) to afford **10c** as dark red crystals. Yield 1.10 g (52%); C_23_H_21_FN_4_OS (M*_r_* 420.51); m.p. 179 °C; ^1^H-NMR (CDCl_3_): δ = 1.39 (t, ^3^*J =* 7.0 Hz, 3H, CH_2_C*H*_3_), 2.63 (s, 3H, SC*H*_3_), 4.03 (q, ^3^*J =* 7.0 Hz, 2H, C*H*_2_CH_3_), 6.76 (t, ^3^*J =* 7.7 Hz, 1H, C^5^*H*, EtO-Phe), 6.81–6.84 (m, 2H, C^5^*H*, Pyr and C^3^*H*, EtO-Phe), 6.91 (t, ^3^*J =* 7.7 Hz, 1H, C^4^*H*, EtO-Phe), 6.97 (bs, 1H, C^3^*H*, Pyr), 7.00–7.05 (t, ^3^*J =* 8.7 Hz, 2H, C^3/5^*H*, F-Phe), 7.21 (bs, 1H, N*H*), 7.37–7.41 (m, 2H, C^2/6^*H*, F-Phe), 7.49 (d, ^3^*J =* 7.7 Hz, 1H, C^6^*H*, EtO-Phe), 8.00 (d, ^3^*J =* 5.5 Hz, 1H, C^6^*H*, Pyr) ppm; ^13^C-NMR (CDCl_3_): δ = 15.0 (*C*H_3_CH_2_), 16.6 (S*C*H_3_), 64.3 (*C*H_2_CH_3_), 106.5 (*C*^3^H, Pyr), 111.7 (*C*^3^H, EtO-Phe), 112.9 (*C*^5^H, Pyr), 116.0 (d, ^2^*J*_CF_ = 21.7 Hz, *C*^3/5^H, F-Phe), 120.0 (*C*^6^H, EtO-Phe), 120.7 (*C*^5^H, EtO-Phe), 122.6 (*C*^4^H, EtO-Phe), 129.4 (*C*^1^, EtO-Phe), 130.5 (d, ^3^*J*_CF_ = 8.1 Hz, *C*^2/6^H, F-Phe), 143.2 (*C*^2^, Imdz), 146.8 (*C*^6^H, Pyr), 148.7 (*C*OEt), 155.5 (*C*^2^, Pyr), 162.5 (d, ^1^*J*_CF_ = 249.0 Hz, *C*F) ppm; MS (ESI, 70 eV) *m*/*z* = 421 [MH]^+^.

*N-(3,4-Dimethoxyphenethyl)-4-(5-(4-fluorophenyl)-2-(methylthio)-1H-imidazol-4-yl)-pyridin-2-amine* (**10d**). Synthesis was performed according to the general procedure from **6** (400 mg, 1.32 mmol) and 3,4-dimethoxyphenethylamine (900 µL, 5.31 mmol). Purification was achieved by flash chromatography (SiO_2_, 20%–90% ethyl acetate/petrol ether) to afford **10d** as yellow crystals. Yield 550 mg (90%); C_25_H_25_FN_4_O_2_S (M*_r_* 464.56); m.p. 87 °C; ^1^H-NMR (CDCl_3_): δ = 2.55 (s, 3H, SC*H*_3_), 2.69 (t, ^3^*J =* 6.9 Hz, 2H, C*H*_2_CH_2_NH), 3.29 (m, 2H, CH_2_C*H*_2_NH), 3.74 (s, 3H, C^3^OC*H*_3_), 3.75 (s, 3H, C^4^OC*H*_3_), 4.86 (bs, 1H, CH_2_CH_2_N*H*), 6.51 (d, ^3^*J =* 5.2 Hz, 1H, C^5^*H*, Pyr), 6.57 (d, ^4^*J* = 2.0 Hz, 1H, C^3^*H*, Pyr), 6.57–6.70 (m, 3H, C^2/5/6^*H*, (OCH_3_)_2_-Phe), 6.91–6.96 (m, 2H, C^3/5^*H*, F-Phe), 7.32–7.36 (m, 2H, C^2/6^*H*, F-Phe), 7.74 (d, ^3^*J =* 5.8 Hz, 1H, C^6^*H*, Pyr), 10.77 (bs, 1H, N*H*) ppm; ^13^C-NMR (CDCl_3_): δ = 16.4 (S*C*H_3_), 35.1 (*C*H_2_CH_2_NH), 43.5 (CH_2_*C*H_2_NH), 55.9 (C^3^O*C*H_3_), 55.9 (C^4^O*C*H_3_), 104.1 (*C*^3^H, Pyr), 111.0 (*C*^5^H, Pyr), 111.4 (*C*^5^H, (OCH_3_)_2_-Phe), 112.1 (*C*^2^H, (OCH_3_)_2_-Phe), 115.7 (d, ^2^*J*_CF_ = 21.7 Hz, *C*^3/5^H, F-Phe), 120.7 (*C*^6^H, (OCH_3_)_2_-Phe), 130.3 (d, ^3^*J*_CF_ = 8.2 Hz, *C*^2/6^H, F-Phe), 131.4 (*C*^4/5^, Imdz), 142.9 (*C*^4^, Pyr), 146.7 (*C*^6^H, Pyr, 147.7 (*C*^4^OCH_3_), 149.0 (*C*^3^OCH_3_), 158.3 (*C*^2^, Pyr), 162.6 (d, ^1^*J*_CF_ = 248.5 Hz, *C*F) ppm; MS (ESI, 70 eV) *m*/*z* 465 [MH]^+^.

*N-(2-(1H-Indol-3-yl)-ethyl)-4-(5-(4-fluorophenyl)-2-(methylthio)-1H-imidazol-4-yl)-pyridin-2-amine* (**10e**). Synthesis was performed according to the general procedure from **6** (400 mg, 1.32 mmol) and tryptamine (845 mg, 5.27 mmol). Purification was achieved by flash chromatography (SiO_2_, 20%–90% ethyl acetate/petrol ether) to afford **10e** as yellowish-brown crystals. Yield 427 mg (73%); C_25_H_22_FN_5_S (M*_r_* 443.54); m.p. 122 °C; ^1^H-NMR (CDCl_3_): δ = 2.61 (s, 3H, SC*H*_3_), 2.95 (t, ^3^*J =* 6.6 Hz, 2H, C*H*_2_CH_2_NH), 3.39 (t, ^3^*J =* 6.6 Hz, 2H, CH_2_C*H*_2_NH), 6.52 (d, ^3^*J =* 5.7 Hz, 1H, C^5^*H*, Pyr), 6.52 (bs, 1H, C^2^*H*, Indole), 6.87 (d, ^4^*J* = 2.1 Hz, 1H, C^3^*H*, Pyr), 6.95–7.01 (m, 2H, C^3/5^*H*, F-Phe), 7.08 (t, ^3^*J =* 7.8 Hz, 1H, C^6^*H*, Indole), 7.16 (t, ^3^*J =* 7.9 Hz, 1H, C^5^*H*, Indole), 7.30 (d, ^3^*J =* 7.9 Hz, 1H, C^4^*H*, Indole), 7.35–7.39 (m, 2H, C^2/6^*H*, F-Phe), 7.52 (d, ^3^*J =* 7.7 Hz, 1H, C^7^*H*, Indole), 7.71 (d, ^3^*J =* 5.7 Hz, 1H, C^6^*H*, Pyr), 8.38 (s, 1H, N*H*, Indole) ppm; ^13^C-NMR (CDCl_3_): δ = 16.4 (S*C*H_3_), 25.1 (*C*H_2_CH_2_NH), 42.2 (CH_2_*C*H_2_NH), 104.0 (*C*^3^H, Pyr), 110.6 (*C*^5^H, Pyr), 111.3 (*C*^4^H, Indole), 112.6 (*C*^3^, Indole), 115.8 (d, ^2^*J*_CF_ = 21.6 Hz, C^3/5^*H*, F-Phe), 118.6 (*C*^7^H, Indole), 119.4 (*C*^6^H, Indole), 122.1 (*C*^5^H, Indole), 122.5 (*C*^2^H, Indole), 127.2 (*C*^8a^, Indole), 130.3 (^3^*J*_CF_ = 8.3 Hz, *C*^2/6^H, F-Phe), 136.4 (*C*^3a^, Indole), 143.3 (*C*^2^, Imdz), 145.1 (*C*^6^H, Pyr), 158.0 (*C*^2^, Pyr) ppm; MS (ESI, 70 eV) *m*/*z* 444 [MH]^+^.

*N-(4-(5-(4-Fluorophenyl)-2-(methylthio)-1H-imidazol-4-yl)pyridin-2-yl)-N′,N′-dimethyl-propan-1,3-diamine* (**10f**). Synthesis was performed according to the general procedure from **6** (400 mg, 1.32 mmol) and *N*^1^,*N*^1^-dimethylpropane-1,3-diamine (1.00 mL, 7.93 mmol). Purification was achieved by flash chromatography (SiO_2_, 20%–90% ethyl acetate/petrol ether, then methanol) and subsequent filtration, to afford **10f** as yellow solid. Yield 175 mg (35%); C_20_H_24_FN_5_S (M*_r_* 385.51); m.p. 129 °C; ^1^H-NMR (CDCl_3_): δ = 1.61 (t, ^3^*J =* 6.8 Hz, 2H, CH_2_C*H*_2_CH_2_), 2.13 (s, 6H, N(C*H*_3_)_2_), 2.29 (t, ^3^*J =* 6.9 Hz, 2H, C*H*_2_N(CH_3_)_2_), 2.58 (s, 3H, SC*H*_3_), 3.11 (t, ^3^*J =* 6.7 Hz, 2H, C*H*_2_NH), 5.22 (bs, 1H, CH_2_N*H*), 6.42–6.46 (m, 2H, C^3/5^*H*, Pyr), 6.95–7.01 (m, 2H, C^3/5^*H*, F-Phe), 7.35–7.39 (m, 2H, C^2/6^*H*, F-Phe), 7.78 (dd, ^3^*J =* 5.4 Hz, ^5^*J =* 0.6 Hz, 1H, C^6^*H*, Pyr) ppm; ^13^C-NMR (CDCl_3_): δ = 16.5 (S*C*H_3_), 26.7 (CH_2_*C*H_2_CH_2_), 45.2 (N(*C*H_3_)_2_), 57.6 (*C*H_2_N(CH_3_)_2_), 104.0 (*C*^3^H, Pyr), 111.1 (*C*^5^H, Pyr), 115.5 (d, ^2^*J*_CF_ = 21.5 Hz, *C*^3/5^H, F-Phe), 130.2 (d, ^3^*J*_CF_ = 8.1 Hz, *C*^2/6^H, F-Phe), 142.7 (*C*^2^, Imdz), 147.9 (*C*^6^H, Pyr), 159.2 (*C*^2^, Pyr), 162.4 (d, ^1^*J*_CF_ = 247.7 Hz, *C*F) ppm; MS (ESI, 70 eV) *m*/*z* 386 [MH]^+^.

*1-(4-(5-(4-Fluorophenyl)-2-(methylthio)-1H-imidazol-4-yl)pyridin-2-yl)-piperazine* (**10g**). Synthesis was performed according to the general procedure from **6** (300 mg, 989 µmol) and piperazine (341 mg, 3.96 mmol). Purification was achieved by flash chromatography (bas. Al_2_O_3_, 100% methanol) to afford **10g** as yellow solid. Yield 63.0 mg (17%); C_19_H_20_FN_5_S (M*_r_* 369.46); m.p. 146 °C; ^1^H-NMR (DMSO-*d*_6_): δ = 2.60 (bs, 3H, SC*H*_3_), 2.73 (t, ^3^*J =* 4.5 Hz, 4H, C^2/6^*H*_2_, Piperazine), 3.31 (t, ^3^*J =* 4.6 Hz, 4H, C^3/5^*H*_2_, Piperazine), 6.56 (d, ^3^*J =* 5.1 Hz, 1H, C^5^*H*, Pyr), 6.80 (bs, 1H, C^3^*H*, Pyr), 7.20–7.26 (m, 2H, C^3/5^*H*, F-Phe), 7.47–7.51 (m, 2H, C^2/6^*H*, F-Phe), 7.97 (d, ^3^*J =* 5.1 Hz, 1H, C^6^*H*, Pyr) ppm; ^13^C-NMR (DMSO-*d*_6_): δ = 15.1 (S*C*H_3_), 45.3 (*C*^2/6^H_2_, Piperazine), 45.8 (*C*^3/5^H_2_, Piperazine), 103.8 (*C*^3^H, Pyr), 110.6 (*C*^5^H, Pyr), 115.4 (d, ^2^*J*_CF_ = 21.6 Hz, *C*^3/5^H, F-Phe), 130.3 (d, ^3^*J*_CF_ = 7.6 Hz, *C*^2/6^H, F-Phe), 142.3 (*C*^2^, Imdz), 147.6 (*C*^6^H, Pyr), 159.6 (*C*^2^, Pyr), 161.6 (d, ^1^*J*_CF_ = 245.1 Hz, *C*F) ppm; MS (ESI, 70 eV) *m*/*z* 370 [MH]^+^.

*1-(4-(5-(4-Fluorophenyl)-2-(methylthio)-1H-imidazol-4-yl)pyridin-2-yl)-4-methyl-piperazine* (**10h**). Synthesis was performed according to the general procedure from **6** (400 mg, 1.32 mmol) and *N*-methylpiperazine (600 µL, 5.39 mmol). Purification was achieved by flash chromatography (RP-18, 20%–90% methanol/H_2_O) to afford **10h** as pale yellow solid. Yield 115 mg (23%); C_20_H_22_FN_5_S (M*_r_* 383.49); m.p. 111 °C; ^1^H-NMR (CDCl_3_): δ = 2.28 (s, 3H, C*H*_3_), 2.45 (t, ^3^*J =* 4.8 Hz, 4H, C^3/5^*H*_2_, Piperazine), 2.57 (s, 3H, SC*H*_3_), 3.40 (t, ^3^*J =* 4.8 Hz, 4H, C^2/6^*H*_2_, Piperazine), 6.54 (dd, ^3^*J =* 5.3 Hz, ^4^*J =* 1.1 Hz, 1H, C^5^*H*, Pyr), 6.73 (bs, 1H, C^3^*H*, Pyr), 6.91–6.97 (m, 2H, C^3/5^*H*, F-Phe), 7.32–7.37 (m, 2H, C^2/6^*H*, F-Phe), 7.94 (d, ^3^*J =* 5.3 Hz, 1H, C^6^*H*, Pyr) ppm; ^13^C-NMR (CDCl_3_): δ = 16.5 (S*C*H_3_), 44.7 (*C*^2/6^H_2_, Piperazine), 45.6 (*C*H_3_), 54.4 (*C*^3/5^H_2_, Piperazine), 104.9 (*C*^3^H, Pyr), 111.9 (*C*^5^H, Pyr), 115.7 (d, ^2^*J*_CF_ = 21.6 Hz, *C*^3/5^H, F-Phe), 128.2 (d, ^4^*J*_CF_ *=* 4.1 Hz, *C*^C1^, F-Phe), 130.2 (d, ^3^*J*_CF_ = 8.1 Hz, *C*^2/6^H, F-Phe), 142.8 (*C*^2^, Imdz), 147.9 (*C*^6^H, Pyr), 159.6 (*C*^2^, Pyr), 162.5 (d, ^1^*J*_CF_ = 248.3 Hz, *C*F) ppm; MS (ESI, 70 eV) *m*/*z* 384 [MH]^+^.

*1-(4-(5-(4-Fluorophenyl)-2-(methylthio)-1H-imidazol-4-yl)pyridin-2-yl)-4-phenyl-piperazine* (**10i**). Synthesis was performed according to the general procedure from **6** (400 mg, 1.32 mmol) and *N*-phenylpiperazine (800 µL, 5.23 mmol). The combined organic phases were extracted with 2 M aq. HCl solution, the aq. layer was neutralized with 2 M aq. KOH solution, and the pale yellow precipitate was collected by filtration, and purified by flash chromatography (SiO_2_, 20%–90% ethyl acetate/petrol ether) to afford **10i** as yellow crystals. Yield 291 mg (50%); C_20_H_22_FN_5_S (M*_r_* 445.56); m.p. 101 °C; ^1^H- NMR (CDCl_3_): δ = 2.64 (s, 3H, SC*H*_3_), 3.19 (t, ^3^*J =* 5.1 Hz, 4H, C^2/6^*H*_2_, Piperazine), 3.58 (t, ^3^*J =* 5.1 Hz, 4H, C^3/5^*H*_2_, Piperazine), 6.64 (dd, ^3^*J =* 5.4 Hz, ^4^*J =* 1.2 Hz, 1H, C^5^*H*, Pyr), 6.93–6.85 (m, 4H, C^3^*H*, Pyr and C^2/4/6^*H*, Phe), 6.99–7.05 (m, 2H, C^3/5^*H*, F-Phe), 7.26 (t, ^3^*J =* 8.0 Hz, 2H, C^3/5^*H*, Phe), 7.39–7.44 (m, 2H, C^2/6^*H*, F-Phe), 8.00 (d, ^3^*J =* 5.5 Hz, 1H, C^6^*H*, Pyr) ppm; ^13^C-NMR (CDCl_3_): δ = 16.5 (S*C*H_3_), 45.4 (*C*^2/6^H_2_, Piperazine), 49.0 (*C*^3/5^H_2_, Piperazine), 105.1 (*C*^3^H, Pyr), 111.6 (*C*^5^H, Pyr), 115.8 (d, ^2^*J*_CF_ = 21.7 Hz, *C*^3/5^H, F-Phe), 116.3 (*C*^2/6^H, Phe), 120.2 (*C*^4^H, Phe), 129.2 (*C*^3/5^H, Phe), 130.3 (d, ^3^*J*_CF_ = 8.1 Hz, *C*^2/6^H, F-Phe), 143.0 (*C*^2^, Imdz), 146.9 (*C*^6^H, Pyr), 151.1 (*C*^1^, Phe), 159.0 (*C*^2^, Pyr), 162.6 (d, ^1^*J*_CF_ = 248.5 Hz, *C*F) ppm; MS (ESI, 70 eV) *m*/*z* 446 [MH]^+^.

#### 3.2.3. Synthesis of *N*-(4-(5-(4-Fluorophenyl)-2-(methylthio)-1*H*-imidazol-4-yl)pyridin-2-yl)-3-phenyl-propanamides **11a**–**e**

The appropriate methoxy-substituted 3-phenylpropionic acid and CDI (1.1 equiv) were stirred in 3–4 mL anhyd. DMF until formation of CO_2_ was undetectable. **7** was added to the reaction and the mixture was heated at 110 °C under a nitrogen atmosphere for 12 h. The reaction was cooled to rt and ethyl acetate was added. The resulting mixture was washed with H_2_O and sat. aq. NaCl solution, dried over anhyd. Na_2_SO_4_, and the solvent was removed under reduced pressure. The crude product was purified by flash chromatography (SiO_2_ and RP-18, eluent and mixing ratio given for each compound, respectively) to afford the particular compound.

*3-(2,4-Dimethoxyphenyl)-N-(4-(5-(4-fluorophenyl)-2-(methylthio)-1H-imidazol-4-yl)-pyridin-2-yl)propanamide* (**11a**). Synthesis was performed according to the general procedure from 3-(2,4-dimethoxyphenyl)propionic acid (560 mg, 2.66 mmol) and **7** (400 mg, 1.33 mmol). Purification was achieved by flash chromatography (SiO_2_, 30%–50% ethyl acetate/petrol ether and RP-18, 30%–80% methanol/H_2_O) to afford **11a** as beige solid. Yield 177 mg (27%); C_26_H_25_FN_4_O_3_S (M*_r_* 492.57); m.p. 90 °C; ^1^H-NMR (DMSO-*d*_6_): δ = 2.57 (t, ^3^*J* = 7.4 Hz, 2H, COC*H*_2_CH_2_), 2.62 (s, 3H, SC*H*_3_), 2.75 (t, ^3^*J* = 7.5 Hz, 2H, COCH_2_C*H*_2_), 3.72 (s, 3H, C^2^O*H*_3_), 3.77 (s, 3H, C^4^OC*H*_3_), 6.42 (dd, ^3^*J* = 8.3 Hz, ^4^*J* = 2.4 Hz, 1H, C^5^*H*, (OCH_3_)_2_-Phe), 6.51 (d, ^4^*J* = 2.4 Hz, 1H, C^3^*H*, (OCH_3_)_2_-Phe), 6.99 (dd, ^3^*J* = 5.3 Hz, 1H, C^5^*H*, Pyr), 7.03 (d, ^3^*J* = 8.3 Hz, 1H, C^6^*H*, (OCH_3_)_2_-Phe), 7.28 (bs, 2H, C^3/5^*H*, F-Phe), 7.46–7.51 (m, 2H, C^2/6^*H*, F-Phe), 8.12 (bs, 1H, C^6^*H*, Pyr), 8.32 (bs, 1H, C^3^*H*, Pyr), 10.29 (bs, 1H, CON*H*), 12.72 (bs, 1H, N*H*) ppm; ^13^C-NMR (DMSO-*d*_6_): δ = 15.1 (S*C*H_3_), 24.7 (COCH_2_*C*H_2_), 36.3 (CO*C*H_2_CH_2_), 55.1 (C^2^O*C*H_3_), 55.3 (C^4^O*C*H_3_), 98.3 (*C*^3^H, (OCH_3_)_2_-Phe), 104.3 (*C*^5^H, (OCH_3_)_2_-Phe), 110.6 (*C*^3^H, Pyr), 115.8 (d, ^2^*J*_CF_ = 24.1 Hz, *C*^3/5^H, F-Phe), 116.4 (*C*^5^H, Pyr), 120.9 (*C*^1^, (OCH_3_)_2_-Phe), 126.7 (*C*^1^, F-Phe), 129.8 (*C*^6^H, (OCH_3_)_2_-Phe), 130.7 (*C*^5^, Imdz and *C*^2/6^H, F-Phe), 134.5 (*C*^4^, Imdz), 143.7 (*C*^2^, Imdz), 147.6 (*C*^6^H, Pyr), 148.0 (*C*^4^, Pyr), 152.5 (*C*^2^, Pyr), 157.9 (*C*^4^, (OCH_3_)_2_-Phe), 159.0 (*C*^2^, (OCH_3_)_2_-Phe), 161.9 (d, ^1^*J*_CF_ = 246.7 Hz, *C*F), 171.4 (*C*O) ppm; IR (ATR): ν = 2935, 1670, 1609, 1547, 1505, 1414, 1289, 1262, 1221, 1207, 1153, 1121, 1036, 835 cm^−1^; MS (ESI, 70 eV) *m*/*z* 493 [MH]^+^; HRMS (EI, 70 eV) *m*/*z* [M]^+^ calcd. for C_26_H_25_FN_4_O_3_S, 492.1631; found, 492.1631.

**Figure 6:**
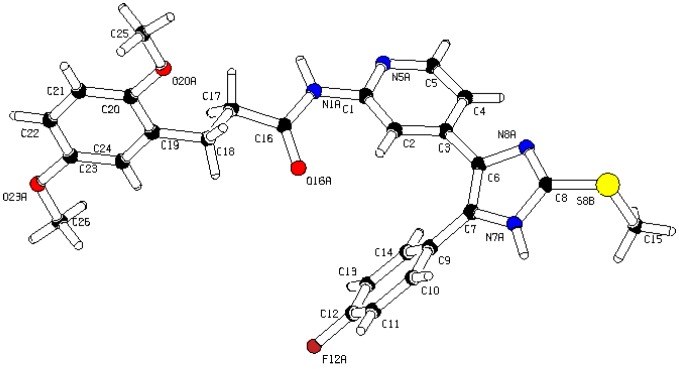
Small molecule X-ray crystal structure determination of compound **11b**.

*3-(2,5-Dimethoxyphenyl)-N-(4-(5-(4-fluorophenyl)-2-(methylthio)-1H-imidazol-4-yl)-pyridin-2-yl)-propanamide* (**11b**). Synthesis was performed according to the general procedure from 3-(2,5-dimethoxyphenyl)propionic acid (560 mg, 2.66 mmol) and **7** (400 mg, 1.33 mmol). Purification was achieved by flash chromatography (SiO_2_, 20%–100% ethyl acetate/petrol ether and RP-18, 20%–100% methanol/H_2_O) to afford **11b** as colorless solid. Yield 255 mg (39%); C_26_H_25_FN_4_O_3_S (M*_r_* 492.57); m.p. 91 °C; ^1^H-NMR (DMSO-*d*_6_): δ = 2.59–2.64 (m, 5H, SC*H*_3_ and COC*H*_2_CH_2_), 2.80 (t, ^3^*J* = 7.5 Hz, 2H, COCH_2_C*H*_2_), 3.66 (s, 3H, C^5^OC*H*_3_), 3.73 (s, 3H, C^2^OC*H*_3_), 6.72 (dd, ^3^*J* = 8.8 Hz, ^4^*J* = 3.1 Hz, 1H, C^4^*H*, (OCH_3_)_2_-Phe), 6.78 (d, ^4^*J* = 3.0 Hz, 1H, C^6^*H*, (OCH_3_)_2_-Phe), 6.86 (d, ^3^*J* = 8.8 Hz, 1H, C^3^*H*, (OCH_3_)_2_-Phe), 6.99 (dd, ^3^*J* = 5.2 Hz, ^4^*J* = 1.6 Hz, 1H, C^5^*H*, Pyr), 7.26 (bs, 2H, C^3/5^*H*, F-Phe), 7.46–7.51 (m, 2H, C^2/6^*H*, F-Phe), 8.13 (bs, 1H, C^6^*H*, Pyr), 8.32 (bs, 1H, C^3^*H*, Pyr), 10.34 (bs, 1H, CON*H*), 12.72 (bs, 1H, N*H*) ppm; ^13^C-NMR (DMSO-*d*_6_): δ = 15.1 (S*C*H_3_), 25.3 (COCH_2_*C*H_2_), 36.0 (CO*C*H_2_CH_2_), 55.2 (C^5^O*C*H_3_), 55.8 (C^2^O*C*H_3_), 110.6 (*C*^3^H, Pyr), 111.2 (*C*^4^H, (OCH_3_)_2_-Phe), 111.5 (*C*^3^H, (OCH_3_)_2_-Phe), 115.6 (*C*^3/5^H, F-Phe), 115.9 (*C*^6^H, (OCH_3_)_2_-Phe), 116.5 (*C*^5^H, Pyr), 126.7 (*C*^1^, F-Phe), 130.0 (*C*^1^, (OCH_3_)_2_-Phe), 130.6 (*C*^5^, Imdz and *C*^2/6^H, F-Phe), 134.5 (*C*^4^, Imdz), 142.3 (*C*^2^, Imdz), 147.7 (*C*^4^ and *C*^6^H, Pyr), 151.2 (*C*^2^, (OCH_3_)_2_-Phe), 152.5 (*C*^2^, Pyr), 153.0 (*C*^5^, (OCH_3_)_2_-Phe), 161.9 (d, ^1^*J*_CF_ = 247.3, *C*F), 171.3 (*C*O) ppm; MS (ESI, 70 eV) *m*/*z* 494 [MH]^+^; HRMS (EI, 70 eV) *m*/*z* [M]^+^ calcd. for C_26_H_25_FN_4_O_3_S, 492.1631; found, 492.1631. The compound was demonstrated to have the desired structure by small molecule X-ray structure determination (Figure 6). For further details see CCDC and Supporting Information.

*3-(2,3-Dimethoxyphenyl)-N-(4-(5-(4-fluorophenyl)-2-(methylthio)-1H-imidazol-4-yl)-pyridin-2-yl)-propanamide* (**11c**). Synthesis was performed according to the general procedure from 3-(2,3-dimethoxyphenyl)propionic acid (700 mg, 3.33 mmol) and **7** (500 mg, 1.67 mmol). Purification was achieved by flash chromatography (SiO_2_, 30%–100% ethyl acetate/petrol ether and RP-18, 50%–100% methanol/H_2_O) to afford **11c** as colorless solid. Yield 515 mg (63%); C_26_H_25_FN_4_O_3_S (M*_r_* 492.57); ^1^H-NMR (DMSO-*d*_6_): δ = 2.62 (s, 3H, SC*H*_3_), 2.59–2.66 (m, 2H, COC*H*_2_CH_2_), 2.84 (t, ^3^*J* = 7.7 Hz, 2H, COCH_2_C*H*_2_), 3.73 (s, 3H, C^2^OC*H*_3_), 3.78 (s, 3H, C^3^OC*H*_3_), 6.79 (dd, ^3^*J* = 7.5 Hz, ^4^*J* = 1.6 Hz, 1H, C^6^*H*, (OCH_3_)_2_-Phe), 6.88 (dd, ^3^*J* = 8.1 Hz, ^4^*J* = 1.2 Hz, 1H, C^4^*H*, (OCH_3_)_2_-Phe), 6.97 (t, ^3^*J* = 7.8 Hz, 1H, C^5^*H*, (OCH_3_)_2_-Phe), 7.01 (dd, ^3^*J* = 5.3 Hz, ^4^*J* = 1.5 Hz, 1H, C^5^*H*, Pyr), 7.26–7.32 (m, 2H, C^3/5^*H*, F-Phe), 7.45–7.51 (m, 2H, C^2/6^*H*, F-Phe), 8.11 (d, ^3^*J* = 5.3 Hz, 1H, C^6^*H*, Pyr), 8.34 (bs, 1H, C^3^*H*, Pyr), 10.35 (s, 1H, CON*H*), 12.70 (s, 1H, N*H*) ppm; ^13^C-NMR (DMSO-*d*_6_): δ = 15.1 (S*C*H_3_), 24.8 (COCH_2_*C*H_2_), 36.8 (CO*C*H_2_CH_2_), 55.3 (C^3^O*C*H_3_), 60.0 (C^2^O*C*H_3_), 110.6 (*C*^3^H, Pyr), 110.9 (*C*^4^H, (OCH_3_)_2_-Phe), 115.8 (d, ^2^*J*_CF_ = 21.8 Hz, *C*^3/5^H, F-Phe), 116.4 (*C*^5^H, Pyr), 121.4 (*C*^6^H, (OCH_3_)_2_-Phe), 123.7 (*C*^5^H, (OCH_3_)_2_-Phe), 126.6 (d, ^4^*J*_CF_ = 3.1 Hz, *C*^1^, F-Phe), 130.1 (*C*^5^, Imdz), 130.7 (d, ^3^*J*_CF_ = 8.4 Hz, *C*^2/6^H, F-Phe), 134.4 (*C*^1^, (OCH_3_)_2_-Phe), 134.5 (*C*^4^, Imdz), 142.2 (*C*^2^, Imdz), 143.8 (*C*^4^, Pyr), 146.6 (*C*^2^OCH_3_), 147.6 (*C*^6^H, Pyr), 152.4 (*C*^3^OCH_3_), 152.5 (*C*^2^, Pyr), 162.0 (d, ^1^*J*_CF_ = 244.7 Hz, *C*F), 171.1 (*C*O) ppm; MS (ESI, 70 eV) *m*/*z* 493 [MH]^+^.

*3-(3,4-Dimethoxyphenyl)-N-(4-(5-(4-fluorophenyl)-2-(methylthio)-1H-imidazol-4-yl)-pyridin-2-yl)-propanamide* (**11d**). Synthesis was performed according to the general procedure from 3-(3,4-dimethoxyphenyl)propionic acid (700 mg, 3.33 mmol) and **7** (500 mg, 1.67 mmol). Purification was achieved by flash chromatography (SiO_2_, 30%–100% ethyl acetate/petrol ether and RP-18, 50%–100% methanol/H_2_O) to afford **11d** as colorless solid. Yield 468 mg (57%); ^1^H-NMR (DMSO-*d*_6_): δ = 2.62 (s, 3H, SC*H*_3_), 2.62–2.67 (m, 2H, COC*H*_2_CH_2_), 2.81 (t, ^3^*J* = 7.4 Hz, 2H, COCH_2_C*H*_2_), 3.70 (s, 3H, C^4^OC*H*_3_), 3.72 (s, 3H, C^3^OC*H*_3_), 6.73 (dd, ^3^*J* = 8.2 Hz, ^4^*J* = 1.9 Hz, 1H, C^6^*H*, (OCH_3_)_2_-Phe), 6.84 (d, ^3^*J* = 8.3 Hz, 1H, C^5^*H*, (OCH_3_)_2_-Phe), 6.85 (bs, 1H, C^2^*H*, (OCH_3_)_2_-Phe), 7.00 (dd, ^3^*J* = 5.2 Hz, ^4^*J* = 1.5 Hz, 1H, C^5^*H*, Pyr), 7.26–7.31 (m, 2H, C^3/5^*H*, F-Phe), 7.46–7.50 (m, 2H, C^2/6^*H*, F-Phe), 8.10–8.34 (m, 2H, C^3/6^*H* Pyr), 10.33 (s, 1H, CON*H*), 12.71 (s, 1H, N*H*) ppm; ^13^C-NMR (DMSO-*d*_6_): δ = 15.1 (S*C*H_3_), 30.4 (COCH_2_*C*H_2_), 38.0 (CO*C*H_2_CH_2_), 55.4 (C^4^O*C*H_3_), 55.5 (C^3^O*C*H_3_), 110.6 (*C*^3^H, Pyr), 111.9 (*C*^5^H, (OCH_3_)_2_-Phe), 112.3 (*C*^2^H, (OCH_3_)_2_-Phe), 115.8 (d, ^2^*J*_CF_ = 22.5 Hz, *C*^3/5^H, F-Phe), 116.4 (*C*^5^H, Pyr), 120.0 (*C*^6^H, (OCH_3_)_2_-Phe), 130.6 (*C*^2/6^H, F-Phe), 133.5 (*C*^1^, (OCH_3_)_2_-Phe), 147.1 (*C*^4^OCH_3_), 147.6 (*C*^6^H, Pyr), 148.6 (*C*^3^OCH_3_), 152.5 (*C*^2^, Pyr), 165.8 (d, ^1^*J*_CF_ = 242.6 Hz, *C*F), 171.2 (*C*O) ppm; MS (ESI, 70 eV) *m*/*z* 493 [MH]^+^.

*3-(3,4,5-Trimethoxyphenyl)-N-(4-(5-(4-fluorophenyl)-2-(methylthio)-1H-imidazol-4-yl)-pyridin-2-yl)-propanamide* (**11e**). Synthesis was performed according to the general procedure from 3-(3,4,5-dimethoxyphenyl)propionic acid (800 mg, 3.33 mmol) and **7** (500 mg, 1.67 mmol). Purification was achieved by flash chromatography (SiO_2_, 20%–100% ethyl acetate/petrol ether and RP-18, 50%–100% methanol/H_2_O) to afford **11e** as pale yellowish solid. Yield 422 mg (49%); C_27_H_27_FN_4_O_4_S (M*_r_* 522.60); ^1^H-NMR (DMSO-*d*_6_): δ = 2.62 (s, 3H, SC*H*_3_), 2.62–2.69 (m, 2H, COC*H*_2_CH_2_), 2.80–2.85 (m, 2H, COCH_2_C*H*_2_), 3.61 (s, 3H, C^4^OC*H*_3_), 3.74 (bs, 6H, C^3/5^OC*H*_3_), 6.56 (s, 2H, C^2/6^*H*, (OCH_3_)_3_-Phe), 7.01 (dd, ^3^*J* = 5.3 Hz, ^4^*J* = 1.6 Hz, 1H, C^5^*H*, Pyr), 7.26–7.32 (m, 2H, C^3/5^*H*, F-Phe), 7.46–7.51 (m, 2H, C^2/6^*H*, F-Phe), 8.11 (dd, ^3^*J* = 5.3 Hz, ^5^*J* = 0.5 Hz, 1H, C^6^*H*, Pyr), 8.36 (bs, 1H, C^3^*H*, Pyr), 10.36 (s, 1H, CON*H*), 12.70 (s, 1H, N*H*) ppm; ^13^C-NMR (DMSO-*d*_6_): δ = 15.1 (S*C*H_3_), 31.3 (COCH_2_*C*H_2_), 37.9 (CO*C*H_2_CH_2_), 55.7 (C^3/5^O*C*H_3_), 59.9 (C^4^O*C*H_3_), 105.5 (*C*^2/6^H, (OCH_3_)_3_-Phe), 110.6 (*C*^3^H, Pyr), 115.8 (d, ^2^*J*_CF_ = 21.7 Hz, *C*^3/5^H, F-Phe), 116.4 (*C*^5^H, Pyr), 126.6 (d, ^4^*J*_CF_ = 3.3 Hz, *C*^1^, F-Phe), 130.1 (*C*^5^, Imdz), 130.7 (d, ^3^*J*_CF_ = 8.4 Hz, *C*^2/6^H, F-Phe), 134.5 (*C*^4^, Imdz), 135.7 (*C*^4^OCH_3_), 136.8 (*C*^1^, (OCH_3_)_3_-Phe), 142.2 (*C*^2^, Imdz), 143.8 (*C*^4^, Pyr), 147.6 (*C*^6^H, Pyr), 152.5 (*C*^2^, Pyr), 152.7 (*C*^3/5^OCH_3_), 162.0 (d, ^1^*J*_CF_ = 245.4 Hz, *C*F), 171.2 (*C*O) ppm; MS (ESI, 70 eV) *m*/*z* 523 [MH]^+^.

#### 3.2.4. Synthesis of 4-(5-(4-Fluorophenyl)-2-(methylthio)-1*H*-imidazol-4-yl)pyridin-2-carbamides **12a**–**g**

A solution of **7** (1.0 equiv), the appropriate isocyanate (1.1 equiv) and DIPEA (1.2 equiv) in 5 mL anhyd. DMF was stirred under a nitrogen atmosphere at rt for 12 h. The solvent was removed under reduced pressure, the residue resuspended in ethyl acetate, and washed with 0.1 M aq. HCl, sat. aq. NaHCO_3_ solution, and sat. aq. NaCl solution, dried over anhyd. Na_2_SO_4_, and concentrated under reduced pressure. The crude product was purified by flash chromatography (SiO_2_ and RP-18, eluent and mixing ratio given for each compound, respectively) to afford the particular compound.

*1-(2,4-Dimethoxyphenyl)-3-(4-(5-(4-fluorophenyl)-2-(methylthio)-1H-imidazol-4-yl)-pyridin-2-yl)carbamide* (**12a**). Synthesis was performed according to the general procedure from 2,4-dimethoxyphenyl isocyanate (262 mg, 1.47 mmol). Purification was achieved by flash chromatography (SiO_2_, 0%–10% methanol/DCM and RP-18, 50%–100% methanol/H_2_O) to afford **12a** as colorless solid. Yield 135 mg (21%); C_24_H_22_FN_5_O_3_S (M*_r_* 479.53); m.p. 214 °C; ^1^H-NMR (DMSO-*d*_6_): δ = 2.63 (s, 3H, SC*H*_3_), 3.74 (s, 3H, C^4^OC*H*_3_), 3.88 (s, 3H, C^2^OC*H*_3_), 6.48 (dd, ^3^*J* = 8.9 Hz, ^4^*J* = 2.7 Hz, 1H, C^5^*H*, (OCH_3_)_2_-Phe), 6.62 (d, ^4^*J* = 2.7 Hz, 1H, C^3^*H*, (OCH_3_)_2_-Phe), 6.92 (dd, ^3^*J* = 5.4 Hz, ^4^*J* = 1.5 Hz, 1H, C^5^*H*, Pyr), 7.30–7.51 (m, 5H, C^3/5^*H*, F-Phe and C^3^*H*, Pyr and C^2/6^*H*, F-Phe), 8.03 (d, ^3^*J* = 8.9 Hz, 1H, C^6^*H*, (OCH_3_)_2_-Phe), 8.12 (bs, 1H, C^6^*H*, Pyr), 9.64 (bs, 1H, N*H*), 10.89–11.26 (m, 1H, N*H*), 12.74 (bs, 1H, N*H*, Imdz) ppm; ^13^C-NMR (DMSO-*d*_6_): δ = 15.1 (S*C*H_3_), 55.3 (C^4^O*C*H_3_), 56.0 (C^2^O*C*H_3_), 98.8 (*C*^3^H, (OCH_3_)_2_-Phe), 104.1 (*C*^5^H, (OCH_3_)_2_-Phe), 108.6 (*C*^3^H, Pyr), 114.6 (*C*^5^H, Pyr), 115.7 (*C*^2/6^H, F-Phe), 119.7 (*C*^6^H, (OCH_3_)_2_-Phe), 121.9 (*C*^1^, (OCH_3_)_2_-Phe), 130.7 (*C*^3/5^H, F-Phe), 134.2 (*C*^4^, Pyr), 142.3 (*C*^2^, Imdz), 146.1 (*C*^6^H, Pyr), 149.4 (*C*^2^, (OCH_3_)_2_-Phe), 152.2 (*C*O), 153.6 (*C*^2^, Pyr), 155.2 (*C*^4^, (OCH_3_)_2_-Phe), 161.8 (d, ^1^*J*_CF_ = 241.6 Hz, *C*F) ppm; MS (ESI, 70 eV) *m*/*z* 480 [MH]^+^.

*1-(2,5-Dimethoxyphenyl)-3-(4-(5-(4-fluorophenyl)-2-(methylthio)-1H-imidazol-4-yl)-pyridin-2-yl)carbamide* (**12b**). Synthesis was performed according to the general procedure from 2,5-dimethoxyphenyl isocyanate (300 mg, 1.67 mmol). Purification was achieved by flash chromatography (SiO_2_, 20%–100% ethyl acetate/petrol ether) to afford **12b** as pale yellowish solid. Yield 79.9 mg (15%); C_24_H_22_FN_5_O_3_S (M*_r_* 479.53); m.p. 184 °C; ^1^H-NMR (DMSO-*d*_6_): δ = 2.63 (s, 3H, SC*H*_3_), 3.70 (s, 3H, C^5^OC*H*_3_), 3.84 (s, 3H, C^2^OC*H*_3_), 6.52 (dd, ^3^*J* = 8.8 Hz, ^4^*J* = 2.9 Hz, 1H, C^4^*H*, (OCH_3_)_2_-Phe), 6.91–6.96 (m, 1H, C^3^*H*, (OCH_3_)_2_-Phe), 6.95 (dd, ^3^*J* = 5.5 Hz, ^4^*J* = 1.4 Hz, 1H, C^5^*H*, Pyr), 7.28–7.38 (m, 2H, C^3/5^*H*, F-Phe), 7.47–7.51 (m, 3H, C^2/6^*H*, F-Phe and C^3^*H*, Pyr), 7.92 (d, ^4^*J* = 2.9 Hz, 1H, C^6^*H*, (OCH_3_)_2_-Phe), 8.13 (d, ^3^*J* = 5.4 Hz, 1H, C^6^*H*, Pyr), 9.76 (bs, 1H, N*H*), 11.52 (vbs, 1H, N*H*), 12.74 (bs, 1H, N*H*, Imdz) ppm; ^13^C-NMR (DMSO-*d*_6_): δ = 15.1 (S*C*H_3_), 55.3 (C^5^O*C*H_3_), 55.6 (C^2^O*C*H_3_), 105.6 (*C*^6^H, (OCH_3_)_2_-Phe), 105.8 (*C*^4^H, (OCH_3_)_2_-Phe), 108.5 (*C*^3^H, Pyr), 111.7 (*C*^3^H, (OCH_3_)_2_-Phe), 114.7 (*C*^5^H, Pyr), 115.9 (d, ^2^*J*_CF_ = 22.0 Hz, *C*^3/5^H, F-Phe), 126.5 (d, ^4^*J*_CF_ = 3.3 Hz, *C*^1^, F-Phe), 129.6 (*C*^1^, (OCH_3_)_2_-Phe), 130.4 (*C*^5^, Imdz), 130.8 (d, ^3^*J*_CF_ = 8.4 Hz, *C*^2/6^H, F-Phe), 134.1 (*C*^4^, Imdz), 142.3 (*C*^2^, Imdz), 142.4 (*C*^2^OCH_3_), 144.2 (*C*^4^, Pyr), 146.1 (*C*^6^H, Pyr), 152.2 (*C*O), 153.4 (*C*^5^OCH_3_), 153.4 (*C*^2^, Pyr), 162.1 (d, ^1^*J*_CF_ = 245.9 Hz, *C*F) ppm; MS (ESI, 70 eV) *m*/*z* 480 [MH]^+^.

*1-(4-(5-(4-Fluorophenyl)-2-(methylthio)-1H-imidazol-4-yl)pyridin-2-yl)-3-(4-methoxy-phenyl)carbamide* (**12c**). Synthesis was performed according to the general procedure from 4-methoxyphenyl isocyanate (165 µL, 1.27 mmol). Purification was achieved by flash chromatography (SiO_2_, 50%–100% ethyl acetate/petrol ether and RP-18, 60%–100% methanol/H_2_O) to afford **12c** as pale yellow solid. Yield 137 mg (26%); C_23_H_20_FN_5_O_2_S (M*_r_* 449.50); m.p. 201 °C; ^1^H-NMR (DMSO-*d*_6_): δ = 2.62 (s, 3H, SC*H*_3_), 3.72 (s, 3H, OC*H*_3_), 6.86–6.90 (m, 2H, C^3/5^*H*, H_3_CO-Phe), 6.92 (dd, ^3^*J* = 5.4 Hz, ^4^*J* = 1.5 Hz, 1H, C^5^*H*, Pyr), 7.30 (bs, 2H, C^3/5^*H*, F-Phe), 7.39–7.44 (m, 2H, C^2/6^*H*, H_3_CO-Phe), 7.47–7.51 (m, 2H, C^2/6^*H*, F-Phe), 7.58 (bs, 1H, C^3^*H*, Pyr), 8.11 (bs, 1H, C^6^*H*, Pyr), 9.40 (bs, 1H, N*H*), 10.35–10.71 (m, 1H, N*H*), 12.74 (s, 1H, N*H*, Imdz) ppm; ^13^C-NMR (DMSO-*d*_6_): δ = 15.1 (S*C*H_3_), 55.2 (O*C*H_3_), 105.3 (*C*^4^, Pyr), 108.7 (*C*^3^H, Pyr), 114.0 (*C*^3/5^H, H_3_CO-Phe), 114.6 (*C*^5^H, Pyr), 115.8 (d, ^2^*J*_CF_ = 22.0 Hz, *C*^3/5^H, F-Phe), 120.6 (*C*^2/6^H, H_3_CO-Phe), 130.6–130.8 (*C*^2/6^H, F-Phe), 132.0 (*C*^1^, H_3_CO-Phe), 142.3 (*C*^2^, Imdz), 146.4 (*C*^6^H, Pyr), 152.3 (*C*O), 153.5 (*C*^2^, Pyr), 154.9 (*C*^4^, H_3_CO-Phe), 162.1 (d, ^1^*J*_CF_ = 252.6 Hz, *C*F) ppm; MS (ESI, 70 eV) *m*/*z* 450 [MH]^+^.

*1-(4-(5-(4-Fluorophenyl)-2-(methylthio)-1H-imidazol-4-yl)pyridin-2-yl)-3-(m-tolyl)-carbamide* (**12d**). Synthesis was performed according to the general procedure from 3-methylphenyl isocyanate (284 µL, 2.20 mmol). Purification was achieved by flash chromatography (SiO_2_, 30%–100% ethyl acetate/petrol ether and RP-18, 60%–80% methanol/H_2_O) to afford **12d** as colorless solid. Yield 241 mg (28%); C_23_H_20_FN_5_OS (M*_r_* 433.51); m.p. 211 °C; ^1^H-NMR (DMSO-*d*_6_): δ = 2.29 (s, 3H, C*H*_3_), 2.63 (s, 3H, SC*H*_3_), 6.83 (d, ^3^*J* = 7.4 Hz, 1H, C^4^*H*, Tol), 6.95 (dd, ^3^*J* = 5.4 Hz, ^4^*J* = 1.5 Hz, 1H, C^5^*H*, Pyr), 7.15–7.35 (m, 2H, C^3/5^*H*, F-Phe), 7.17 (t, ^3^*J* = 7.6 Hz, 1H, C^5^*H*, Tol), 7.31 (d, ^3^*J* = 8.0 Hz, 1H, C^6^*H*, Tol), 7.35 (s, 1H, C^2^*H*, Tol), 7.47–7.52 (m, 2H, C^2/6^*H*, F-Phe), 7.61 (bs, 1H, C^3^*H*, Pyr), 8.13 (bs, 1H, C^6^*H*, Pyr), 9.44 (s, 1H, N*H*), 10.42–10.78 (m, 1H, N*H*), 12.74 (bs, 1H, N*H*, Imdz) ppm; ^13^C-NMR (DMSO-*d*_6_): δ = 15.1 (S*C*H_3_), 21.2 (*C*H_3_), 108.7 (*C*^3^H, Pyr), 114.8 (*C*^5^H, Pyr), 115.8 (d, ^2^*J*_CF_ = 22.3 Hz, *C*^3/5^H, F-Phe), 116.0 (*C*^6^H, Tol), 119.3 (*C*^2^H, Tol), 123.2 (*C*^4^H, Tol), 126.6 (d, ^4^*J*_CF_ = 2.6 Hz, *C*^1^, F-Phe), 128.7 (*C*^5^H, Tol), 129.7 (*C*^5^, Imdz), 130.8 (*C*^2/6^H, F-Phe), 134.2 (*C*^4^, Imdz), 138.1 (*C*^3^, Tol), 139.0 (*C*^1^, Tol), 142.4 (*C*^2^, Imdz), 144.3 (*C*^4^, Pyr), 146.4 (*C*^6^H, Pyr), 152.1 (*C*O), 153.4 (*C*^2^, Pyr), 162.0 (d, ^1^*J*_CF_ = 243.0 Hz, *C*F) ppm; MS (ESI, 70 eV) *m*/*z* 434 [MH]^+^.

*1-(3-Chloro-4-methylphenyl)-3-(4-(5-(4-fluorophenyl)-2-(methylthio)-1H-imidazol-4-yl)-pyridin-2-yl)-carbamide* (**12e**). Synthesis was performed according to the general procedure from 3-chloro-4-methylyphenyl isocyanate (205 µL, 1.47 mmol). Purification was achieved by flash chromatography (SiO_2_, 0%–10% methanol/DCM and RP-18, 70%–100% methanol/H_2_O and SiO_2_, 20%–100% ethyl acetate/petrol ether) to afford **12e** as colorless solid. Yield 210 mg (34%); C_23_H_19_ClFN_5_OS (M*_r_* 467.95); m.p. 210 °C; ^1^H-NMR (DMSO-*d*_6_): δ = 2.27 (s, 3H, C*H*_3_), 2.63 (s, 3H, SC*H*_3_), 6.96 (dd, ^3^*J* = 5.5 Hz, ^4^*J* = 1.4 Hz, 1H, C^5^*H*, Pyr), 7.26 (m, 2H, C^5/6^*H*, Cl-Tol), 7.28–7.34 (m, 2H, C^3/5^*H*, F-Phe), 7.46–7.50 (m, 2H, C^2/6^*H*, F-Phe), 7.59 (bs, 1H, C^3^*H*, Pyr), 7.77 (s, 1H, C^2^*H*, Cl-Tol), 8.12 (d, ^3^*J* = 5.4 Hz, 1H, C^6^*H*, Pyr), 9.51 (s, 1H, N*H*), 10.99 (s, 1H, N*H*), 12.73 (s, 1H, N*H*, Imdz) ppm; ^13^C-NMR (DMSO-*d*_6_): δ = 15.1 (S*C*H_3_), 18.8 (*C*H_3_), 108.7 (*C*^3^H, Pyr), 114.8 (*C*^5^H, Pyr), 115.9 (d, ^2^*J*_CF_ = 21.7 Hz, *C*^3/5^H, F-Phe), 117.6 (*C*^6^H, Cl-Tol), 118.7 (*C*^2^H, Cl-Tol), 126.5 (d, ^4^*J*_CF_ = 3.1 Hz, *C*^1^, F-Phe), 128.9 (*C*^4^, Cl-Tol), 130.4 (*C*^5^, Imdz), 130.8 (d, ^3^*J*_CF_ = 8.3 Hz, *C*^2/6^H, F-Phe), 131.2 (*C*^5^H, Cl-Tol), 133.2 (*C*^3^, Cl-Tol), 134.1 (*C*^4^, Imdz), 138.2 (*C*^1^, Cl-Tol), 142.3 (*C*^2^, Imdz), 144.3 (*C*^4^, Pyr), 146.4 (*C*^6^H, Pyr), 152.2 (*C*O), 153.2 (*C*^2^, Pyr), 162.1 (d, ^1^*J*_CF_ = 245.5 Hz, *C*F) ppm; MS (ESI, 70 eV) *m*/*z* 468 [MH]^+^.

*1-(4-(5-(4-Fluorophenyl)-2-(methylthio)-1H-imidazol-4-yl)pyridin-2-yl)-3-(3-(trifluoromethyl)phenyl)-carbamide* (**12f**). Synthesis was performed according to the general procedure from 3-(trifluoromethyl)phenyl isocyanate (300 µL, 2.20 mmol). Purification was achieved by flash chromatography (SiO_2_, 40% methanol/DCM) and subsequent filtration to afford **12f** as colorless solid. Yield 255 mg (26%); C_23_H_17_FN_5_OS (M*_r_* 487.48); m.p. 234 °C; ^1^H-NMR (DMSO-*d*_6_): δ = 2.63 (s, 3H, SC*H*_3_), 6.99 (dd, ^3^*J* = 5.6 Hz, ^4^*J* = 1.2 Hz, 1H, C^5^*H*, Pyr), 7.28–7.37 (m, 3H, C^3/5^*H*, F-Phe and C^4^*H*, F_3_C-Phe), 7.46–7.66 (m, 5H, C^3^*H*, Pyr and C^2/6^*H*, F-Phe and C^5/6^*H*, F_3_C-Phe), 8.08 (bs, 1H, C^2^*H*, F_3_C-Phe), 8.14 (d, ^3^*J* = 5.5 Hz, 1H, C^6^*H*, Pyr), 9.56 (s, 1H, N*H*), 11.12 (s, 1H, N*H*), 12.74 (s, 1H, N*H*, Imdz) ppm; ^13^C-NMR (DMSO-*d*_6_): δ = 15.1 (S*C*H_3_), 108.7 (*C*^3^H, Pyr), 114.7 (*C*^2^H, F_3_C-Phe), 115.0 (*C*^5^H, Pyr), 115.9 (d, ^2^*J*_CF_ = 21.8 Hz, *C*^3/5^H, F-Phe), 118.7 (*C*^4^H, F_3_C-Phe), 122.5 (*C*^6^H, F_3_C-Phe), 124.2 (q, ^1^*J*_CF_ = 272.1 Hz, *C*F_3_, F_3_C-Phe), 126.5 (d, ^4^*J*_CF_ = 2.9 Hz, *C*^1^, F-Phe), 129.6 (q, ^2^*J*_CF_ = 31.3 Hz, *C*^3^, F_3_C-Phe), 130.0 (*C*^5^H, F_3_C-Phe), 130.5 (*C*^5^, Imdz), 130.8 (d, ^3^*J*_CF_ = 8.3 Hz, *C*^2/6^H, F-Phe), 134.0 (*C*^4^, Imdz), 139.9 (*C*^1^, F_3_C-Phe), 142.3 (*C*^2^, Imdz), 144.3 (*C*^4^, Pyr), 146.6 (*C*^6^H, Pyr), 152.2 (*C*O), 153.0 (*C*^2^, Pyr), 162.0 (d, ^1^*J*_CF_ = 245.2 Hz, *C*F) ppm; MS (ESI, 70 eV) *m*/*z* 488 [MH]^+^; HRMS (EI, 70 eV) *m*/*z* [M]^+^ calcd. for C_23_H_17_FN_5_OS, 487.1090; found 487.1090.

*1-(4-(5-(4-Fluorophenyl)-2-(methylthio)-1H-imidazol-4-yl)pyridin-2-yl)-3-(naphthalen-1-yl)carbamide* (**12g**). Synthesis was performed according to the general procedure from naphthyl isocyanate (211 µL, 1.47 mmol). Purification was achieved by flash chromatography (SiO_2_, 0%–10% methanol/DCM and SiO_2_, 30%–100% ethyl acetate/petrol ether and RP-18, 50%–100% methanol/H_2_O) to afford **12g** as pale yellow solid. Yield 257 mg (41%); C_26_H_20_FN_5_OS (M*_r_* 469.54); m.p. 249 °C; ^1^H-NMR (DMSO-*d*_6_): δ = 2.64 (s, 3H, SC*H*_3_), 7.01 (dd, ^3^*J* = 5.5 Hz, ^4^*J* = 1.4 Hz, 1H, C^5^*H*, Pyr), 7.30–7.36 (m, 2H, C^3/5^*H*, F-Phe), 7.42–7.59 (m, 5H, C^3^*H*, Pyr and C^2/6^*H*, F-Phe and C^3/6^*H*, Naph), 7.63–7.68 (m, 2H, C^4/7^*H*, Naph), 7.95 (d, ^3^*J* = 8.1 Hz, 1H, C^5^*H*, Naph), 8.16–8.22 (m, 2H, C^2/8^*H*, Naph), 8.27 (d, ^3^*J* = 5.5 Hz, 1H, C^6^*H*, Pyr), 9.87 (s, 1H, N*H*), 11.86 (bs, 1H, N*H*), 12.77 (s, 1H, N*H*, Imdz) ppm; ^13^C-NMR (DMSO-*d*_6_): δ = 15.1 (S*C*H_3_), 108.7 (*C*^3^H, Pyr), 114.7 (*C*^5^H, Pyr), 115.9 (d, ^2^*J*_CF_ = 21.7 Hz, *C*^3/5^H, F-Phe), 116.7 (*C*^2^H, Naph), 120.9 (*C*^8^H, Naph), 123.0 (*C*^4^H, Naph), 125.4 (*C*^1^, Naph), 126.0 (*C*^3/6^H, Naph), 126.3 (*C*^7^H, Naph), 126.5 (d, ^4^*J*_CF_ = 3.2 Hz, *C*^1^, F-Phe), 128.6 (*C*^5^H, Naph), 130.6 (*C*^5^, Imdz), 130.8 (d, ^3^*J*_CF_ = 8.3 Hz, *C*^2/6^H, F-Phe), 133.7 (*C*^4a^, Naph), 134.0 (*C*^8a^, Naph), 134.2 (*C*^4^, Imdz), 142.4 (*C*^2^, Imdz), 144.5 (*C*^4^, Pyr), 146.1 (*C*^6^H, Pyr), 152.6 (*C*O), 153.5 (*C*^2^, Pyr), 162.1 (d, ^1^*J*_CF_ = 246.2 Hz, *C*F) ppm; MS (ESI, 70 eV) *m*/*z* 471 [MH]^+^.

#### 3.2.5. Synthesis of *N*-(4-(5-(4-Fluorophenyl)-2-(methylthio)-1*H*-imidazol-4-yl)pyridin-2-yl)-1-methyl-4-phenyl-1*H*-pyrrole-2-carboxamides **13**, **14a**–**b**, **15a**–**b**, **16a**–**b**

*Ethyl 4-bromo-1-methyl-1H-pyrrole-2-carboxylate* (**13**). NaH (240 mg 60% dispersion in mineral oil, 6.05 mmol) was added in one portion to a solution of 4-bromo-1*H*-pyrrole-2-carboxylate (1.17 g, 5.34 mmol) in 15 mL anhyd. DMF at 0 °C under a nitrogen atmosphere. The suspension was stirred for 20 min at the same temp. before methyl iodide (400 µL, 6.43 mmol) was carefully added and stirring continued at 0 °C for 15 min and another 2.5 h at r.t. The reaction was quenched with H_2_O and extracted with ethyl acetate. The combined organic phases were washed with H_2_O and sat. aq. NaCl solution, dried over anhyd. Na_2_SO_4_, and the solvent was removed under reduced pressure. Purification was achieved by flash chromatography (SiO_2_, 2%–10% ethyl acetate/petrol ether) to afford **13** as clear colorless oil that crystallized on standing as colorless needles. Yield 1.22 g (98%); C_8_H_10_BrNO_2_ (M*_r_* 232.08); m.p. 43 °C; ^1^H-NMR (DMSO-*d*_6_): δ = 1.26 (t, ^3^*J* = 7.1 Hz, 3H, COOCH_2_C*H*_3_), 3.83 (d, *J* = 0.2 Hz, 3H, C*H*_3_), 4.21 (q, ^3^*J* = 7.1 Hz, 2H, COOC*H*_2_CH_3_), 6.84 (d, ^4^*J* = 2.0 Hz, 1H, C^3^*H*, Pyrrole), 7.28 (d, ^4^*J* = 1.7 Hz, 1H, C^5^*H*, Pyrrole) ppm; ^13^C-NMR (DMSO-*d*_6_): δ = 14.2 (COOCH_2_*C*H_3_), 36.6 (*C*H_3_), 59.8 (COO*C*H_2_CH_3_), 93.8 (*C*Br), 118.1 (*C*^3^H, Pyrrole), 122.7 (*C*^2^, Pyrrole), 129.5 (*C*^5^H, Pyrrole), 159.5 (*C*OOCH_2_CH_3_) ppm.

*Ethyl 4-(2,4-dimethoxyphenyl)-1-methyl-1H-pyrrole-2-carboxylate* (**14a**). 27 mL 2 M aq. Na_2_CO_3_ solution were added to a stirred solution of **13** (1.22 g, 5.26 mmol), 2,4-dimethoxyphenyl boronic acid (2.89 g, 15.9 mmol), and Pd(PPh_3_)_4_ (306 mg, 265 µmol) in 80 mL DMF. Stirring continued for 4 h under reflux and 12 h at r.t. H_2_O was added and the mixture was extracted with ethyl acetate. The combined organic phases were washed with H_2_O and sat. aq. NaCl solution, dried over anhyd. Na_2_SO_4_, and the solvent was removed under reduced pressure. The crude product was purified by flash chromatography (SiO_2_, 5%–10% ethyl acetate/petrol ether and RP-18, 50%–70% methanol/H_2_O) to afford **14a** as colorless solid. Yield 1.02 g (67%); C_16_H_19_NO_4_ (M*_r_* 289.33); ^1^H-NMR (DMSO-*d*_6_): δ = 1.29 (t, ^3^*J* = 7.1 Hz, 3H, COOCH_2_C*H*_3_), 3.77 (s, 3H, C^4^OC*H*_3_), 3.84 (s, 3H, C^2^OC*H*_3_), 3.87 (s, 3H, C*H*_3_), 4.23 (q, ^3^*J* = 7.1 Hz, 2H, COOC*H*_2_CH_3_), 6.53 (dd, ^3^*J* = 8.5 Hz, ^4^*J* = 2.5 Hz, 1H, C^5^*H*, (OCH_3_)_2_-Phe), 6.60 (d, ^4^*J* = 2.5 Hz, 1H, C^3^*H*, (OCH_3_)_2_-Phe), 7.17 (d, ^4^*J* = 2.0 Hz, 1H, C^3^*H*, Pyrrole), 7.44 (d, ^3^*J* = 8.5 Hz, 1H, C^6^*H*, (OCH_3_)_2_-Phe), 7.46 (dd, ^4^*J* = 2.0 Hz, ^5^*J* = 0.4 Hz, 1H, C^5^*H*, Pyrrole) ppm; ^13^C-NMR (DMSO-*d*_6_): δ = 14.4 (COOCH_2_*C*H_3_), 36.4 (*C*H_3_), 55.2 (C^4^O*C*H_3_), 55.4 (C^2^O*C*H_3_), 59.4 (*C*H_2_), 98.8 (*C*^3^H, (OCH_3_)_2_-Phe), 105.2 (*C*^5^H, (OCH_3_)_2_-Phe), 115.4 (*C*^3^H, Pyrrole), 115.5 (*C*^1^, (OCH_3_)_2_-Phe), 119.0 (*C*^4^, Pyrrole), 121.3 (*C*^2^, Pyrrole), 127.7 (*C*^6^H, (OCH_3_)_2_-Phe), 129.0 (*C*^5^H, Pyrrole), 156.7 (*C*^4^OCH_3_), 158.74 (*C*^2^OCH_3_), 160.5 (*C*OOCH_2_CH_3_) ppm; MS (ESI, 70 eV) *m*/*z* 290 [MH]^+^.

*Ethyl 4-(2,5-dimethoxyphenyl)-1-methyl-1H-pyrrole-2-carboxylate* (**14b**). Synthesis was performed according to the procedure described for **14a** starting from **13** (1.11 g, 4.78 mmol), 2,5-dimethoxyphenyl boronic acid (2.65 g, 14.6 mmol), Pd(PPh_3_)_4_ (289 mg, 250 µmol) in 80 mL DMF, and 25 mL 2 M aq. Na_2_CO_3_ solution. Purification was achieved by flash chromatography (SiO_2_, 2%–10% ethyl acetate/petrol ether and RP-18, 65% methanol/H_2_O) to afford **14b** as colorless solid. Yield 984 mg (71%); C_16_H_19_NO_4_ (M*_r_* 289.33); ^1^H-NMR (DMSO-*d*_6_): δ = 1.30 (t, ^3^*J* = 7.1 Hz, 3H, COOCH_2_C*H*_3_), 3.74 (s, 3H, C^5^OC*H*_3_), 3.79 (s, 3H, C^2^OC*H*_3_), 3.89 (s, 3H, C*H*_3_), 4.24 (q, ^3^*J* = 7.1 Hz, 2H, COOC*H*_2_CH_3_), 6.74 (dd, ^3^*J* = 8.9 Hz, ^4^*J* = 3.1 Hz, 1H, C^4^*H*, (OCH_3_)_2_-Phe), 6.96 (d, ^3^*J* = 8.9 Hz, 1H, C^3^*H*, (OCH_3_)_2_-Phe), 7.09 (d, ^4^*J* = 3.1 Hz, 1H, C^6^*H*, (OCH_3_)_2_-Phe), 7.28 (d, ^4^*J* = 2.0 Hz, 1H, C^3^*H*, Pyrrole), 7.60 (dd, ^4^*J* = 2.0 Hz, ^5^*J* = 0.4 Hz, 1H, C^5^*H*, Pyrrole) ppm; ^13^C-NMR (DMSO-*d*_6_): δ = 14.3 (COOCH_2_*C*H_3_), 36.5 (*C*H_3_), 55.4 (C^5^O*C*H_3_), 55.9 (C^2^O*C*H_3_), 59.4 (COO*C*H_2_CH_3_), 111.7 (*C*^4^H, (OCH_3_)_2_-Phe), 112.5 (*C*^6^H, (OCH_3_)_2_-Phe), 112.9 (*C*^3^H, (OCH_3_)_2_-Phe), 116.1 (*C*^3^H, Pyrrole), 118.9 (*C*^4^, Pyrrole), 121.6 (*C*^1^, Pyrrole), 123.4 (*C*^1^, (OCH_3_)_2_-Phe), 130.0 (*C*^5^H, Pyrrole), 150.0 (*C*^2^OCH_3_), 153.4 (*C*^5^OCH_3_), 160.5 (*C*OOCH_2_CH_3_) ppm; MS (ESI, 70 eV) *m*/*z* 290 [MH]^+^.

*4-(2,4-Dimethoxyphenyl)-1-methyl-1H-pyrrole-2-carboxylic acid* (**15a**). 7 mL 4 M aq. NaOH were added to a solution of **14a** (990 mg, 3.42 mmol) in 18 mL THF and 9 mL methanol and the mixture was stirred at 50 °C for 5 h and then 12 h at r.t. H_2_O was added to the reaction, the pH was adjusted to 3 using 1 M aq. HCl, and the mixture was extracted with ethyl acetate. The combined organic phases were washed with H_2_O and sat. aq. NaCl solution, the organic phase was dried over anhyd. Na_2_SO_4_, and the solvent was removed under reduced pressure to afford **15a** as brown solid. Yield 894 mg (quant.); C_14_H_15_NO_4_ (M*_r_* 261.28); m.p. 164 °C; ^1^H-NMR (DMSO-*d*_6_): δ = 3.76 (s, 3H, C^4^OC*H*_3_), 3.83 (s, 3H, C^2^OC*H*_3_), 3.86 (s, 3H, C*H*_3_), 6.53 (dd, ^3^*J* = 8.5 Hz, ^4^*J* = 2.4 Hz, 1H, C^5^*H*, (OCH_3_)_2_-Phe), 6.59 (d, ^4^*J* = 2.4 Hz, 1H, C^3^*H*, (OCH_3_)_2_-Phe), 7.14 (d, ^4^*J* = 2.0 Hz, 1H, C^3^*H*, Pyrrole), 7.41 (d, ^4^*J* = 2.0 Hz, 1H, C^5^*H*, Pyrrole), 7.42 (d, ^3^*J* = 8.4 Hz, 1H, C^6^*H*, (OCH_3_)_2_-Phe), 12.17 (bs, 1H, COO*H*) ppm; ^13^C-NMR (DMSO-*d*_6_): δ = 36.3 (*C*H_3_), 55.2 (C^4^O*C*H_3_), 55.4 (C^2^O*C*H_3_), 98.8 (*C*^3^H, (OCH_3_)_2_-Phe), 105.2 (*C*^5^H, (OCH_3_)_2_-Phe), 115.7 (*C*^3^H, Pyrrole), 115.7 (*C*^1^, (OCH_3_)_2_-Phe), 118.8 (*C*^4^, Pyrrole), 122.0 (*C*^2^, Pyrrole), 127.6 (*C*^6^H, (OCH_3_)_2_-Phe), 128.6 (*C*^5^H, Pyrrole), 156.7 (*C*^2^OCH_3_), 158.7 (*C*^4^OCH_3_), 162.1 (*C*OOH) ppm; MS (ESI, 70 eV) *m*/*z* 262 [MH]^+^.

*4-(2,5-Dimethoxyphenyl)-1-methyl-1H-pyrrole-2-carboxylic acid* (**15b**). Synthesis was performed according to the procedure described for **15a** starting from **14b** (980 mg, 3.39 mmol) in 19 mL THF and 10 mL methanol, and 7 mL 4 M aq. NaOH to afford **15b** as brown solid. Yield 885 mg (quant.); C_14_H_15_NO_4_ (M*_r_* 261.28); m.p. 149 °C; ^1^H-NMR (DMSO-*d*_6_): δ = 3.74 (s, 3H, C^2^OC*H*_3_), 3.79 (s, 3H, C^5^OC*H*_3_), 3.88 (C*H*_3_), 6.72 (dd, ^3^*J* = 8.9 Hz, ^4^*J* = 3.1 Hz, 1H, C^4^*H*, (OCH_3_)_2_-Phe), 6.95 (d, ^3^*J* = 8.9 Hz, 1H, C^3^*H*, (OCH_3_)_2_-Phe), 7.08 (d, ^4^*J* = 3.1 Hz, 1H, C^6^*H*, (OCH_3_)_2_-Phe), 7.25 (d, ^4^*J* = 2.0 Hz, 1H, C^3^*H*, Pyrrole), 7.55 (d, ^4^*J* = 2.0 Hz, 1H, C^5^*H*, Pyrrole), 12.15 (bs, 1H, COO*H*) ppm; ^13^C-NMR (DMSO-*d*_6_): δ = 36.4 (*C*H_3_), 55.4 (C^2^O*C*H_3_), 55.9 (C^5^O*C*H_3_), 111.5 (*C*^4^H, (OCH_3_)_2_-Phe), 112.5 (*C*^6^H, (OCH_3_)_2_-Phe), 112.9 (*C*^3^H, (OCH_3_)_2_-Phe), 116.2 (*C*^3^H, Pyrrole), 118.7 (*C*^4^, Pyrrole), 122.4 (*C*^2^, Pyrrole), 123.6 (*C*^1^, (OCH_3_)_2_-Phe), 129.6 (*C*^5^H, Pyrrole), 150.1 (*C*^5^OCH_3_), 153.4 (*C*^2^OCH_3_), 162.1 (*C*OOH) ppm; MS (ESI, 70 eV) *m*/*z* 262 [MH]^+^.

*4-(2,4-Dimethoxyphenyl)-N-(4-(5-(4-fluorphenyl)-2-(methylthio)-1H-imidazol-4-yl)-pyridin-2-yl)-1-methyl-1H-pyrrole-2-carboxamide* (**16a**). A solution of **15a** (1.01 g, 4.07 mmol), PyBOP (2.54 g, 4.88 mmol), and DIPEA (2.15 mL, 12.3 mmol) was stirred in 14 mL anhyd. DMF under a nitrogen atmosphere for 30 min at r.t. **7** (1.60 g, 5.31 mmol) was added in one portion and the mixture was stirred for 12 h at 110 °C. The reaction was quenched with H_2_O and extracted with ethyl acetate. The combined organic phases were washed with H_2_O and sat. aq. NaCl solution, dried over anhyd. Na_2_SO_4_, and the solvent was removed under reduced pressure. The crude product was purified by flash chromatography (SiO_2_, 30%–100% ethyl acetate/petrol ether and RP-18, 50%–100% methanol/H_2_O) to afford **16a** as beige solid. Yield 303 mg (24%); C_29_H_26_FN_5_O_3_S (M*_r_* 543.62); m.p. 236 °C; ^1^H-NMR (DMSO-*d*_6_): δ = 2.64 (s, 3H, SC*H*_3_), 3.78 (s, 3H, C^2^OC*H*_3_), 3.86 (s, 3H, C^4^OC*H*_3_), 3.90 (s, 3H, C*H*_3_), 6.57 (dd, ^3^*J* = 8.5 Hz, ^4^*J* = 2.3 Hz, 1H, C^5^*H*, (OCH_3_)_2_-Phe), 6.61 (d, ^4^*J* = 2.3 Hz, 1H, C^3^*H*, (OCH_3_)_2_-Phe), 7.06 (dd, ^3^*J* = 5.3 Hz, ^4^*J* = 1.2 Hz, 1H, C^5^*H*, Pyr), 7.28–7.34 (m, 2H, C^3/5^*H*, F-Phe), 7.41 (d, ^4^*J* = 1.3 Hz, 1H, C^3^*H*, Pyrrole), 7.46 (d, ^3^*J* = 8.4 Hz, 1H, C^6^*H*, (OCH_3_)_2_-Phe), 7.49–7.54 (m, 2H, C^2/6^*H*, F-Phe), 7.66 (d, ^4^*J* = 1.4 Hz, 1H, C^5^*H*, Pyrrole), 8.18 (d, ^3^*J* = 5.2 Hz, 1H, C^6^*H*, Pyr), 8.38 (bs, 1H, C^3^*H*, Pyr), 10.10 (bs, 1H, CON*H*), 12.73 (bs, 1H, N*H*) ppm; ^13^C-NMR (DMSO-*d*_6_): δ = 15.3 (S*C*H_3_), 36.5 (*C*H_3_), 55.2 (C^2^O*C*H_3_), 55.5 (C^4^O*C*H_3_), 98.8 (*C*^3^H, (OCH_3_)_2_-Phe), 105.2 (*C*^5^H, (OCH_3_)_2_-Phe), 111.3 (*C*^3^H, Pyr), 113.2 (*C*^5^H, Pyrrole), 115.9 (d, ^2^*J*_CF_ = 21.9 Hz, *C*^3/5^H, F-Phe), 115.9 (*C*^1^, (OCH_3_)_2_-Phe), 116.3 (*C*^5^H, Pyr), 118.5 (*C*^4^, Pyrrole), 124.1 (*C*^2^, Pyrrole), 126.7 (d, ^4^*J*_CF_ = 3.5 Hz, *C*^1^, F-Phe), 127.5 (*C*^6^H, (OCH_3_)_2_-Phe), 128.5 (*C*^3^H, Pyrrole), 130.8 (d, ^3^*J*_CF_ = 8.4 Hz, *C*^2/6^H, F-Phe), 134.5 (*C*^5^, Imdz), 142.1 (*C*^2^, Imdz), 143.6 (*C*^4^, Imdz), 147.6 (*C*^6^H, Pyr), 152.7 (*C*^2^, Pyr), 156.7 (*C*^4^OCH_3_), 158.6 (*C*^2^OCH_3_), 159.8 (*C*ONH), 162.0 (d, ^1^*J*_CF_ = 245.6 Hz, *C*F) ppm; MS (ESI, 70 eV) *m*/*z* 544 [MH]^+^; HRMS (EI, 70 eV) *m*/*z* [M]^+^ calcd. for C_29_H_26_FN_5_O_3_S, 543.1740; found, 543.1740.

*4-(2,5-Dimethoxyphenyl)-N-(4-(5-(4-fluorphenyl)-2-(methylthio)-1H-imidazol-4-yl)-pyridin-2-yl)-1-methyl-1H-pyrrole-2-carboxamide* (**16b**). Synthesis was performed according to the procedure described for **16a** starting from **15b** (604 mg, 2.31 mmol), PyBOP (1.45 g, 2.79 mmol), DIPEA (1.20 mL, 6.87 mmol), and **7** (904 mg, 3.01 mmol) in 12 mL anhyd. DMF. The crude product was purified by flash chromatography (SiO_2_, 30%–100% ethyl acetate/petrol ether and RP-18, 50%–100% methanol/H_2_O) to afford **16b** as pale yellowish solid. Yield 255 mg (20%); m.p. 127 °C; ^1^H-NMR (DMSO-*d*_6_): δ = 2.64 (s, 3H, SC*H*_3_), 3.76 (s, 3H, C^5^OC*H*_3_), 3.81 (s, 3H, C^2^OC*H*_3_), 3.92 (s, 3H, C*H*_3_), 6.73 (dd, ^3^*J* = 8.9 Hz, ^4^*J* = 3.1 Hz, 1H, C^4^*H*, (OCH_3_)_2_-Phe), 6.96 (d, ^3^*J* = 9.0 Hz, 1H, C^3^*H*, (OCH_3_)_2_-Phe), 7.06 (dd, ^3^*J* = 5.3 Hz, ^4^*J* = 1.6 Hz, 1H, C^5^*H*, Pyr), 7.17 (d, ^4^*J* = 3.1 Hz, 1H, C^6^*H*, (OCH_3_)_2_-Phe), 7.24–7.30 (m, 2H, C^3/5^*H*, F-Phe), 7.50–7.55 (m, 2H, C^2/6^*H*, F-Phe), 7.56 (d, ^4^*J* = 1.7 Hz, 1H, C^5^*H*, Pyrrole), 7.80 (d, ^4^*J* = 1.8 Hz, 1H, C^3^*H*, Pyrrole), 8.22 (d, ^3^*J* = 5.2 Hz, 1H, C^6^*H*, Pyr), 8.35 (bs, 1H, C^3^*H*, Pyr), 10.15 (bs, 1H, CON*H*), 12.77 (vbs, 1H, N*H*) ppm; ^13^C-NMR (DMSO-*d*_6_): δ = 15.2 (S*C*H_3_), 36.7 (*C*H_3_), 55.4 (C^5^O*C*H_3_), 55.9 (C^2^O*C*H_3_), 111.3 (*C*^4^H, (OCH_3_)_2_-Phe and *C*^3^H, Pyr), 112.5 (*C*^6^H, (OCH_3_)_2_-Phe), 112.8 (*C*^3^H, (OCH_3_)_2_-Phe), 113.6 (*C*^3^H, Pyrrole), 115.7 (d, ^2^*J*_CF_ = 21.5 Hz, *C*^3/5^H, F-Phe), 116.5 (*C*^5^H, Pyr), 118.3 (*C*^4^, Pyrrole), 123.8 (*C*^1^, (OCH_3_)_2_-Phe), 124.3 (*C*^2^, Pyrrole), 129.7 (*C*^5^H, Pyrrole), 130.42 (*C*^2/6^H, F-Phe), 142.5 (*C*^2^, Imdz and *C*^4^, Pyr), 147.7 (*C*^6^H, Pyr), 150.1 (*C*^2^OCH_3_), 152.7 (*C*^1^, Pyr), 153.4 (*C*^5^OCH_3_), 159.8 (*C*ONH), 161.9 (d, ^1^*J*_CF_ = 247.7 Hz, *C*F) ppm; MS (ESI, 70 eV) *m*/*z* 544 [MH]^+^; HRMS (EI, 70 eV) *m*/*z* [M]^+^ calcd. for C_29_H_26_FN_5_O_3_S, 543.1740; found, 543.1740.

#### 3.2.6. Synthesis of Sulfoxides **10j**–**k**, **11f**–**g**, **12h**–**m**, **16c**

The sulfide (1.0 equiv) was dissolved in THF and H_2_O was added (approx. 3:1). The mixture was stirred at 0 °C for 10 min before an ice-cold aq. solution of potassium peroxomonosulfate (Oxone^®^, 0.6 equiv) was added and stirring continued for 0.5–2 h at the same temp. After completion of the reaction sat. aq. NaHCO_3_ solution, H_2_O, and ethyl acetate were added and the phases were separated. The organic layer was washed with H_2_O and sat. aq. NaCl solution, dried over anhyd. Na_2_SO_4_, and the solvent was removed under reduced pressure. Purification of the crude products was achieved by crystallization from ethyl acetate or flash chromatography (SiO_2_ and RP-18, eluent and mixing ratio given for each compound) to afford the appropriate compound.

*N-(2-Ethoxyphenyl)-4-(5-(4-fluorophenyl)-2-(methylsulfinyl)-1H-imidazol-4-yl)pyridin-2-amine* (**10j**). Synthesis was performed according to the general procedure for sulfoxidation starting from **10c** (300 mg, 713 µmol) in 5 mL THF and 2 mL H_2_O. Flash chromatography (RP-18, 20%–90% methanol/H_2_O) afforded **10j** as voluminous yellow solid. Yield 255 mg (82%); C_23_H_21_FN_4_O_2_S (M*_r_* 436.51); m.p. 152 °C; ^1^H-NMR (CDCl_3_): δ = 1.42 (t, ^3^*J =* 7.0 Hz, 3H, C*H*_3_), 3.03 (s, 3H, SC*H*_3_), 4.07 (q, ^3^*J =* 7.0 Hz, 2H, C*H*_2_), 6.94–6.78 (m, 4H, C^4–6^*H*, EtO-Phe and C^5^*H*, Pyr), 7.03 (dd, ^4^*J =* 1.4 Hz, ^5^*J =* 0.7 Hz, 1H, C^3^*H*, Pyr), 7.07–7.13 (m, 3H, C^3/5^*H*, F-Phe and N*H*), 7.46–7.50 (m, 2H, C^2/6^*H*, F-Phe), 7.62 (dd, ^3^*J =* 7.8 Hz, ^4^*J =* 1.5 Hz, 1H, C^3^*H*, EtO-Phe), 8.14 (dd, ^3^*J =* 5.3 Hz, ^5^*J =* 0.7 Hz, 1H, C^6^*H*, Pyr) ppm; ^13^C-NMR (CDCl_3_): δ = 14.9 (*C*H_3_), 40.8 (S*C*H_3_), 64.1 (*C*H_2_), 106.9 (*C*^3^H, Pyr), 111.5 (*C*^3^H, EtO-Phe), 113.3 (*C*^5^H, Pyr), 116.0 (d, ^2^J_CF_ = 21.7 Hz, *C*^3/5^H, F-Phe), 118.2 (*C*^6^H, EtO-Phe), 120.6 (*C*^5^H, EtO-Phe), 121.9 (*C*^4^H, EtO-Phe), 129.9 (*C*^1^, EtO-Phe), 130.6 (d, ^3^*J*_CF_ = 8.2 Hz, *C*^2/6^H, F-Phe), 146.7 (*C*^2^, Imdz), 148.2 (*C*^2^, EtO-Phe), 148.5 (*C*^6^H, Pyr), 156.0 (*C*^2^, Pyr), 162.9 (d, ^1^*J*_CF_ = 248.8 Hz, *C*F) ppm; MS (ESI, 70 eV) *m*/*z* 437 [MH]^+^.

*N-(3,4-Dimethoxyphenethyl)-4-(5-(4-fluorophenyl)-2-(methylsulfinyl)-1H-imidazol-4-yl)-pyridin-2-amine* (**10k**). Synthesis was performed according to the general procedure for sulfoxidation starting from **10d** (500 mg, 1.08 mmol) in 5 mL H_2_O in 9 mL THF and 3 mL H_2_O. Flash chromatography (RP-18, 20%–90% methanol/H_2_O) afforded **10d** as pale yellow solid. Yield 208 mg (39%); C_25_H_25_FN_4_O_3_S (M*_r_* 480.56); m.p. 183 °C; ^1^H-NMR (CDCl_3_): δ = 2.74 (t, ^3^*J =* 6.8 Hz, 1H, C*H*_2_CH_2_NH), 2.97 (s, 3H, SC*H*_3_), 3.36 (m, 2H, CH_2_C*H*_2_NH), 3.76 (s, 6H, 2 OC*H*_3_), 5.00 (bs, 1H, CH_2_CH_2_N*H*), 6.49 (bs, 1H, C^3^*H*, Pyr), 6.52 (dd, ^3^*J =* 5.4 Hz, ^4^*J =* 1.3 Hz, 1H, C^5^*H*, Pyr), 6.60–6.63 (m, 2H, C^2/6^*H*, (OCH_3_)_2_-Phe), 6.71 (d, ^3^*J =* 8.7 Hz, 1H, C^5^*H*, (OCH_3_)_2_-Phe), 6.96–7.02 (m, 2H, C^3/5^*H*, F-Phe), 7.36–7.41 (m, 2H, C^2/6^*H*, F-Phe), 7.80 (d, ^3^*J =* 5.5 Hz, 1H, C^6^*H*, Pyr) ppm; ^13^C-NMR (CDCl_3_): δ = 35.1 (*C*H_2_CH_2_NH), 40.7 (S*C*H_3_), 43.3 (CH_2_*C*H_2_NH), 55.9 (C^3^O*C*H_3_), 55.9 (C^4^O*C*H_3_), 104.6 (*C*^3^H, Pyr), 111.2 (*C*^5^H, Pyr), 111.4 (*C*^5^H, (OCH_3_)_2_-Phe), 112.1 (*C*^2^H, (OCH_3_)_2_-Phe), 115.9 (d, ^2^*J*_CF_ = 21.7 Hz, *C*^3/5^H, F-Phe), 120.7 (*C*^6^H, (OCH_3_)_2_-Phe), 127.2 (*C*^1^, F-Phe), 130.5 (d, ^3^*J*_CF_ = 8.2 Hz, *C*^2/6^H, F-Phe), 131.5 (*C*^1^, (OCH_3_)_2_-Phe), 135.3 (*C*^4/5^, Imdz), 141.6 (*C*^4^, Pyr), 146.8 (*C*^2^, Imdz), 147.4 (*C*^6^H, Pyr), 147.7 (*C*^4^OCH_3_), 149.0 (*C*^3^OCH_3_), 158.6 (*C*^2^, Pyr), 162.9 (d, ^1^*J*_CF_ = 249.2 Hz, *C*F) ppm; MS (ESI, 70 eV) *m*/*z* 481 [MH]^+^.

*3-(2,4-Dimethoxyphenyl)-N-(4-(5-(4-fluorphenyl)-2-(methylsulfinyl)-1H-imidazol-4-yl)-pyridin-2-yl)-propanamide* (**11f**). Synthesis was performed according to the general procedure for sulfoxidation starting from **11a** (100 mg, 203 µmol) in 2 mL THF and 0.6 mL H_2_O. Flash chromatography (SiO_2_, 40%–100% ethyl acetate/petrol ether) afforded **11f** as colorless solid. Yield 96.1 mg (93%); C_26_H_25_FN_4_O_4_S (M*_r_* 508.57); m.p. 209 °C; ^1^H-NMR (DMSO-*d*_6_): δ = 2.58 (t, ^3^*J* = 7.5 Hz, 2H, CH_2_C*H*_2_CO), 2.75 (t, ^3^*J* = 7.4 Hz, 2H, C*H*_2_CH_2_CO), 3.08 (s, 3H, SC*H*_3_), 3.72 (s, 3H, C^2^OC*H*_3_), 3.77 (s, 3H, C^4^OC*H*_3_), 6.42 (dd, ^3^*J* = 8.3 Hz, ^4^*J* = 2.4 Hz, 1H, C^5^*H*, (OCH_3_)_2_-Phe), 6.51 (d, ^4^*J* = 2.4 Hz, 1H, C^3^*H*, (OCH_3_)_2_-Phe), 7.01–7.05 (m, 2H, C^5^*H*, Pyr and C^6^*H*, (OCH_3_)_2_-Phe), 7.26–7.32 (m, 2H, C^3/5^*H*, F-Phe), 7.51–7.55 (m, 2H, C^2/6^*H*, F-Phe), 8.19 (d, ^3^*J* = 5.2 Hz, 1H, C^6^*H*, Pyr), 8.34 (bs, 1H, C^3^*H*, Pyr), 10.40 (s, 1H, CON*H*), 13.89 (bs, N*H*) ppm; ^13^C-NMR (DMSO-*d*_6_): δ = 24.7 (*C*H_2_CH_2_CO), 36.3 (CH_2_*C*H_2_CO), 39.1 (S*C*H_3_), 55.1 (C^2^O*C*H_3_), 55.3 (C^4^O*C*H_3_), 98.3 (*C*^3^H, (OCH_3_)_2_-Phe), 104.3 (*C*^5^H, (OCH_3_)_2_-Phe), 111.1 (*C*^3^H, Pyr), 115.8 (d, ^2^*J*_CF_ = 21.7 Hz, *C*^3/5^H, F-Phe), 117.0 (*C*^5^H, Pyr), 120.9 (*C*^1^, (OCH_3_)_2_-Phe), 127.3 (*C*^1^, F-Phe), 129.8 (*C*^6^H, (OCH_3_)_2_-Phe), 130.8 (d, ^3^*J*_CF_ = 8.2 Hz, *C*^2/6^H, F-Phe), 133.4 (*C*^4^, Imdz), 133.9 (*C*^5^, Imdz), 142.2 (*C*^4^, Pyr), 147.9 (*C*^6^H, Pyr), 148.9 (*C*^2^, Imdz), 152.6 (*C*^2^, Pyr), 157.9 (*C*^4^OCH_3_), 159.0 (*C*^2^OCH_3_), 162.1 (d, ^1^*J*_CF_ = 245.5 Hz, *C*F), 171.54 (*C*O) ppm; MS (ESI, 70 eV) *m*/*z* 509 [MH]^+^; HRMS (EI, 70 eV) *m*/*z* [M]^+^ calcd. for C_26_H_25_FN_4_O_4_S, 508.1581; found, 508.1581.

*3-(2,5-Dimethoxyphenyl)-N-(4-(5-(4-fluorophenyl)-2-(methylsulfinyl)-1H-imidazol-4-yl)-pyridin-2-yl)propanamide* (**11g**). Synthesis was performed according to the general procedure for sulfoxidation starting from **11b** (100 mg, 203 µmol) in 2 mL THF and 0.6 mL H_2_O. Crystallization from ethyl acetate afforded **11g** as colorless solid. Yield 26.6 mg (26%); m.p. 204 °C; ^1^H-NMR (DMSO-*d*_6_): δ = 2.62 (t, ^3^*J* = 7.7 Hz, 2H, CH_2_C*H*_2_CO), 2.80 (t, ^3^*J* = 7.5 Hz, 2H, C*H*_2_CH_2_CO), 3.09 (s, 3H, SC*H*_3_), 3.66 (s, 3H, C^5^OC*H*_3_), 3.72 (s, 3H, C^2^OC*H*_3_), 6.72 (dd, ^3^*J* = 8.8 Hz, ^4^*J* = 3.1 Hz, 1H, C^4^*H*, (OCH_3_)_2_-Phe), 6.77 (d, ^4^*J* = 3.0 Hz, 1H, C^6^*H*, (OCH_3_)_2_-Phe), 6.86 (d, ^3^*J* = 8.8 Hz, 1H, C^3^*H*, (OCH_3_)_2_-Phe), 7.02 (dd, ^3^*J* = 5.2 Hz, ^4^*J* = 1.5 Hz, 1H, C^5^*H*, Pyr), 7.25–7.31 (m, 2H, C^3/5^*H*, F-Phe), 7.51–7.55 (m, 2H, C^2/6^*H*, F-Phe), 8.19 (d, ^3^*J* = 5.0 Hz, 1H, C^6^*H*, Pyr), 8.33 (s, 1H, C^3^*H*, Pyr), 10.45 (s, 1H, CON*H*), 14.00 (bs, 1H, N*H*) ppm; ^13^C-NMR (DMSO-*d*_6_): δ = 25.3 (*C*H_2_CH_2_CO), 36.0 (CH_2_*C*H_2_CO), 39.0 (S*C*H_3_), 55.2 (C^5^O*C*H_3_), 55.8 (C^2^O*C*H_3_), 111.2 (*C*^4^H, (OCH_3_)_2_-Phe), 111.3 (*C*^3^H, Pyr), 111.5 (*C*^3^H, (OCH_3_)_2_-Phe), 115.8 (d, ^2^*J*_CF_ = 21.3 Hz, *C*^3/5^H, F-Phe), 115.9 (*C*^6^H, (OCH_3_)_2_-Phe), 126.5 (*C*^1^, F-Phe), 130.8 (d, ^3^*J*_CF_ = 8.3 Hz, *C*^2/6^H, F-Phe), 142.1 (*C*^4^, Pyr), 148.0 (*C*^6^H, Pyr), 148.8 (*C*^2^, Imdz), 151.2 (*C*^2^OCH_3_), 152.58 (*C*^2^, Pyr), 153.0 (*C*^5^OCH_3_), 162.1 (d, ^1^*J*_CF_ = 245.6 Hz, *C*F), 171.42 (*C*O) ppm; MS (ESI, 70 eV) *m/z* 509 [MH]^+^.

*1-(2,4-Dimethoxyphenyl)-3-(4-(5-(4-fluorophenyl)-2-(methylsulfinyl)-1H-imidazol-4-yl)-pyridin-2-yl)-carbamide* (**12h**). Synthesis was performed according to the general procedure for sulfoxidation starting from **12a** (85.0 mg, 177 µmol) in 3.4 mL THF and 1 mL H_2_O. Flash chromatography (SiO_2_, 35%–100% ethyl acetate/petrol ether and RP-18, 55%–100% methanol/H_2_O) afforded **12h** as colorless solid. Yield 18.9 mg (22%); C_24_H_22_FN_5_O_4_S (M*_r_* 495.53); m.p. 227 °C; ^1^H-NMR (DMSO-*d*_6_): δ = 3.08 (s, 3H, SC*H*_3_), 3.74 (s, 3H, C^4^OC*H*_3_), 3.88 (s, 3H, C^2^OC*H*_3_), 6.48 (dd, ^3^*J* = 8.9 Hz, ^4^*J* = 2.7 Hz, 1H, C^5^*H*, (OCH_3_)_2_-Phe), 6.62 (d, ^4^*J* = 2.7 Hz, 1H, C^3^*H*, (OCH_3_)_2_-Phe), 6.95 (dd, ^3^*J* = 5.4 Hz, ^4^*J* = 1.5 Hz, 1H, C^5^*H*, Pyr), 7.27–7.33 (m, 2H, C^3/5^*H*, F-Phe), 7.46 (bs, 1H, C^3^*H*, Pyr), 7.51–7.56 (m, 2H, C^2/6^*H*, F-Phe), 8.02 (d, ^3^*J* = 8.9 Hz, 1H, C^6^*H*, (OCH_3_)_2_-Phe), 8.19 (d, ^3^*J* = 5.3 Hz, 1H, C^6^*H*, Pyr), 9.70 (s, 1H, N*H*), 11.07 (bs, 1H, N*H*), 13.87 (bs, 1H, N*H*, Imdz) ppm; ^13^C-NMR (DMSO-*d*_6_): δ = 39.1 (S*C*H_3_), 55.3 (C^2^O*C*H_3_), 56.0 (C^4^O*C*H_3_), 98.8 (*C*^3^H, (OCH_3_)_2_-Phe), 104.1 (*C*^5^H, (OCH_3_)_2_-Phe), 109.2 (*C*^3^H, Pyr), 115.1 (*C*^5^H, Pyr), 115.8 (d, ^2^*J*_CF_ = 21.7 Hz, *C*^3/5^H, F-Phe), 119.8 (*C*^6^H, (OCH_3_)_2_-Phe), 121.8 (*C*^1^, (OCH_3_)_2_-Phe), 127.2 (*C*^1^, F-Phe), 130.8 (d, ^3^*J*_CF_ = 8.4 Hz, *C*^2/6^H, F-Phe), 132.7 (*C*^4^, Imdz), 134.1 (*C*^5^, Imdz), 142.8 (*C*^4^, Pyr), 146.5 (*C*^6^H, Pyr), 149.1 (*C*^2^, Imdz), 149.4 (*C*^2^OCH_3_), 152.2 (*C*O), 153.6 (*C*^2^, Pyr), 155.2 (*C*^4^OCH_3_), 162.14 (d, ^1^*J*_CF_ = 244.6 Hz, *C*F) ppm; MS (ESI, 70 eV) *m*/*z* 496 [MH]^+^.

*1-(4-(5-(4-Fluorophenyl)-2-(methylsulfinyl)-1H-imidazol-4-yl)pyridin-2-yl)-3-(4-meth-oxyphenyl)carbamide* (**12i**). Synthesis was performed according to the general procedure for sulfoxidation starting from **12c** (100 mg, 225 µmol) in 2 mL THF and 0.6 mL H_2_O. Flash chromatography (RP-18, 50%–70% methanol/H_2_O) afforded **12i** as colorless solid. Yield 62.6 mg (61%); C_23_H_20_FN_5_O_3_S (M*_r_* 465.50); m.p. 233 °C; ^1^H-NMR (DMSO-*d*_6_): δ = 3.09 (s, 3H, SC*H*_3_), 3.73 (s, 3H, C^4^OC*H*_3_), 6.89 (d, ^3^*J* = 9.1 Hz, 2H, C^3/5^*H*, H_3_CO-Phe), 6.95 (dd, ^3^*J* = 5.4 Hz, ^4^*J* = 1.5 Hz, 1H, C^5^*H*, Pyr), 7.28–7.34 (m, 2H, C^3/5^*H*, F-Phe), 7.41 (d, ^3^*J* = 9.1 Hz, 2H, C^2/6^*H*, H_3_CO-Phe), 7.51–7.56 (m, 2H, C^2/6^*H*, F-Phe), 7.65 (bs, 1H, C^3^*H*, Pyr), 8.18 (d, ^3^*J* = 5.4 Hz, 1H, C^6^*H*, Pyr), 9.43 (s, 1H, N*H*), 10.47 (bs, 1H, N*H*), 13.92 (bs, 1H, N*H*, Imdz) ppm; ^13^C-NMR (DMSO-*d*_6_): δ = 39.1 (S*C*H_3_), 55.2 (C^4^O*C*H_3_), 109.4 (*C*^3^H, Pyr), 114.0 (*C*^3/5^H, H_3_CO-Phe), 115.2 (*C*^5^H, Pyr), 115.9 (d, ^2^*J*_CF_ = 21.7 Hz, *C*^3/5^H, F-Phe), 120.6 (*C*^2/6^H, H_3_CO-Phe), 127.5 (*C*^1^, F-Phe), 130.9 (d, ^3^*J*_CF_ = 8.7 Hz, *C*^2/6^H, F-Phe), 132.0 (*C*^1^, H_3_CO-Phe), 146.9 (*C*^6^H, Pyr), 148.8 (*C*^2^, Imdz), 152.2 (*C*O), 153.5 (*C*^2^, Pyr), 154.9 (*C*^4^OCH_3_), 162.2 (d, ^1^*J*_CF_ = 246.7 Hz, *C*F) ppm; MS (ESI, 70 eV) *m*/*z* 466 [MH]^+^.

*1-(4-(5-(4-Fluorophenyl)-2-(methylsulfinyl)-1H-imidazol-4-yl)pyridin-2-yl)-3-(m-tolyl)-carbamide* (**12j**). Synthesis was performed according to the general procedure for sulfoxidation starting from **12d** (100 mg, 231 µmol) in 2 mL THF and 0.6 mL H_2_O. Flash chromatography (SiO_2_, 20%–100% ethyl acetate/petrol ether) afforded **12j** as colorless solid. Yield 74.8 mg (72%); C_23_H_20_FN_5_O_2_S (M*_r_* 449.50); m.p. 235 °C; ^1^H-NMR (DMSO-*d*_6_): δ = 2.29 (s, 3H, C*H_3_*), 3.09 (s, 3H, SC*H*_3_), 6.83 (d, ^3^*J* = 7.4 Hz, 1H, C^4^*H*, Tol), 6.97 (dd, ^3^*J* = 5.4 Hz, ^4^*J* = 1.4 Hz, 1H, C^5^*H*, Pyr), 7.18 (t, ^3^*J* = 7.7 Hz, 1H, C^5^*H*, Tol), 7.29–7.35 (m, 4H, C^3/5^*H*, F-Phe and C^2/6^*H*, Tol), 7.52–7.57 (m, 2H, C^2/6^*H*, F-Phe), 7.69 (s, 1H, C^3^*H*, Pyr), 8.19 (d, ^3^*J* = 5.1 Hz, 1H, C^6^*H*, Pyr), 9.48 (s, 1H, N*H*), 10.54 (bs, 1H, N*H*), 13.92 (bs, 1H, N*H*, Imdz) ppm; ^13^C-NMR (75 MHz, DMSO-*d*_6_): δ = 21.2 (*C*H_3_), 39.1 (S*C*H_3_), 109.3 (*C*^3^H, Pyr), 115.3 (*C*^5^H, Pyr), 115.9 (d, ^2^*J*_CF_ = 21.5 Hz, *C*^3/5^H, F-Phe), 116.0 (*C*^6^H, Tol), 119.3 (*C*^2^H, Tol), 123.2 (*C*^4^H, Tol), 128.7 (*C*^5^H, Tol), 130.9 (*C*^2/6^H, F-Phe), 138.1 (*C*^3^, Tol), 138.9 (*C*^1^, Tol), 143.1 (*C*^4^, Pyr), 147.0 (*C*^6^H, Pyr), 148.7 (*C*^2^, Imdz), 152.1 (*C*O), 153.4 (*C*^2^, Pyr), 162.2 (d, ^1^*J*_CF_ = 244.0 Hz, *C*F) ppm; MS (ESI, 70 eV) *m*/*z* 450 [MH]^+^.

*1-(3-Chloro-4-methylphenyl)-3-(4-(5-(4-fluorophenyl)-2-(methylsulfinyl)-1H-imidazol-4-yl)pyridin-2-yl)-carbamide* (**12k**). Synthesis was performed according to the general procedure for sulfoxidation starting from **12e** (100 mg, 214 µmol) in 4 mL THF and 1 mL H_2_O. Flash chromatography (SiO_2_, 20%–100% ethyl acetate/petrol ether and RP-18, 50%–100% methanol/H_2_O) afforded **12k** as beige solid. Yield 78.0 mg (75%); C_23_H_19_ClFN_5_O_2_S (M*_r_* 483.95); ^1^H-NMR (DMSO-*d*_6_): δ = 2.27 (s, 3H, C*H*_3_), 3.09 (s, 3H, SC*H*_3_), 6.99 (dd, ^3^*J* = 5.4 Hz, ^4^*J* = 1.3 Hz, 1H, C^5^*H*, Pyr), 7.26 (s, 1H, C^5^*H*, Cl-Tol), 7.26 (s, 1H, C^6^*H*, Cl-Tol), 7.29–7.35 (m, 2H, C^3/5^*H*, F-Phe), 7.52–7.56 (m, 2H, C^2/6^*H*, F-Phe), 7.65 (bs, 1H, C^3^*H*, Pyr), 7.76 (bs, 1H, C^2^*H*, Cl-Tol), 9.55 (s, 1H, N*H*), 10.77 (bs, 1H, N*H*), 13.93 (bs, 1H, N*H*, Imdz) ppm; ^13^C-NMR (DMSO-*d*_6_): δ = 18.8 (*C*H_3_), 39.1 (S*C*H_3_), 109.2 (*C*^3^H, Pyr), 115.4 (*C*^5^H, Pyr), 115.9 (d, ^2^*J*_CF_ = 21.5 Hz, *C*^3/5^H, F-Phe), 117.5 (*C*^6^H, Cl-Tol), 118.7 (*C*^2^H, Cl-Tol), 129.0 (*C*^4^, Cl-Tol), 131.1 (*C*^2/6^H, F-Phe), 131.2 (*C*^5^, Imdz and *C*^5^H, Cl-Tol), 133.2 (*C*^3^, Cl-Tol), 134.6 (*C*^4^, Imdz), 138.2 (*C*^1^, Cl-Tol), 147.0 (*C*^6^H, Pyr), 148.7 (*C*^2^, Imdz), 152.1 (*C*O), 153.2 (*C*^2^, Pyr), 162.3 (d, ^1^*J*_CF_ = 249.9 Hz, *C*F) ppm; MS (ESI, 70 eV) *m*/*z* 484 [MH]^+^.

*1-(4-(5-(4-Fluorophenyl)-2-(methylsulfinyl)-1H-imidazol-4-yl)pyridin-2-yl)-3-(3-(trifluormethyl)phenyl)carbamide* (**12l**). Synthesis was performed according to the general procedure for sulfoxidation starting from **12f** (20.0 mg, 41.0 µmol) in 0.4 mL THF and 0.1 mL water. Flash chromatography (SiO_2_, 40%–100% ethyl acetate/petrol ether) afforded **12l** as a colorless solid. Yield 15.1 mg (73%); m.p. 235 °C; ^1^H-NMR (DMSO-*d*_6_): δ = 3.10 (s, 3H, SC*H*_3_), 7.02 (dd, ^3^*J* = 5.4 Hz, ^4^*J* = 1.4 Hz, 1H, C^5^*H*, Pyr), 7.30–7.37 (m, 2H, C^3/5^*H*, F-Phe), 7.36 (d, ^3^*J* = 7.7 Hz, 1H, C^4^*H*, F_3_C-Phe), 7.51–7.57 (m, 3H, C^2/6^*H*, F-Phe and C^5^*H*, F_3_C-Phe), 7.65 (d, ^3^*J* = 8.3 Hz, 1H, C^6^*H*, F_3_C-Phe), 7.71 (s, 1H, C^2^*H*, F_3_C-Phe), 8.08 (bs, 1H, C^3^*H*, Pyr), 8.22 (bs, 1H, C^6^*H*, Pyr), 9.59 (s, 1H, N*H*), 10.90 (bs, 1H, N*H*), 13.93 (bs, 1H, N*H*, Imdz) ppm; ^13^C-NMR (DMSO-*d*_6_): δ = 39.1 (S*C*H_3_), 109.3 (*C*^2^H, F_3_C-Phe), 114.7 (q, ^3^*J*_CF_ = 2.5 Hz, *C*^3^H, Pyr), 115.5 (*C*^5^H, Pyr), 115.9 (d, ^3^*J*_CF_ = 21.9 Hz, *C*^3/5^H, F-Phe), 118.7 (q, ^3^*J*_CF_ = 2.1 Hz, *C*^4^H, F_3_C-Phe), 122.5 (*C*^6^H, F_3_C-Phe), 124.2 (q, ^1^*J*_CF_ = 272.2 Hz, *C*F_3_), 125.9 (d, ^4^*J*_CF_ = 3.2 Hz, *C*^1^, F-Phe), 129.6 (q, ^2^*J*_CF_ = 31.4 Hz, *C*^3^, F_3_C-Phe), 130.0 (*C*^5^H, F_3_C-Phe), 130.8 (*C*^5^, Imdz), 131.1 (d, ^4^*J*_CF_ = 11.4 Hz, *C*^2/6^H, F-Phe), 134.5 (*C*^4^, Imdz), 139.9 (*C*^1^, F_3_C-Phe), 143.4 (*C*^4^, Pyr), 147.1 (*C*^6^H, Pyr), 148.7 (*C*^2^, Imdz), 152.2 (*C*O), 162.4 (d, ^1^*J*_CF_ = 242.12 Hz, *C*F) ppm; MS (ESI, 70 eV) *m*/*z* 504 [MH]^+^.

*1-(4-(5-(4-Fluorophenyl)-2-(methylthio)-1H-imidazol-4-yl)pyridin-2-yl)-3-(naphthalen-1-yl)carbamide* (**12m**). Synthesis was performed according to the general procedure for sulfoxidation starting from **12g** (51.0 mg, 109 µmol) in 5 mL THF and 1.5 mL H_2_O. Flash chromatography (SiO_2_, 20%–100% ethyl acetate/petrol ether) afforded **12m** as colorless solid. Yield 37.0 mg (70%); C_26_H_20_FN_5_O_2_S (M*_r_* 485.54); m.p. 244 °C; ^1^H-NMR (DMSO-*d*_6_): δ = 3.10 (s, 3H, SC*H*_3_), 7.04 (dd, ^3^*J* = 5.4 Hz, ^4^*J* = 1.4 Hz, 1H, C^5^*H*, Pyr), 7.31–7.36 (m, 2H, C^3/5^*H*, F-Phe), 7.49 (t, ^3^*J* = 7.9 Hz, 1H, C^3^*H*, Naph), 7.54–7.59 (m, 4H, C^3^*H*, Pyr and C^2/6^*H*, F-Phe and C^6^*H*, Naph), 7.63–7.68 (m, 2H, C^4/7^*H*, Naph), 7.96 (dd, ^3^*J* = 8.1 Hz, ^4^*J* = 0.8 Hz, 1H, C^5^*H*, Naph), 8.16–8.20 (m, 2H, C^2/8^*H*, Naph), 8.34 (d, ^3^*J* = 5.4 Hz, 1H, C^6^*H*, Pyr), 8.93 (s, 1H, N*H*), 11.58 (bs, 1H, N*H*), 13.95 (bs, 1H, N*H*, Imdz) ppm; ^13^C-NMR (DMSO-*d*_6_): δ = 39.1 (S*C*H_3_), 109.4 (*C*^3^H, Pyr), 115.3 (*C*^5^H, Pyr), 115.9 (d, ^2^*J*_CF_ = 21.8 Hz, *C*^3/5^H, F-Phe), 116.9 (*C*^2^H, Naph), 120.9 (*C*^8^H, Naph), 123.1 (*C*^4^H, Naph), 125.5 (*C*^1^, Naph), 126.0 (*C*^6^H, Naph), 126.0 (*C*^3^H, Naph), 126.3 (*C*^7^H, Naph), 128.6 (*C*^5^H, Naph), 130.7 (*C*^5^, Imdz), 130.9 (*C*^2/6^H, F-Phe), 133.7 (*C*^4a^, Naph), 134.1 (*C*^8a^, Naph), 143.5 (*C*^4^, Pyr), 146.7 (*C*^6^H, Pyr), 148.9 (*C*^2^, Imdz), 152.6 (*C*O), 153.6 (*C*^2^, Pyr), 162.3 (d, ^1^*J*_CF_ = 246.9 Hz, *C*F) ppm; MS (ESI, 70 eV) *m*/*z* 486 [MH]^+^.

*4-(2,4-Dimethoxyphenyl)-N-(4-(5-(4-fluorophenyl)-2-(methylsulfinyl)-1H-imidazol-4-yl)-pyridin-2-yl)-1-methyl-1H-pyrrol-2-carboxamide* (**16c**). Synthesis was performed according to the general procedure for sulfoxidation starting from **16a** (196 mg, 368 µmol) in 4 mL THF and 1 mL H_2_O. Flash chromatography (SiO_2_, 40%–100% ethyl acetate/petrol ether) afforded **16c** as yellow solid. Yield 182 mg (88%); C_29_H_26_FN_5_O_4_S (M*_r_* 559.62); m.p. 136 °C; ^1^H-NMR (DMSO-*d*_6_): δ = 3.11 (s, 3H, SC*H*_3_), 3.78 (s, 3H, C^2^OC*H*_3_), 3.86 (s, 3H, C^4^OC*H*_3_), 3.90 (s, 3H, C*H*_3_), 6.57 (dd, ^3^*J* = 8.5 Hz, ^4^*J* = 2.4 Hz, 1H, C^5^*H*, (OCH_3_)_2_-Phe), 6.61 (d, ^4^*J* = 2.3 Hz, 1H, C^3^*H*, (OCH_3_)_2_-Phe), 7.07 (d, ^3^*J* = 4.8 Hz, 1H, C^5^*H*, Pyr), 7.23–7.38 (m, 2H, C^3/5^*H*, F-Phe), 7.42 (bs, 1H, C^3^*H*, Pyrrole), 7.46 (d, ^3^*J* = 8.4 Hz, 1H, C^6^*H*, (OCH_3_)_2_-Phe), 7.54–7.59 (m, 2H, C^2/6^*H*, F-Phe), 7.68 (bs, 1H, C^5^*H*, Pyrrole), 8.22 (d, ^3^*J* = 4.5 Hz, 1H, C^6^*H*, Pyr), 8.42 (s, 1H, C^3^*H*, Pyr), 10.17 (s, 1H, CON*H*), 13.89 (s, 1H, N*H*) ppm; ^13^C-NMR (DMSO-*d*_6_): δ = 36.6 (*C*H_3_), 39.2 (S*C*H_3_), 55.2 (C^2^O*C*H_3_), 55.4 (C^4^O*C*H_3_), 98.8 (*C*^3^H, (OCH_3_)_2_-Phe), 105.2 (*C*^5^H, (OCH_3_)_2_-Phe), 111.8 (*C*^3^H, Pyr), 113.3 (*C*^5^H, Pyrrole), 115.9 (*C*^1^, (OCH_3_)_2_-Phe), 116.0 (d, ^2^*J*_CF_ = 22.4 Hz, *C*^3/5^H, F-Phe), 116.1 (*C*^5^H, Pyr), 118.5 (*C*^4^, Pyrrole), 124.0 (*C*^2^, Pyrrole), 125.8 (d, ^4^*J*_CF_ = 3.3 Hz, *C*^1^, F-Phe), 127.5 (*C*^6^H, (OCH_3_)_2_-Phe), 128.6 (*C*^3^H, Pyrrole), 131.3 (d, ^3^*J*_CF_ = 8.3 Hz, *C*^2/6^H. F-Phe), 131.9 (*C*^4^, Imdz), 135.1 (*C*^5^, Imdz), 143.0 (*C*^4^, Pyr), 147.8 (*C*^6^H, Pyr), 148.5 (*C*^2^, Imdz), 152.9 (*C*^2^, Pyr), 156.7 (*C*^4^OCH_3_), 158.6 (*C*^2^OCH_3_), 159.9 (*C*O), 162.4 (d, ^1^*J*_CF_ = 244.3 Hz, *C*F) ppm; MS (ESI, 70 eV) *m*/*z* 560 [MH]^+^.

### 3.3. Kinase Assays and IC_50_ Determination

In vitro kinase assays using 2 μCi 32P-γ-ATP per reaction as co-factor were carried out in kinase buffer containing 25 mM Tris-HCl [pH 7.5], 10 mM MgCl_2_, 100 µM EDTA, and 10 µM ATP. Potential inhibitor compounds were used in a dilution series ranging from 10 μM to 5 nM final reaction concentration which was prepared by serial dilution in DMSO. Recombinant human CK1δ transcription variant 1 (expressed and purified as GST fusion protein as described earlier [60]) and human GST-CK1ε (Invitrogen) were used as sources of enzyme while α-casein (C6780; Sigma-Aldrich) was used as substrate. Kinase reactions were incubated for 30 min at 30 °C. Subsequently, reactions were separated by SDS-PAGE and phosphorylated protein bands were visualized on dried gels by autoradiography. The phosphorylated substrate protein bands were excised and phosphorylation was quantified by Cherenkov counting. Dose-response analyses and calculation of IC_50_ values were carried out using GraphPad Prism 6 statistical software (San Diego, CA, USA).

### 3.4. Cell Culture

#### 3.4.1. Cell Lines

The human pancreatic cancer cell lines Colo357 [61], Panc-1 [62], and MiaPaCa-2 [63] were grown in Dulbecco’s modified Eagle’s medium (DMEM). Panc89 [64] was grown in DMEM:RPMI-1640 medium in ratio 1:1. The human colon adenocarcinoma cell line HT-29 [65] was grown in McCoy’s 5A medium. All media were supplemented with 10% fetal calf serum (FCS), 100 units·mL^−1^ penicillin, 100 µg∙mL^−1^ streptomycin and 2 mM glutamine. All cells were grown at 37 °C in a humidified 5% carbon dioxide atmosphere.

#### 3.4.2. Cell Assays and EC_50_ Determination

In order to determine the cytotoxic effects of investigated inhibitors conventional MTT assay were used. 1 × 10^4^ cells∙well^−1^ were seeded in 96-well cell culture plates and cultivated for 24 h. Cell lines were exposed to increasing concentrations of the indicated inhibitor compounds, with untreated and DMSO-treated cells serving as control. After an incubation period of 48 h 10 µL of MTT solution (5 mg∙mL^−1^ in PBS) were added and cells were incubated for 4 h. MTT-containing medium was carefully removed and 100 µL acidic isopropanol (0.04 N HCl in isopropanol) per well were added. For dissolution of formazan crystals plates were shaken for 30 min on an orbital shaker in the dark. Finally, dissolved crystals were measured spectrophotometrically at 570 nM. All experiments were performed in triplicate with four technical replicates per assay. Results were normalized considering the mean optical density value of control wells as 100%. GraphPad Prism 6 (La Jolla, CA, USA) software was used to calculate EC_50_ values.

### 3.5. X-ray Crystallography

#### 3.5.1. Protein Expression, Purification, and Crystallisation of CK1δ

BL21 (DE3) TaKaRa 2 cells (Clontech) were transformed with the plasmid pET28a-tCK1δ which contains a codon-optimized construct of CK1δ spanning residues 1–294 in a pET28a vector (NdeI and XhoI restriction sites) and streaked out on LB agar plates containing 50 μg∙mL^−1^ kanamycin and 20 µg∙mL^−1^ chloramphenicol as selective antibiotics. A pre-culture was prepared by inoculating cells from a single colony in LB medium supplemented with selective antibiotics and 4 mg∙mL^−1^ l-arabinose and overnight cultivation at 37 °C. Expression cultures were prepared by diluting a fresh pre-culture with LB medium (plus selective antibiotics and 4 mg∙mL^−1^ l-arabinose) to an optical density of 0.1 at 600 nm (OD600). Expression cultures were cultivated at 37 °C until an OD600 of 0.6 was reached, then cultivation temperature was reduced to 20 °C and tCK1δ expression was induced by adding 0.5 mM IPTG (isopropyl-β-d-1-thiogalactopyranoside). 16 h after IPTG addition, cells were harvested by centrifugation (4000× *g*, 20 min 4 °C), washed with TBS (20 mM Tris pH 7.5, 300 mM NaCl), and stored at −80 °C until purification.

Cells were thawed and resuspended (4 mL∙g^−1^ wet cell pellet) in TBS plus 0.5 mM TCEP and subsequently lysed on ice by sonication (Vibra-Cell VCX500, Sonics, Newtown, CT, USA). The resultant lysate was clarified by ultracentrifugation (165,000× *g* at 4 °C for 30 min) and supplemented with 5 mM imidazole pH 7.5 before loading it on 2 mL TALON^®^ (Clontech Takara Bio Europe SAS, Saint-Germain-en-Laye, France) resin. After a wash step with TBS containing 10 mM imidazole, tCK1δ was eluted with TBS and 120 mM imidazole, concentrated, and applied to a Superdex S200 16/600 size exclusion chromatography column using TBS as chromatography buffer. Fractions containing monomeric tCK1δ were pooled and concentrated to 10 mg/mL and stored at −80 °C.

For co-crystallization of tCK1δ with **16b**, protein stock solution (10 mg∙mL^−1^) was mixed 30:1 with 10 mM **16b** (solubilized in DMSO) and incubated for 30 min at room temperature. Sitting drop crystallization trials were set up at 4 °C with drop ratios of 3 µL protein/inhibitor solution to 2 µL precipitant solution. Crystals appeared after three to four days in drops containing 0.1 M Hepes pH 7.0, 0.7 M NaH_2_PO_4_, and 0.7 M KH_2_PO_4_. For data collection, these crystals were cryo-protected by swiping them though reservoir solution supplemented with sucrose (60% saturation) and 0.3 mM **16b** and subsequently flash frozen.

Diffraction data was collected at beamLine X06DA at the Swiss Light Source, Paul-Scherrer-Institute, Villigen, Switzerland and processed using XDS [66]. The structure was solved by molecular replacement using the program PHASER [67] with a truncated crystal structure of CK1δ (pdb 4TWC [25]) as search model. Between iterative cycles of refinement using phenix.refine [68] missing loops as well as **16b** were manually built with Coot [69]. Restrains of **16b** were calculated using phenix.elbow [70].

#### 3.5.2. Protein Expression, Purification, and Crystallization of p38α MAPK

The expression and purification of inactive, non-phosphorylated p38α wt MAPK was done as previously reported [71]. Briefly, an N-terminal His_6_-p38α wt construct was transformed into *E. coli* BL21 (DE3) and expressed overnight at 18 °C. The protein was purified by Ni^2+^-NTA-affinity chromatography, followed by anion exchange and size exclusion chromatography after removal of the His-tag by proteolytic cleavage. The pure protein was subsequently concentrated to 10–30 mg·mL^−1^, aliquoted, flash frozen in liquid N_2_, and stored at −80 °C.

**11b** was co-crystallized with p38α wt using conditions similar to those as described previously [72]. Briefly, protein-ligand complexes were prepared by mixing 20 µL p38α wt (10 mg·mL^−1^,) with 0.2 µl compound (50 mM in DMSO) that were subsequently incubated for 60 min on ice. The samples were centrifuged at 13,000 rpm for 10 min to remove excess ligand. Crystals were grown in 24-well crystallisation plates (EasyXtal Tool, Qiagen, Hilden, Germany) using the hanging drop vapor diffusion method and by mixing 1.5 µL protein-ligand solution with 0.5 µL reservoir (100 mM MES pH 5.6–6.2, 20%–30% PEG4000 and 50 mM β-gluco-d-pyranoside). The crystals were protected using 25% PEG400 before they were flash frozen in liquid N_2_. Diffraction data of the p38α-ligand complexes were collected at the PX II beam line of the Swiss Light Source (Paul-Scherrer-Institute, Villigen, Switzerland) using wavelengths close to 1 Å. The datasets were integrated with XDS and scaled using XSCALE [73]. The complex structures were solved by molecular replacement with PHASER [67]. using the published p38α structure (pdb 4DLI [74]) as template. Molecules in the asymmetric unit were manually modified using the program COOT [75]. The final refinement was performed with REFMAC [76]. Inhibitor topology files were generated using the Dundee PRODRG server [77]. Refined structures were validated by Ramachandran plot analysis with RAMPAGE [78]. Data collection, structure refinement statistics, and the Ramachandran plot results are shown in Supplementary Table S6.

## 4. Conclusions

Deriving from hit compounds **1** and **2** we report on design and synthesis of novel sets of highly potent and specific ATP-competitive inhibitors of CK1δ thereby confirming modeled binding modes by X-ray analysis in CK1δ and also in comparison to p38α. Especially **11b** (CK1δ IC_50_ ≤ 3–4 nM, CK1ε IC_50_ = 25 nM, p38α IC_50_ = 10 nM) has been identified as promising agent with IC_50_ values in the single-digit nanomolar range and is therefore among the most potent inhibitors of CK1δ reported so far. Interestingly, X-ray analysis of ligand-protein complexes demonstrated a binding mode for **11b** deviating from typical type I within the active site of p38α, thereby stabilizing the kinase in an intermediate conformation between DFG-in and DFG-out (*DFG-in-between*). CK1δ, however, is addressed by conventional type I inhibition in a DFG-in conformation as confirmed by co-crystallization with **16b**. In addition, at 100 nM **11b** is rather selective for CK1δ/ε hitting only six off-targets from a panel of 321 protein kinases, among them p38α. Compound **11b** further exhibited single-digit micromolar efficacy in MTT viability assays in pancreatic Colo357 and Panc89 carcinoma cell lines. Protein kinase CK1δ, however, executes diverse physiological and pathophysiological functions and specific inhibitors might therefore strongly depend on tissue and cellular background [2,19,28,79]. Consequently, additional experiments using cellular systems as well as optimization of physicochemical properties might prove beneficial. Nevertheless, the implied hit structure has to be considered inept in order to achieve complete CK1 isoform selectivity regarding CK1δ and CK1ε, although moderate discrimination of CK1α and significant selectivity against CK1γ1-3 have been successfully achieved. Based on the current results, additional optimization cycles might therefore focus on decreased fitting into the active site of p38α. Reducing the acrylamide moiety of **1** proved beneficial as the obtained propionic amide **11b** does not show a *Michael* acceptor moiety. All synthesized inhibitors were stable in DMSO solution at room temperature over a period of 72 h. In fact, the compound is among the most potent and stable inhibitors of CK1δ published to date with good selectivity regarding a screen comprising 321 protein kinases and suitable efficacy in different human cancer cell lines. Fortunately, sulfoxidation, which is believed to be the predominant metabolic pathway of this inhibitor class [44], did not abrogate activity. Co-crystallization of compounds with CK1δ and related p38α confirmed our postulated binding modes within these kinases. Taken together, small molecule kinase inhibitor **11b** can be suggested for the use as a pharmacological tool to further investigate the significance of CK1δ in signaling pathways, especially linked to proliferative and neurodegenerative diseases.

## Figures and Tables

**Table 1:** Synthesized test compounds of series 1, 2, 3, and 4. R refers to the side chains indicated in Scheme 5. Only a limited selection of thioether compounds has been oxidized leading to sulfoxide compounds (**12h**–**12m**, **16c**). Abbreviation: # compound number.

Thioether #	Sulfoxide #	R	Thioether #	Sulfoxide #	R
**10a**		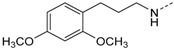	**11c**		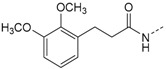
**10b**		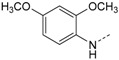	**11d**		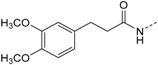
**10c**	**10j**	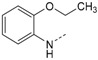	**12a**	**12h**	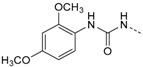
**10d**	**10k**	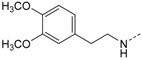	**12b**		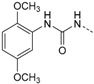
**10e**		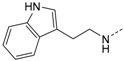	**12c**	**12i**	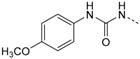
**10f**		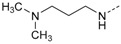	**12d**	**12j**	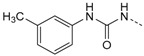
**10g**		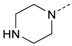	**12e**	**12k**	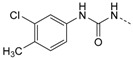
**10h**		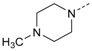	**12f**	**12l**	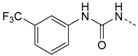
**10i**		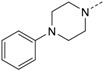	**12g**	**12m**	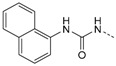
**11a**	**11f**	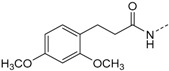	**16a**	**16c**	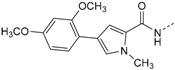
**11b**	**11g**	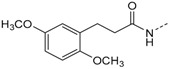	**16b**		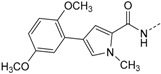

**Table 2:** IC_50_ values of promising inhibitors for CK1δ and CK1ε. All compounds have initially been screened in an in vitro assay against CK1δ and CK1ε at a concentration of 10 µM. The most promising agents were subjected to IC_50_ value determination. Results are presented as mean ± SD from experiments performed in triplicate (*n* = 3). Abbreviation: # compound number.

Compound #	CK1δ IC_50_ (nM)	CK1ε IC_50_ (nM)
**10a**	386 ± 86	6731 ± 2680
**10d**	644 ± 274	3323 ± 1297
**10e**	344 ± 104	1753 ± 738
**11a**	20 ± 2	129 ± 22
**11b**	4 ± 1	25 ± 4
**11c**	14 ± 2	91 ± 12
**11d**	20 ± 4	233 ± 38
**11e**	27 ± 3	204 ± 32
**11f**	93 ± 12	499 ± 70
**11g**	87 ± 2	573 ± 104
**12a**	19 ± 3	227 ± 37
**12b**	31 ± 3	186 ± 28
**12d**	47 ± 6	272 ± 52
**12e**	35 ± 10	203 ± 67
**12f**	196 ± 22	498 ± 124
**12h**	153 ± 24	910 ± 18
**12j**	160 ± 26	804 ± 160
**12k**	115 ± 18	764 ± 207
**12l**	88 ± 14	623 ± 139
**12m**	48 ± 8	182 ± 39
**16a**	9 ± 1	45 ± 7
**16b**	8 ± 1	81 ± 15
**16c**	32 ± 4	181 ± 32

**Table 3:** IC_50_ values of key inhibitors for CK1δ and p38α MAPK. IC_50_ values of the most promising agents of series 2 (**11b**), series 3 (**12a**), and series 4 (**16b**) have commercially been obtained from ProQinase GmbH (Freiburg, Germany). Single experiments have been performed (*n* = 1). Abbreviation: # compound number.

Compound #	CK1δ IC_50_ (nM)	p38α IC_50_ (nM)
**11b**	<3	10
**12a**	10	28
**16b**	<3	10

**Table 4:** EC_50_ values of selected inhibitor compounds as determined for Colo357, Panc89, Panc-1, and MiaPaCa-2 cell lines. Results are presented as mean ± SD from experiments performed in triplicate (*n* = 3). Abbreviations: # compound number; n.d., not determined.

Compound #	EC_50_ Values (µM)			
	Colo357	Panc89	MiaPaCa-2	Panc-1
**11a**	4.1 ± 1.7	2.5 ± 1	4.9 ± 0.8	7.0 ± 0.7
**11b**	3.5 ± 0.3	1.5 ± 0.4	n.d.	n.d.
**11c**	1.9 ± 0.6	6.1 ± 3.0	n.d.	n.d.
**11d**	5.9 ± 2.4	7.8 ± 0.4	n.d.	n.d.
**11e**	4.5 ± 1.3	8.3 ± 2.2	n.d.	n.d.
**12a**	17.4 ± 0.04	19.6 ± 3.8	15.22 ± 0.04	16.1 ± 5.6
**12b**	15.5 ± 1.6	n.d.	11.6 ± 1.1	15.2 ± 2.6
**12e**	14.9 ± 2.6	7.5 ± 3.0	n.d.	n.d.
**16a**	n.d.	n.d.	2.4 ± 0.7	5.1 ± 1.6
**16b**	5.7 ± 2.2	7.3 ± 1.9	2.3 ± 0.2	5.4 ± 1.5
**16c**	16.7 ± 5.6	n.d.	7.7 ± 2.5	10.2 ± 3.2

## Supplementary Materials

Supplementary materials are available online.

## Abbreviations

The following abbreviations are used in this manuscript:

AD	Alzheimer’s disease
ALS	amyotrophic lateral sclerosis
ATP	adenosine triphosphate
B-Raf	rapidly accelerated fibrosarcoma B
Boc	*tert*-butyloxycarbonyl
CDI	*N*,*N’*-carbonyldiimidazole
CK1	protein kinase CK1, formerly known as casein kinase 1
DCM	dichloromethane
DFG-in/out	sequence motif of aspartic acid (D), phenylalanine (F), and glycine (G) within the activation loop of the kinase domain
DIPEA	*N*,*N*-diisopropylethylamine
DMEM	Dulbecco’s modified Eagle’s medium
DMF	*N*,*N*-dimethylformamide
DMSO	dimethyl sulfoxide
EDTA	ethylenediaminetetraacetic acid
FASPS	familial advanced sleep phase syndrome
FCS	fetal calf serum
GST	glutathione S-transferase
HPI	hydrophobic pocket I
HRII	hydrophobic region II
JNK	c-Jun N-terminal kinase
LB	lysogeny broth
LCK	lymphocyte-specific protein tyrosine kinase
MAPK	mitogen-activated protein kinase
MTT	3-(4,5-dimethylthiazol-2-yl)-2,5-diphenyltetrazolium bromide
NaHMDS	sodium bis(trimethylsilyl)amide
NBS	*N*-bromosuccinimide
NTA	nitrilotriacetic acid
PBS	phosphate-buffered saline
Pd/C	palladium on activated charcoal
pdb	Research Collaboratory for Structural Bioinformatics (RCSB) protein data bank
PEG	polyethylene glycol
PPh_3_	triphenylphosphine
PyBOP	(Benzotriazol-1-yloxy)tripyrrolidinophosphonium hexafluorophosphate
rt	room temperature
RIPK	receptor-interacting Ser/Thr-protein kinase
RPMI	Roswell Park Memorial Institute medium
SAR	structure-activity relationship
SDS-PAGE	sodium dodecyl sulfate polyacrylamide gel electrophoresis
TBS	Tris-buffered saline
TCEP	tris(2-carboxyethyl)phosphine
THF	tetrahydrofuran
Tris	tris(hydroxymethyl)aminomethane
TTBK	tau tubulin kinase
VRK	vaccinia-related kinase
