# Supplementary material for: Optimized 4,5-Diarylimidazoles as Potent/Selective Inhibitors of Protein Kinase CK1δ and Their Structural Relation to p38α MAPK
Source: Molecules. 2017 Mar 24;22(4):522. doi: 10.3390/molecules22040522 (PMC6154583; doi:10.3390/molecules22040522)
Supplement: Supplementary file 1 [file molecules-22-00522-s001.pdf]

Supplementary Materials: Optimized 4,5-Diaryl-imidazoles as Potent/Selective Inhibitors of Protein Kinase CK1δ and Their Structural Relation to p38α MAPK

Jakob Halekotte, Lydia Witt, Chiara Ianes, Marc Krüger, Mike Bührmann, Daniel Rauh, Christian Pichlo, Ulrich Baumann, Uwe Knippschild, Joachim Bischof, and Christian Peifer

Table S1	HPLC methods used for the determination of key compound purity	**S2**
Table S2	IR data and purity of key compounds	**S3**
Table S3	*In vitro* dose-response data	**S5**
Figure S1	Initial cell culture screening of compound series 1	**S7**
Table S4	X-ray crystal structure analysis and CCDC-No. of compound 11b	**S8**
Table S4	Selectivity profile of compound 11b	**S9**
Table S5	Data collection, structure refinement, and Ramachandran plot results of protein crystallization	**S19**

Experimental data: Synthetic procedure and spectroscopic details for compound 10a **S20**

Table S1. HPLC methods used for the determination of key compound purity. All key compounds submitted to biological assays were proven to show ≥ 98 % purity.

Hewlett Packard 1050 Series			
Column specification: ZORBAX® Eclipse XDB-C8 (150 ·4.6 mm, 5 µm)			
Detection:	λ = 254 nm		
Temperature:	rt		
Injection volume:	20 µl		
Flow rate:	1.5 ml · min^{-1}		
Mobile phase A:	methanol		
Mobile phase B:	10 mM KH_2PO_4 buffer pH 2.3		
Gradients:	**Method 1**	Time 0 min:	A/B (40/60)
		Time 8 min:	A/B (85/15)
		Time 13 min:	A/B (85/15)
		Time 14 min:	A/B (40/60)
		Time 16 min:	A/B (40/60)
	**Method 2**	Time 0 min:	A/B (20/80)
		Time 8 min:	A/B (85/15)
		Time 13 min:	A/B (85/15)
		Time 14 min:	A/B (20/80)
		Time 15 min:	A/B (20/80)
	**Method 3**	Time 0 min:	A/B (40/60)
		Time 8 min:	A/B (85/15)
		Time 11 min:	A/B (85/15)
		Time 12 min:	A/B (40/60)
		Time 14 min:	A/B (40/60)

Table S2. IR data and purity of key compounds.

#	ATR-IR, $\tilde{\nu}$ (cm^{-1})	Purity (%)	HPLC method
**3**	3100, 2490, 1684, 1614, 1597, 1568, 1553, 1508, 1483, 1412, 1341, 1306, 1292, 1271, 1234, 1206, 1161, 118, 1115, 1103, 999, 964, 952, 921, 872, 853, 829, 812, 793, 760, 627, 582, 559	100	1
**4**	2860, 2373, 1667, 1593, 1543, 1504, 1417, 1289, 1262, 1233, 1192, 1153, 1074, 995, 945, 893, 868, 839, 752, 733, 694, 663, 637, 617, 588	98	1
**5**	2810, 2361, 1694, 1616, 1597, 1560, 1521, 1508, 1419, 1281, 1256, 1238, 1161, 1007, 974, 945, 849, 835, 792, 725, 681, 652, 606, 565, 554	98	1
**6**	2980, 1613, 1553, 1506, 1423, 1292, 1238, 1219, 1157, 1098, 1003, 982, 885, 868, 851, 829, 816, 702, 679, 635, 617, 586	100	1
**10a**	-	100	2
**10b**	-	97	1
**10c**	-	100	1
**10d**	-	100	1
**10e**	-	100	1
**10f**	-	100	1
**10g**	-	100	1
**10h**	-	95	1
**10i**	-	100	1
**10j**	-	100	1
**10k**	-	92	1
**11a**	2935, 1670, 1609, 1547, 1505, 1414, 1289, 1262, 1221, 1207, 1153, 1121, 1036, 835	98	1
**11b**	2925, 2400, 1668, 1607, 1551, 1499, 1416, 1221, 1157, 1045, 837, 814, 708	100	2
**11c**	-	100	2
**11d**	-	97	2
**11e**	-	99	2
**11f**	2955, 1701, 1609, 1573, 1545, 1528, 1506, 1412, 1287, 1221, 1209, 1153, 1121, 1034, 986, 831, 706	99	1
**11g**	2960, 1705, 1609, 1557, 1497, 1454, 1437, 1416, 1296, 1283, 1236, 1219, 1184, 1168, 1138, 1113, 1047, 1026, 876, 843, 816, 706	100	3

**12a**	3220, 1663, 1614, 1582, 1572, 1541, 1520, 1456, 1366, 1279, 1207, 1155, 1051, 1036, 837, 824, 787, 763, 669, 613	100	3
**12b**	3098, 2960, 2832, 1672, 1599, 1574, 1539, 1506, 1489, 1462, 1368, 1304, 1279, 1244, 1221, 1198, 1165, 1140, 1047, 1026, 976, 868, 845, 814, 743, 712	100	2
**12c**	3400, 1668, 1607, 1574, 1541, 1510, 1466, 1418, 1370, 1296, 1244, 1223, 1030, 843, 822, 750	99	3
**12d**	3358, 3200, 2980, 1674, 1607, 1581, 1549, 1518, 1476, 1462, 1366, 1294, 1219, 1207, 1157, 1094, 843, 810, 770, 737, 725, 679, 611	100	3
**12e**	3400, 2920, 1672, 1605, 1543, 1520, 1499, 1464, 1364, 1368, 1302, 1265, 1272, 1159, 880, 860, 839, 816, 754	98	3
**12f**	3300, 2900, 1670, 1609, 1581, 1541, 1520, 1497, 1464, 1381, 1370, 1331, 1312, 1294, 1256, 1217, 1207, 1180, 1157, 1134, 1099, 1072, 986, 923, 889, 871, 839, 814, 791, 754, 694	98	3
**12g**	3040, 1686, 1614, 1601, 1571, 1497, 1481, 1362, 1346, 1304, 1225, 976, 837, 791, 770, 739, 716, 683	100	3
**12h**	2950, 1657, 1603, 1564, 1547, 1508, 1454, 1360, 1298, 1281, 1215, 1179, 1155, 1132, 1051, 1032, 978, 837, 824, 750	100	3
**12i**	2950, 1661, 1603, 1568, 1557, 1508, 1471, 1298, 1238, 1209, 1180, 1161, 1058, 976, 876, 835, 818, 789, 737, 718, 681	99	3
**12j**	2950, 1667, 1605, 1562, 1514, 1476, 1393, 1362, 1314, 1300, 1244, 1221, 1167, 1044, 976, 883, 842, 827, 810, 770, 745, 716, 689	99	3
**12k**	3000, 1668, 1597, 1551, 1520, 1497, 1476, 1398, 1366, 1312, 1217, 1049, 835, 808, 745	98	3
**12l**	2900, 1680, 1607, 1576, 1516, 1495, 1476, 1265, 1240, 1229, 1163, 1121, 1099, 1049, 997, 977, 922, 876, 835, 785, 745, 716, 694	96	3
**12m**	2920, 1668, 1601, 1574, 1552, 1472, 1407, 1362, 1346, 1304, 1267, 1240, 1213, 1157, 1053, 974, 873, 842, 792, 770, 754	100	3
**13**	3129, 2984, 1703, 1688, 1468, 1414, 1400, 1389, 1331, 1242, 1186, 1111, 1078, 1057, 1022, 926, 818, 785, 750	100	2
**14a**	1676, 1613, 1559, 1495, 1466, 1435, 1412, 1360, 1287, 1204, 1180, 1128, 1109, 1051, 1032, 947, 828, 756	100	2
**15a**	3316, 1678, 1611, 1578, 1516, 1449, 1379, 1290, 1242, 1206, 1161, 1144, 1051, 1032, 993, 936, 822, 797, 768, 725	99	2
**15b**	2940, 2832, 1667, 1603, 1553, 1505, 1462, 1437, 1410, 1352, 1300, 1273, 1258, 1219, 1202, 1172, 1130, 1113, 1071, 1045, 1024, 889, 856, 842, 822, 806, 737, 708. 663	100	2
**16a**	3380, 2950, 1641, 1611, 1547, 1518, 1505, 1464, 1408, 1393, 1304, 1285, 1256, 1217, 1204, 1155, 1038, 843, 829, 797, 737, 692	99	2
**16b**	2935, 2832, 1667, 1607, 1547, 1505, 1412, 1393, 1362, 1285, 1219, 1169, 1045, 1024, 837, 814, 739, 721, 654	100	2
**16c**	2960, 1685, 1609, 1520, 1506, 1480, 1412, 1395, 1300, 1285, 1254, 1230, 1206, 1157, 1092, 1057, 1030, 941, 862, 835, 791, 702, 627	95	2

Table S3. *In vitro* dose-response data for CK1δ and ε for all tested compounds. Inhibitor compounds were serially diluted from 10 μM to 5 nM and used in *in vitro* kinase reactions using either human GST-CK1δ transcription variant 1 or human GST-CK1ε as enzyme; α-casein was used as substrate. Kinase reactions were separated in SDS-PAGE and incorporation of labeled phosphate into α-casein was quantified by Cherenkov counting. Dose-response analyses were performed using GraphPad Prism 6, error bars represent the standard error of the mean. Dose-response curves are displayed for CK1δ (filled black circles) and CK1ε (filled grey circles).

10a

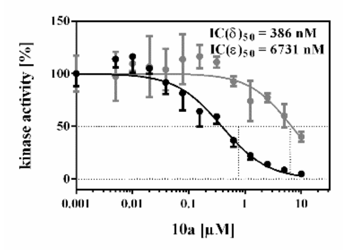

10d

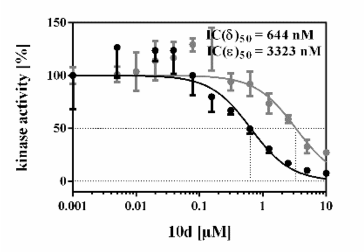

10e

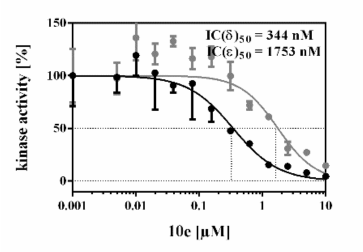

11a

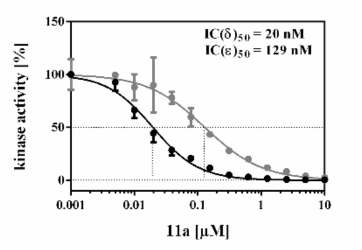

11b

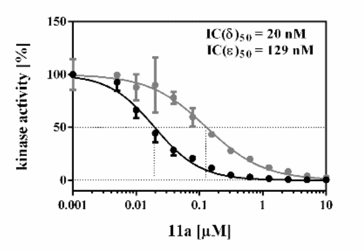

11c

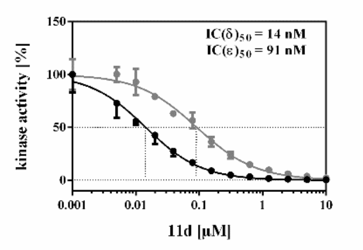

11d

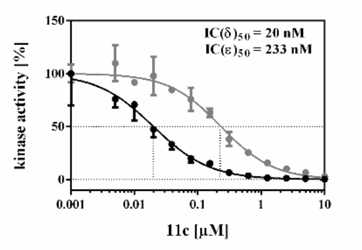

11e

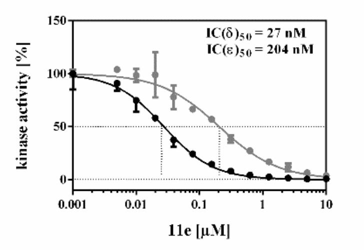

11f

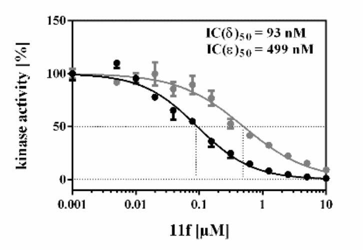

11g

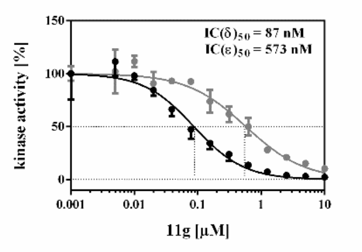

12a

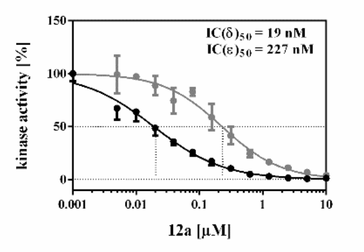

12b

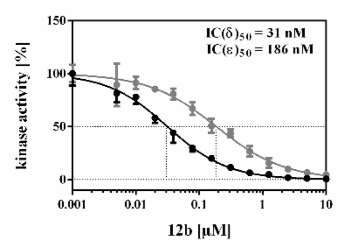

12d

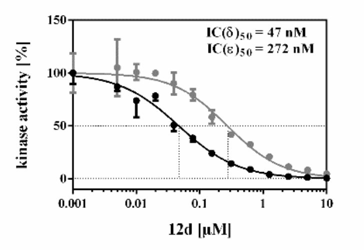

12e

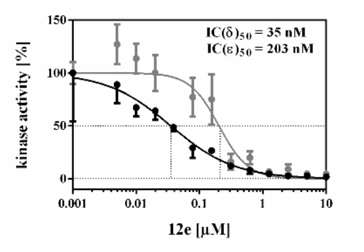

12f

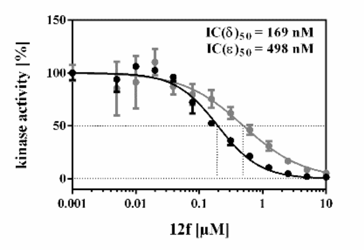

12h

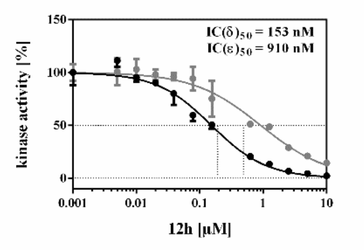

12i

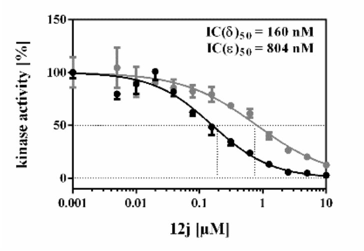

12k

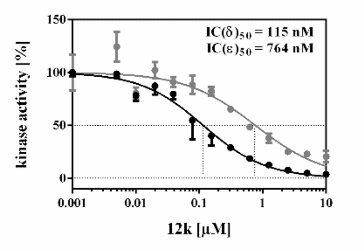

12l

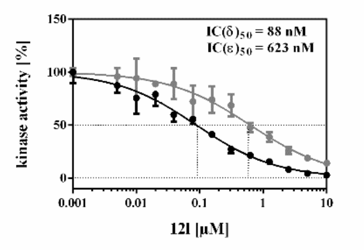

12m

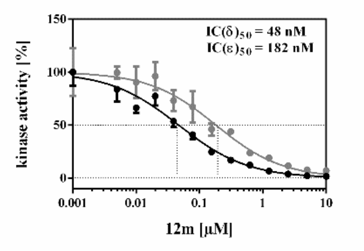

16a

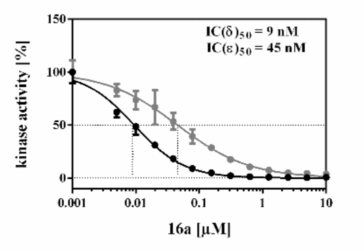

16b

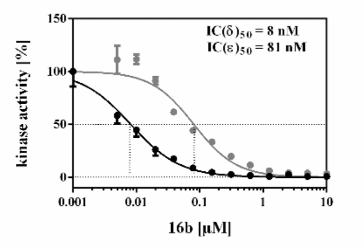

16c

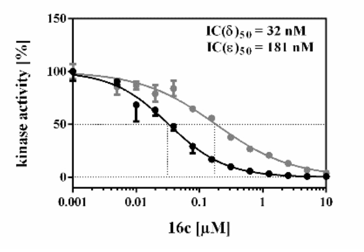

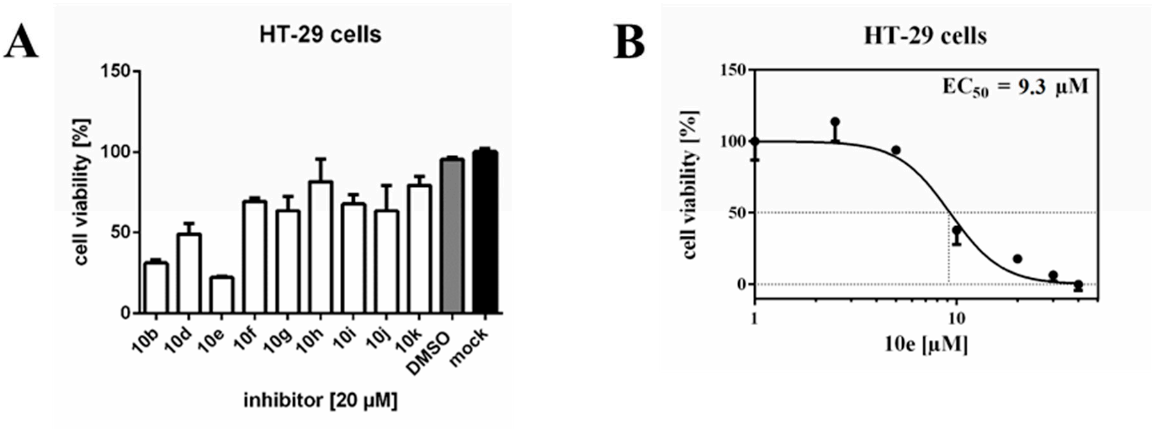

Figure S1. (**A**) HT-29 cells were treated with compounds of series 1 at 20 µM concentration and cell viability was measured by MTT viability assay. Error bars represent the standard error of the mean. (**B**) HT-29 cancer cells were treated with a dilution series of compound **10e** which showed most promising results in the previous screening at 20 µM concentration. Cell viability was determined by MTT assay and the EC_{50} value was calculated using GraphPad Prism 6. Error bars represent the standard error of the mean.

Table S4. X-ray crystal structure analysis and CCDC-No. Of compound **11b**.

Platon* plot showing the numbering scheme<br>*Spek, A. L. (2003). J. Appl. Cryst. 36, 7-13.	CCDC-No.
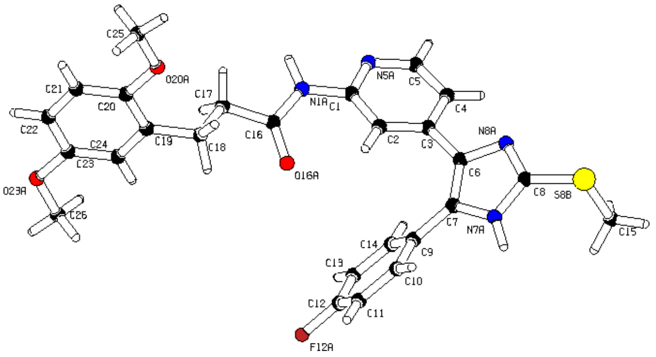	1517339

Table S5. Selectivity profile of compound **11b**. The inhibitor has been screened at a concentration of 100 nM over a panel of 320 wild-type protein kinases and B-Raf V600E by ProQinase GmbH (Freiburg, Germany) using an activity-based radiometric [33]PanQinase® assay. Results are presented as percentage of residual kinase activity relative to control. The final DMSO concentration was 1 % in each reaction-mix. Classification of protein kinase families refers to Manning *et al*. (Manning, G.; Whyte, D. B.; Martinez, R.; Hunter, T.; Sudarsanam, S. The Protein Kinase Complement of the Human Genome. Science **2002**, *298*, 1912–1934): AGC = containing PKA, PKG, PKC families; CAMK = containing Cdk, MAPK, GSK3, CLK families; STE = homologs of yeast sterile 7, sterile 11, sterile 20 kinases; TK = tyrosine kinase; TKL = tyrosine kinase-like.

Kinase name	Kinase family	Residual activity (%)
ABL1	TK	100
ABL2	TK	91
ACK1	TK	96
ACV-R1	TKL	95
ACV-R1B	TKL	101
ACV-R2A	TKL	98
ACV-R2B	TKL	97
ACV-RL1	TKL	99
AKT1 aa106-480	AGC	90
AKT2 aa107-481	AGC	97
AKT3 aa106-479	AGC	83
ALK (GST-HIS-tag)	TK	114
AMPK-alpha1 aa1-550	CAMK	96
ARK5	CAMK	88
ASK1	STE	93
Aurora-A	OTHER	93
Aurora-B	OTHER	92
Aurora-C	OTHER	93
AXL	TK	104
BLK	TK	77
BMPR1A	TKL	102
BMX	TK	98
B-RAF V600E	TKL	102
B-RAF	TKL	91
BRK	TK	98
BRSK1	CAMK	89

BRSK2	CAMK	105
BTK	TK	105
BUB1B	OTHER	102
CAMK1D	CAMK	78
CAMK2A	CAMK	95
CAMK2B	CAMK	103
CAMK2D	CAMK	96
CAMK2G	CAMK	105
CAMK4	CAMK	95
CAMKK1	OTHER	99
CAMKK2	OTHER	96
CDC42BPA	AGC	104
CDC42BPB	AGC	88
CDC7/ASK	OTHER	97
CDK1/CycA2	CMGC	91
CDK1/CycB1	CMGC	95
CDK1/CycE1	CMGC	97
CDK19/CycC	CMGC	86
CDK2/CycA2	CMGC	87
CDK2/CycE1	CMGC	99
CDK3/CycC	CMGC	95
CDK3/CycE1	CMGC	98
CDK4/CycD1	CMGC	98
CDK4/CycD3	CMGC	88
CDK5/p25NCK	CMGC	117
CDK5/p35NCK	CMGC	85
CDK6/CycD1	CMGC	105
CDK6/CycD3		97
CDK7/CycH/MAT1	CMGC	102
CDK8/CycC	CMGC	90
CDK9/CycK	CMGC	103
CDK9/CycT1	CMGC	93
CHK1	CAMK	92
CHK2	CAMK	98
CK1-alpha1	CK1	26

CK1-delta	CK1	3
CK1-epsilon	CK1	7
CK1-gamma1	CK1	100
CK1-gamma2	CK1	91
CK1-gamma3	CK1	73
CK2-alpha1	OTHER	97
CK2-alpha2	OTHER	90
CLK1	CMGC	105
CLK2	CMGC	101
CLK3	CMGC	98
CLK4	CMGC	95
COT	STE	111
CSF1-R	TK	104
CSK	TK	101
DAPK1	CAMK	96
DAPK2	CAMK	95
DAPK3	CAMK	85
DCAMKL2	CAMK	94
DDR2	TK	101
DMPK	AGC	103
DNA-PK	ATYP	95
DYRK1A	CMGC	96
DYRK1B	CMGC	101
DYRK2	CMGC	99
DYRK3	CMGC	97
DYRK4	CMGC	100
EEF2K	ATYPICAL	84
EGF-R	TK	113
EIF2AK2	OTHER	91
EIF2AK3	OTHER	91
EPHA1	TK	104
EPHA2	TK	95
EPHA3	TK	97
EPHA4	TK	96
EPHA5	TK	97

EPHA6	TK	100
EPHA7	TK	99
EPHA8	TK	115
EPHB1	TK	96
EPHB2	TK	55
EPHB3	TK	92
EPHB4	TK	97
ERBB2	TK	97
ERBB4	TK	98
ERK1	CMGC	94
ERK2	CMGC	103
ERK5	CMGC	95
ERK7	CMGC	93
FAK aa2-1052	TK	105
FER	TK	93
FES	TK	91
FGF-R1	TK	113
FGF-R2	TK	101
FGF-R3	TK	99
FGF-R4	TK	86
FGR	TK	103
FLT3	TK	109
FRK	TK	93
FYN	TK	119
GRK2	AGC	113
GRK3	AGC	91
GRK4	AGC	86
GRK5	AGC	97
GRK6	AGC	100
GRK7	AGC	83
GSG2	OTHER	121
GSK3-alpha	CMGC	107
GSK3-beta	CMGC	89
HCK	TK	58
HIPK1	CMGC	102

HIPK2	CMGC	92
HIPK3	CMGC	94
HIPK4	CMGC	94
HRI	OTHER	97
IGF1-R	TK	114
IKK-alpha	OTHER	98
IKK-beta	OTHER	92
IKK-epsilon	OTHER	102
INS-R	TK	101
INSR-R	TK	92
IRAK1	TKL	99
IRAK4 (untagged)	TKL	98
ITK	TK	66
JAK1 aa850-1154	TK	107
JAK2	TK	93
JAK3	TK	88
JNK1	CMGC	103
JNK2	CMGC	21
JNK3	CMGC	37
KIT	TK	103
LCK	TK	26
LIMK1	TKL	96
LIMK2	TKL	100
LRRK2	TKL	94
LTK	TK	101
LYN	TK	105
MAP3K1	STE	99
MAP3K10	STE	63
MAP3K11	STE	87
MAP3K7/MAP3K7IP1	STE	60
MAP3K9	STE	97
MAP4K2	STE	96
MAP4K4	STE	97
MAP4K5	STE	94
MAPKAPK2	CAMK	90

MAPKAPK3	CAMK	108
MAPKAPK5	CAMK	112
MARK1	CAMK	106
MARK2	CAMK	94
MARK3	CAMK	94
MARK4	CAMK	97
MATK	TK	101
MEK1	STE	92
MEK2	STE	100
MEK5	STE	93
MEKK2	STE	97
MEKK3	STE	101
MELK	CAMK	97
MERTK	TK	85
MET	TK	105
MINK1	STE	89
MKK4	STE	103
MKK6 S207D/T211D**	STE	89
MKK7	STE	96
MKNK1	CAMK	99
MKNK2	CAMK	101
MLK4	TKL	94
MST1	STE	124
MST2	STE	107
MST3	STE	92
MST4	STE	101
mTOR	ATYPICAL	96
MUSK	TK	102
MYLK	CAMK	97
MYLK2	CAMK	100
MYLK3	CAMK	98
NEK1	OTHER	101
NEK11	OTHER	77
NEK2	OTHER	103
NEK3	OTHER	93

NEK4	OTHER	89
NEK6	OTHER	99
NEK7	OTHER	113
NEK9	OTHER	98
NIK	STE	88
NLK	CMGC	78
p38-alpha	CMGC	22
p38-beta	CMGC	52
p38-delta	CMGC	99
p38-gamma	CMGC	99
PAK1	STE	93
PAK2	STE	121
PAK3	STE	100
PAK4	STE	101
PAK6	STE	103
PAK7	STE	129
PASK	CAMK	98
PBK	OTHER	121
PCTAIRE1/CycY	CMGC	148
PDGFR-alpha	TK	102
PDGFR-beta	TK	99
PDK1	AGC	106
PHKG1	CAMK	91
PHKG2	CAMK	99
PIM1	CAMK	95
PIM2	CAMK	99
PIM3	CAMK	102
PKA	AGC	100
PKC-alpha	AGC	99
PKC-beta1	AGC	99
PKC-beta2	AGC	106
PKC-delta	AGC	88
PKC-epsilon	AGC	98
PKC-eta	AGC	80
PKC-gamma	AGC	93

PKC-iota	AGC	98
PKC-mu	AGC	117
PKC-nu	AGC	72
PKC-theta	AGC	87
PKC-zeta	AGC	95
PKMYT1	OTHER	112
PLK1	OTHER	106
PLK3	OTHER	97
PRK1	AGC	108
PRK2	AGC	78
PRKD2	CAMK	104
PRKG1	AGC	94
PRKG2	AGC	87
PRKX	AGC	100
PYK2	TK	101
RAF1 Y340D/Y341D (untagged)**	TKL	84
RET	TK	113
RIPK2	TKL	45
RIPK5	TKL	97
ROCK1	AGC	92
ROCK2	AGC	99
RON	TK	110
ROS	TK	97
RPS6KA1	AGC	102
RPS6KA2	AGC	95
RPS6KA3	AGC	94
RPS6KA4	AGC	110
RPS6KA5	AGC	96
RPS6KA6	AGC	141
S6K	AGC	111
S6K-beta	AGC	98
SAK	OTHER	103
SGK1	AGC	111
SGK2	AGC	99
SGK3	AGC	90

SIK1	CAMK	101
SIK2	CAMK	117
SIK3	CAMK	96
SLK	STE	98
SNARK	CAMK	92
SNK	OTHER	94
SRC (GST-HIS-tag)	TK	100
SRMS	TK	117
SRPK1	CMGC	104
SRPK2	CMGC	105
STK17A	CAMK	98
STK23	CAMK	104
STK25	STE	96
STK33	CAMK	90
STK39	STE	109
SYK aa1-635	TK	108
TAOK2	STE	86
TAOK3	STE	89
TBK1	OTHER	96
TEC	TK	100
TGFB-R1	TKL	100
TGFB-R2	TKL	99
TIE2	TK	71
TLK1	AGC	107
TLK2	AGC	122
TNK1	TK	99
TRK-A	TK	101
TRK-B	TK	117
TRK-C	TK	116
TSF1	OTHER	99
TSK2	CAMK	93
TSSK1	CAMK	97
TTBK1	CK1	96
TTBK2	CK1	98
TTK	OTHER	94

TXK	TK	95
TYK2	TK	84
TYRO3	TK	94
VEGF-R1	TK	98
VEGF-R2	TK	90
VEGF-R3	TK	96
VRK1	CK1	100
VRK2	CK1	104
WEE1	OTHER	94
WNK1	OTHER	96
WNK2	OTHER	101
WNK3	OTHER	101
YES	TK	100
ZAK	TKL	96
ZAP70	TK	81

Table S6. Data collection, structure refinement, and Ramachandran plot results of protein crystallization. Crystallographic statistics of p38α MAPK in complex with **11b** (pdb 5ML5) and CK1δ in complex with **16b** (pdb 5MQV) are given. Values in parenthesis are for the highest resolution shell.

	**p38α MAPK with 11b**	**CK1δ with 16b**
Data collection		
Space group	$P2_12_12_1$	C 1 2 1
Cell dimensions		
a, b, c, (Å)	66.50, 69.39, 74.14	197.93, 127.28, 154.78
α, β, γ (°)	90, 90, 90	90, 113.63, 90
Resolution (Å)	50.0 – 1.90 (1.95 – 1.90)	49.29 - 2.15 (2.231 - 2.154)
R_{meas} (%)	9.0 (82.0)	6.83 (51.3)
I/σI	22.69 (3.38)	14.76 (2.49)
Completeness (%)	100 (100)	99 (94)
Redundancy	12.7 (12.9)	4.5 (4.3)
Refinement		
Resolution (Å)	48.01 – 1.90	49.29 - 2.15
No. reflections	26280	187579
R_{work}/R_{free}	19.5/23.9	17.3/19.2
No. atoms		
Protein	2623	14875
Ligand/ion	95	339
Water	103	697
B-factors		
Protein	26.63	51.17
Ligand/ion	36.87	64.20
Water	26.63	46.49
rms deviations		
Bond lengths (Å)	0.019	0.003
Bond angles (°)	2.029	0.62
Wavelength (Å)	1.0000	1.0000
Temperature (K)	90	90
X-ray source	PX II at SLS, Villigen, CH	PX III at SLS, Villigen, CH
Detector	Pilatus 6M-F	Pilatus 2M-F
Ramachandran plot		
Residues in		
favored regions	97.8 %	97 %
allowed regions	2.2 %	2.7 %
outlier regions	0 %	0 %

Experimental Data

Synthetic procedure and spectroscopic details for compound 10a

tert-Butyl (4-methylpyridin-2-yl)carbamate

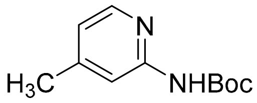

2-Amino-4-methylpyridine (3.00 g, 27.7 mmol) and Boc_2O (6.70 g, 30.5 mmol) were stirred in 70 ml *tert*-butanol at rt for 12 h. The solvent was removed under reduced pressure and the crude product was purified by flash chromatography (SiO_2, 10-50 % ethyl acetate/petrol ether) and crystallized from 2-propanol to afford the title compound as colorless crystals. Yield 5.35 g (93 %); $C_{11}H_{16}N_2O_2$ (M_r 208.26); mp: 125 °C; ^{1}H NMR ($CDCl_3$): δ = 1.53 (s, 9 H, ^{t}Bu), 2.35 (s, 3 H, C*H*$_3$), 6.79 (dq, ^3J = 5.3 Hz, ^4J = 0.6 Hz, 1 H, C^5*H*, Pyr), 7.88 (m, 1 H, C^3*H*, Pyr), 8.13 (dd, ^3J = 5.2 Hz, ^5J = 0.5 Hz, 1 H, C^6*H*, Pyr), 8.84 (bs, 1 H, N*H*) ppm; ^{13}C NMR ($CDCl_3$): δ = 21.6 (*C*H$_3$), 28.5 (C(*C*H$_3$)$_3$), 81.1 (*C*(CH$_3$)$_3$), 113.2 (*C*^{3}H, Pyr), 119.6 (*C*^{5}H, Pyr), 146.6 (*C*^{6}H, Pyr), 150.5 (*C*O), 152.3 (*C*^{4}, Pyr), 152.8 (*C*^{2}, Pyr) ppm; MS (ESI, 70 eV) *m*/*z* 153 [MH$_2$-^{t}Bu]$^+$.

tert-Butyl (3-(2,4-dimethoxyphenyl)propyl)(4-methylpyridin-2-yl)carbamate

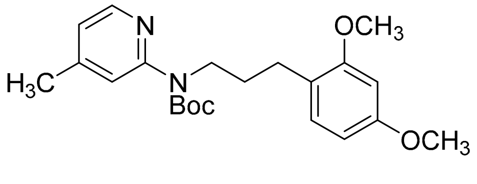

tert-Butyl (4-methylpyridin-2-yl)carbamate (138 mg, 663 µmol) was dissolved in 1.5 ml anhyd. DMF at 0 °C under a nitrogen atmosphere before NaH (39.0 mg 60 % dispersion in mineral oil, 975 µmol) in 2 ml anhyd. DMF was slowly added whereas the temp. was kept below 5 °C. The mixture was stirred at the same temp. for 20 min, **9** (144 mg, 556 µmol) in 3 ml anhyd. DMF was added, and stirring continued for another 30 min at 0 °C. The reaction was allowed to reach rt over 1 h, stirred for another 1 h, and quenched with H_2O. The mixture was extracted with ethyl acetate, washed with

0.1 M aq. HCl, sat. aq. $NaHCO_3$ solution, and sat. aq. NaCl solution, dried over anhyd. Na_2SO_4, and the solvent was removed under reduced pressure to afford the title compound as pale yellowish oil. Yield 202 mg (94 %); $C_{22}H_{30}N_2O_4$ (M_r 386.49); ^1H NMR ($CDCl_3$): δ = 1.48 (s, 9 H, ^{t}Bu), 1.79-1.90 (m, 2 H, $CH_2CH_2CH_2NR_2$), 2.33 (s, 3 H, CH_3), 2.54 (t, ^3J = 7.8 Hz, 2 H, $CH_2CH_2CH_2NR_2$), 3.75 (s, 3 H, C^2OCH_3), 3.77 (s, 3 H, C^4OCH_3), 3.95 (t, ^3J = 7.4 Hz, 2 H, $CH_2CH_2CH_2NR_2$), 6.38 (dd, ^3J = 8.0 Hz, ^4J = 2.5 Hz, 1 H, C^5H, $(OCH_3)_2$-Phe), 6.41 (d, ^4J = 2.3 Hz, 1 H, C^3H, $(OCH_3)_2$-Phe), 6.83 (dq, ^3J = 5.1 Hz, ^4J = 0.7 Hz, 1 H, C^5H, Pyr), 6.99 (dd, ^3J = 8.0 Hz, ^5J = 0.3 Hz, 1 H, C^6H, $(OCH_3)_2$-Phe), 7.39 (m, 1 H, C^3H, Pyr), 8.23 (dd, ^3J = 5.1 Hz, ^5J = 0.5 Hz, 1 H, C^6H, Pyr) ppm; ^{13}C NMR ($CDCl_3$): δ = 21.2 (CH_3), 27.1 ($CH_2CH_2CH_2NR_2$), 28.4 ($C(CH_3)_3$), 29.2 ($CH_2CH_2CH_2NR_2$), 47.0 ($CH_2CH_2CH_2NR_2$), 55.3 (C^2OCH_3), 55.5 (C^4OCH_3), 80.7 ($C(CH_3)_3$), 98.5 (C^3H, $(OCH_3)_2$-Phe), 103.8 (C^5H, $(OCH_3)_2$-Phe), 121.0 (C^5H, Pyr), 121.0 (C^3H, Pyr), 122.9 (C^1, $(OCH_3)_2$-Phe), 129.9 (C^6H, $(OCH_3)_2$-Phe), 147.4 (C^6H, Pyr), 148.1 (C^4, Pyr), 154.5 (CO), 154.9 (C^2, Pyr), 158.4 (C^2OCH_3), 159.2 (C^4OCH_3) ppm; MS (ESI, 70 eV) *m/z* 387 $[MH]^+$.

tert-Butyl (3-(2,4-dimethoxyphenyl)propyl)(4-(2-(4-fluorophenyl)-2-oxoethyl)-pyridin-2-yl)-carbamate

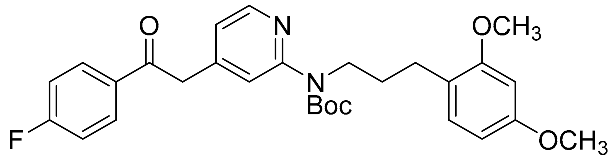

Synthesis was performed according to the procedure for **3** from *tert*-butyl (3-(2,4-dimethoxyphenyl)propyl)(4-methylpyridin-2-yl)carbamate (202 mg, 517 µmol), ethyl 4-fluorobenzoate (200 µl, 1.36 mmol) in 3 ml anhyd. THF, and NaHMDS (500 µl 2 M solution in THF, 1.00 mmol), but required further purification by flash chromatography (SiO_2, 5-35 % ethyl acetate/petrol ether) to afford the title compound as yellowish oil. Yield 126 mg (48 %); $C_{29}H_{33}FN_2O_5$ (M_r 508.59); ^1H NMR ($CDCl_3$): δ = 1.47 (s, 9 H, ^{t}Bu), 1.85-1.90 (m, 2 H, $CH_2CH_2CH_2NR_2$), 2.55 (t, ^3J = 7.7 Hz, 2 H, $CH_2CH_2CH_2NR_2$), 3.75 (s, 3 H, C^2OCH_3), 3.77 (s, 3 H, C^4OCH_3), 3.97 (t, ^3J = 7.5 Hz, 2 H, $CH_2CH_2CH_2NR_2$), 4.24 (s, 2 H, CH_2), 6.38 (dd, ^3J = 8.0 Hz, ^4J = 2.5 Hz, 1 H, C^5H, $(OCH_3)_2$-Phe), 6.41 (d, ^4J = 2.3 Hz, 1 H, C^3H, $(OCH_3)_2$-Phe), 6.90 (dd, ^3J = 5.1 Hz, ^4J = 1.5 Hz, 1 H, C^5H,

Pyr), 6.99 (d, ^3J = 8.1 Hz, 1 H, C^6*H*, $(OCH_3)_2$-Phe), 7.10-7.16 (m, 2 H, C$^{3/5}$*H*, F-Phe), 7.57 (bs, 1 H, C^6*H*, Pyr), 7.99-8.04 (m, 2 H, C$^{2/6}$*H*, F-Phe), 8.32 (dd, ^3J = 5.1 Hz, ^5J = 0.6 Hz, 1 H, C^6*H*, Pyr) ppm; ^{13}C NMR ($CDCl_3$): δ = 27.1 ($CH_2CH_2CH_2NR_2$), 28.4 ($C(CH_3)_3$), 29.2 ($CH_2CH_2CH_2NR_2$), 44.9 (CH_2), 47.0 ($CH_2CH_2CH_2NR_2$), 55.3 (C^2OCH_3), 55.5 (C^4OCH_3), 81.0 (*C*$(CH_3)_3$), 98.5 (C^3H, $(OCH_3)_2$-Phe), 103.8 (C^5H, $(OCH_3)_2$-Phe), 116.1 (d, $^2J_{CF}$ = 22.0 Hz, *C*$^{3/5}$H, F-Phe), 120.6 (C^5H, Pyr), 120.8 (*C*^{6}H, Pyr), 122.8 (*C*^{1}, $(OCH_3)_2$-Phe), 129.9 (*C*^{6}H, $(OCH_3)_2$-Phe), 131.3 (d, $^3J_{CF}$ = 9.4 Hz, *C*$^{2/6}$H, F-Phe), 132.8 (d, $^4J_{CF}$ = 2.9 Hz, *C*^{1}, F-Phe), 144.3 (*C*^{4}, Pyr), 147.6 (C^3H, Pyr), 154.3 (*C*OO^{t}Bu), 155.1 (*C*^{2}, Pyr), 158.4 (*C*^{2}OCH$_3$), 159.2 (C^4OCH_3), 166.1 (d, $^1J_{CF}$ = 255.7 Hz, *C*F), 194.5 (*C*O) ppm; MS (ESI, 70 eV) *m*/*z* 509 $[MH]^+$.

tert-Butyl (3-(2,4-dimethoxyphenyl)propyl)(4-(2-(4-fluorophenyl)-1-(hydroxyimino)-2-oxoethyl)-pyridin-2-yl)carbamate

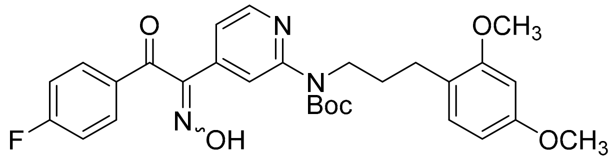

Synthesis was performed according to the procedure for **4** from *tert*-butyl (3-(2,4-dimethoxyphenyl)propyl)(4-(2-(4-fluorophenyl)-2-oxoethyl)-pyridin-2-yl)carbamate (110 mg, 216 μmol) in 3 ml glacial acetic acid, and $NaNO_2$ (45.0 mg, 652 μmol in 0.5 ml H_2O) to afford the title compound as brownish oil. Yield 114 mg (quant.); $C_{29}H_{32}FN_3O_6$ (M_r 537.59); ^{1}H NMR ($CDCl_3$): δ = 1.41 (s, 9 H, ^{t}Bu), 1.81-1.86 (m, 2 H, $CH_2CH_2CH_2NR_2$), 2.52 (t, ^3J = 7.7 Hz, 2 H, $CH_2CH_2CH_2NR_2$), 3.73 (s, 3 H, C^2OCH_3), 3.77 (s, 3 H, C^4OCH_3), 3.94 (t, ^3J = 7.4 Hz, 2 H, $CH_2CH_2CH_2NR_2$), 6.37 (dd, ^3J = 8.1 Hz, ^4J = 2.5 Hz, 1 H, C^5*H*, $(OCH_3)_2$-Phe), 6.40 (d, ^4J = 2.3 Hz, 1 H, C^3*H*, $(OCH_3)_2$-Phe), 6.96 (d, ^3J = 8.1 Hz, 1 H, C^6*H*, $(OCH_3)_2$-Phe), 7.09 (dd, ^3J = 5.3 Hz, ^4J = 1.5 Hz, 1 H, C^5*H*, Pyr), 7.12-7.18 (m, 2 H, C$^{3/5}$*H*, F-Phe), 7.77 (bs, 1 H, C^3*H*, Pyr), 7.93-7.98 (m, 2 H, C$^{2/6}$*H*, F-Phe), 8.35 (dd, ^3J = 5.3 Hz, ^5J = 0.8 Hz, 1 H, C^6*H*, Pyr), 9.01 (vbs, 1 H, CNO*H*) ppm; ^{13}C NMR ($CDCl_3$): δ = 27.0 ($CH_2CH_2CH_2NR_2$), 28.3 ($C(CH_3)_3$), 29.1 ($CH_2CH_2CH_2NR_2$), 47.2 ($CH_2CH_2CH_2NR_2$), 55.3 (C^2OCH_3), 55.5 (C^4OCH_3), 81.5 ($C(CH_3)_3$), 98.6 (C^3H, $(OCH_3)_2$-Phe), 103.9 (C^5H, $(OCH_3)_2$-Phe), 116.2 (C^5H, Pyr), 116.6 (d, $^2J_{CF}$ = 22.4 Hz, *C*$^{3/5}$H, F-Phe), 116.8 (C^3H, Pyr), 122.7 (*C*^{1}, $(OCH_3)_2$-Phe), 129.9 (C^6H, $(OCH_3)_2$-Phe), 131.2

(d, ${}^{4}J_{CF}$ = 2.9 Hz, C^1, F-Phe), 132.4 (d, ${}^{3}J_{CF}$ = 9. 8 Hz, $C^{2/6}$H, F-Phe), 139.8 (C^4, Pyr), 148.3 (C^6H, Pyr), 154.1 (*C*OO^{t}Bu), 154.8 (*C*NOH), 155.5 (C^2, Pyr), 158.4 (C^2OCH_3), 159.2 (C^4OCH_3), 166.9 (d, ${}^{1}J_{CF}$ = 258.0 Hz, *C*F), 191.8 (*C*O) ppm; MS (ESI, 70 eV) *m*/*z* 538 [MH]$^+$.

1-(2-((3-(2,4-Dimethoxyphenyl)propyl)amino)pyridin-4-yl)-2-(4-fluorophenyl)-2-oxoethan-1-aminium chloride

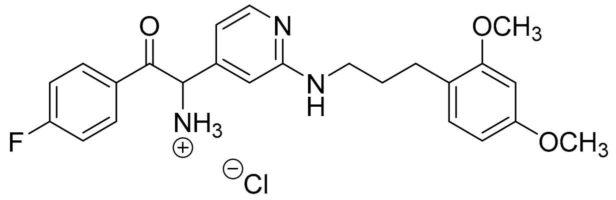

Synthesis was performed according to the procedure for **5** from *tert*-butyl (3-(2,4-dimethoxyphenyl)propyl)(4-(2-(4-fluorophenyl)-1-(hydroxyimino)-2-oxoethyl)pyridin-2-yl)carbamate (110 mg, 205 μmol) in 1 ml 2-propanol and 1.5 ml HCl-sat. 2-propanol, and Pd/C 10 % (20 mg). The crude product was obtained by filtration, the residue was rinsed with methanol. The filtrate was concentrated under reduced pressure and the title compound was afforded by crystallization from diethyl ether as a colorless solid and directly used for the next step without complete spectroscopic characterization. Yield 94.0 mg (99 %); $C_{24}H_{27}ClFN_3O_3$ (M_r 459.95); MS (ESI, 70 eV) *m*/*z* 424 [M-Cl]$^+$.

N-(3-(2,4-Dimethoxyphenyl)propyl)-4-(5-(4-fluorophenyl)-2-(methylthio)-1H-imidazol-4-yl)-pyridin-2-amine (10a)

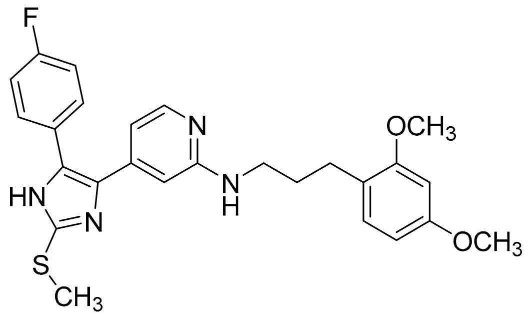

Synthesis was performed according to the procedure for **6** from 1-(2-((3-(2,4-dimethoxyphenyl)propyl)amino)pyridin-4-yl)-2-(4-fluorophenyl)-2-oxoethan-1-aminium chloride (90.0 mg, 196 µmol) in 3 ml anhyd. DMF, and methyl thiocyanate (50.0 µl, 725 µmol), but required further purification by flash chromatography (SiO_2, 20-100 % ethyl acetate/petrol ether and RP-18, 50-100 % methanol/H_2O). to afford **10a** as yellow solid. Yield 36.0 mg (38 %); $C_{26}H_{27}FN_4O_2S$ (M_r 478.59); ^1H NMR (DMSO-d_6): δ = 1.67-1.72 (m, 2 H, $CH_2CH_2CH_2NH$), 2.48-2.52 (m, 2 H, $CH_2CH_2CH_2NH$), 2.60 (s, 3 H, SCH_3), 3.13-3.17 (m, 2 H, $CH_2CH_2CH_2NH$), 3.72 (s, 3 H, C^4OCH_3), 3.74 (s, 3 H, C^2OCH_3), 6.41-6.50 (m, 5 H, $C^{3/5}H$, Pyr and $C^{3/5}H$, $(OCH_3)_2$-Phe and $CH_2CH_2CH_2NH$), 7.01 (d, ^3J = 8.2 Hz, 1 H, C^6H, $(OCH_3)_2$-Phe), 7.22 (m, 2 H, $C^{3/5}H$, F-Phe), 7.48 (m, 2 H, $C^{2/6}H$, F-Phe), 7.85 (bs, C^6H, Pyr), 12.58 (bs, 1H, NH) ppm; ^{13}C NMR (DMSO-d_6): δ = 15.1 (SCH_3), 26.6 ($CH_2CH_2CH_2NH$), 29.3 ($CH_2CH_2CH_2NH$), 40.7 ($CH_2CH_2CH_2NH$), 55.1 (C^4OCH_3), 55.2 (C^2OCH_3), 98.3 (C^3H, $(OCH_3)_2$-Phe), 104.3 (C^5H, $(OCH_3)_2$-Phe), 104.9 (C^3H, Pyr), 109.7 (C^5H, Pyr), 115.4 ($C^{3/5}H$, F-Phe), 121.8 (C^1, $(OCH_3)_2$-Phe), 126.5 (C^4, Imdz), 127.0 (C^5, Imdz), 129.7 (C^1, F-Phe), 129.7 (C^6H, $(OCH_3)_2$-Phe), 130.1 ($C^{2/6}H$, F-Phe), 142.1 (C^2, Imdz), 147.5 (C^6H, Pyr), 157.9 (C^2OCH_3), 158.8 (C^4OCH_3), 159.3 (C^2, Pyr) ppm; MS (ESI, 70 eV) m/z 479 $[MH]^+$; HRMS (EI, 70 eV) m/z $[M]^+$ calcd for $C_{26}H_{27}FN_4O_2S$, 478.1839; found, 478.1839.
